# Prediction of vegetation indices from down-sampled hyperspectral data using machine learning: A novel framework for olive crop monitoring

**DOI:** 10.1371/journal.pone.0323158

**Published:** 2026-03-27

**Authors:** Juan Estrada, Necati Cetin, Kamil Sacilik, Zhen Guo, Fernando Auat Cheein

**Affiliations:** 1 Department of Electronic Engineering, Universidad Tecnica Federico Santa Maria, Av. España, Valparaíso, Chile; 2 Faculty of Agriculture, Department of Agricultural Machinery and Technologies Engineering, Ankara University, Ankara, Turkey; 3 School of Agricultural Engineering and Food Science, Shandong University of Technology, Zhangdian District, Zibo, Shandong, China; 4 Department of Engineering, Harper Adams University, Newport, Shropshire, United Kingdom; 5 School of Mathematical and Computer Sciences, Heriot Watt University, Edinburgh, United Kingdom; Universidade Federal de Uberlandia, BRAZIL

## Abstract

Accurate plant health monitoring relies on hyperspectral imagery to extract vegetation spectral signatures and compute vegetation indices (VIs), which are critical for phenotyping and crop condition assessment. However, the requirement for high spectral resolution significantly increases the cost and complexity of data acquisition. In this study, we proposed a novel machine learning-based framework for predicting VIs from down-sampled hyperspectral reflectance data. The aim was to reduce the dependency on high-resolution spectral imagery without compromising prediction accuracy. The framework integrated correlation-based feature selection with four regression models to identify and utilize the most informative spectral bands from coarsely sampled data. The system was trained and validated using a data set consisting of 555 spectral signatures collected from olive leaves at five stages of dehydration, with spectral resolutions ranging from 1 to 100 nm. A total of 25 vegetation indices, commonly used in the estimation of water stress, chlorophyll, and nitrogen, were predicted on various sampling scales. Experimental results show that even with 100 nm spectral resolution, the proposed framework achieves high prediction accuracy, with coefficients of determination reaching 0.99 for RVSI, VOPT, and SPADI indices. These findings demonstrate that accurate vegetation index estimation is achievable with significantly fewer spectral bands, offering a cost-effective solution for large-scale plant health monitoring. This framework lays the groundwork for the development of low-cost, data-efficient remote sensing systems for precision agriculture, especially in crops such as olives, where health dynamics are sensitive to water and nutrient status.

## Introduction

Olive orchards are of great importance for agriculture in Mediterranean regions [1]. The country of Turkey produces over 200 thousand tons of olive oil and over 450 thousand tons of table olives each year, making it one of the largest producers in the world alongside other countries in the Mediterranean zone [1]. In Chile, even though it is not one of the largest producers worldwide, olive trees are among the most planted trees in the country, with over 20 thousand hectares of olive trees and a production of 14 thousand tons of olive oil for exportation in 2018 [2]. Due to the importance of olive orchards in the economy of various countries worldwide, methods for a quick assessment of the health status of vegetation can assist farmers and producers make better decisions about management and farming [3].

To evaluate the health status of vegetation, one of the most common approaches is to use a multi-spectral or hyperspectral camera mounted on a robotic platform such as an unmanned aerial vehicle (UAV) or a terrestrial vehicle to survey large areas of land [3,4]. The images are then used to create maps and compute vegetation indices (VIs). Vegetation indices derived from multi-spectral images are used in conjunction with machine learning algorithms to estimate the nitrogen and chlorophyll content of various crops, including maize [5], rice [6,7], cotton [8,9], onion [10], potato [11], wheat [12], sugarcane [13], corn [14]. Similar approaches have been tested for yield prediction of various crops using multi-spectral vegetation indices including ratoon rice [15], soybean [16], cotton [9], onion [10] and, sweet potato [17]. These applications highlight the potential of vegetation indices as universal indicators of plant health, especially when coupled with data-driven methods.

The detection of pests and vegetation diseases has been explored using multi-spectral information. For example, [18] used aerial multi-spectral images to distinguish healthy coffee leaves from those infested with the coffee leaf miner. In another study, the damage caused by nematodes in leaves is identified using vegetation indices derived from multi-spectral images [19]. In addition, more robust plant varieties resistant to diseases can be determined based on their spectral reflectance. For instance, [20] demonstrates the identification of resistant and susceptible varieties of sugarcane against brown and orange rust using multi-spectral images.

The use of multi-spectral imagery for various applications related to vegetation status is a highly researched topic. However, most commercial multi-spectral cameras only capture a limited number of wavelengths from the spectrum. In contrast, hyperspectral cameras and handheld spectrometers can record up to thousands of spectral bands [4]. Nevertheless, these devices often exhibit low spatial resolution [21], and handheld spectrometers are typically limited to recording information from a single point or pixel in space [4]. These practical limitations—high cost, restricted coverage, and complexity in deployment—present significant barriers to large-scale real-time monitoring.

Given the numerous applications of hyperspectral information in agriculture, there have been several efforts to predict spectral bands in the infrared regions using information from relatively inexpensive and standard sensors. For example, [22] explores the reconstruction of the near-infrared band and the red edge band with center wavelengths at 840 nm and 717 nm, respectively. They achieve this using images in the visible spectrum (red, green, and blue bands). For this purpose, they trained a hyperspectral deep convolutional neural network (HSCNN-R) [23] to reconstruct the multi-spectral bands with true and natural color images in the visible red, green, and blue (RGB) bands. Other studies involve the use of generative-adversarial networks (GAN) for the reconstruction of multi-spectral images and the prediction of vegetation indices [24].

In a similar study also conducted by [22], the same HSCNN-R network was trained to reconstruct 204 bands ranging from 397 nm to 1003 nm with a spectral resolution of 7 nm, from RGB images obtained through a smartphone camera. This approach was tested for quality analysis of tomatoes. Other approaches utilize shallow learning methods, mainly coupled dictionaries and sparse representation of matrices, for the prediction of multi-spectral bands while employing lighter models rather than deep learning networks [25].

Although previous studies are capable of reconstructing spectral bands from the infrared region, it is still limited to a few bands (Near Infrared Band) [22,23]; or to the region of the spectrum up to 1000 nm [22]. The latest approaches aim to reconstruct bands in the visible to near-infrared (VNIR) and region, which ranges from 350 nm to 1000 nm [26], but for applications such as moisture content prediction [27], the spectrum in the short-wave infrared (SWIR) region, ranging from 1000 to 2500 nm is needed.

Handheld devices such as spectrometers are capable of capturing spectral bands in the shortwave infrared region (SWIR), which ranges from 1001 nm to 2500 nm. In their work, [26] propose the reconstruction of the SWIR region using reflectance values in the visible to near-infrared (VNIR) region with machine learning algorithms. They conducted tests using a dataset composed of spectral signatures of *Eucalyptus globulus* leaves at different dehydration levels to predict vegetation indices related to the water content of leaves.

Another study conducted by [28] aims to predict water content indices from a single index. For this approach, the index with the highercoefficient of determination factor was chosen to develop linear models for the other 17 tested indices. All the models achieved a coefficient of determination over 0.7 and in some cases, it achieved a factor over 0.9. However, this approach performed worse when predicting indices not directly related to water content. Moreover, most of these approaches still require full-resolution hyperspectral acquisition prior to analysis, and the model architectures often target only specific indices or spectral zones, limiting generalizability and efficiency. The research focused on the reconstruction of spectral bands from incomplete information suggests that there is a fundamental set of wavelengths that allows the complete reconstruction of the spectrum with minimal information loss [26].

Therefore, this work aims to identify the minimum spectral resolution that allows the precise reconstruction of vegetation indices related to the health status of the plant. In contrast to prior work, our objective is not to reconstruct missing bands but to determine the lowest spectral resolution required for effective vegetation monitoring—before acquiring data so that lighter, more affordable systems can be designed. For this purpose, we down-sampled the spectral signature from 1 nm up to a 100 nm spectral resolution. With the down-sampled spectral, a correlation feature-selection algorithm was used to select the most relevant bands for each VI, and four machine-learning algorithms (random forest, artificial neural network, support vector machine, and k-nearest neighbors) were trained. Our approach is tested using a dataset composed of 555 spectral signatures from olive leaves at different dehydration levels, collected from the Valparaíso region in Chile. Together, these components form a novel framework for efficient, data-driven prediction of vegetation indices from reduced spectral inputs—enabling practical plant health monitoring at lower costs and complexity.

This work is organized as follows: Section 2 presents the system architecture used, including a description of the dataset and its construction, the main vegetation indices found in the literature for plant health evaluation, and the techniques used to identify the minimum set of bands required for index reconstruction. Section 3 presents the experimental results of the approach; Section 4 discusses the results, and finally, Section 5 presents the conclusion and future perspectives.

## Materials and methods

**Fig 1:**
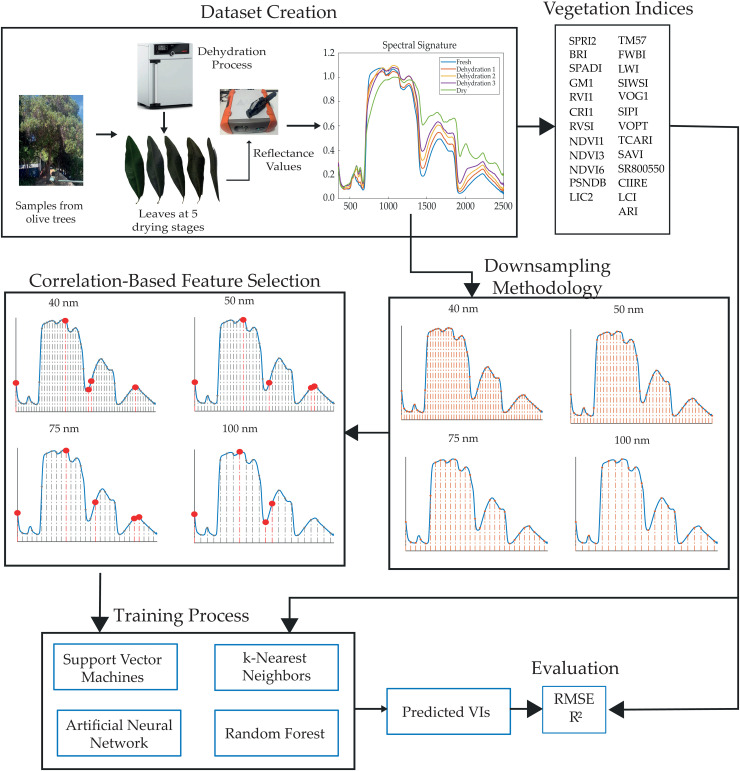
The architecture proposed in this work. Samples were collected from olive trees, and a dehydration process was conducted generating fresh, three dehydration and fully dried stages, the spectral signature was recorded and vegetation indices were computed. The spectral signature was downsampled to reduce the number of bands, a correlation-based feature selection was performed, and four machine learning algorithms were trained to predict the vegetation indices. Finally, the models were evaluated using RMSE and R^2^.

This section details the methods implemented in this study. Fig 1 provides an overview of the architecture used. The process begins with the creation of a dataset composed of leaves from olive trees, which were subjected to a dehydration process at five different stages. The spectral signature from 350 nm to 2500 nm was recorded using a spectrometer.

A set of 25 vegetation indices was computed from the spectral signature; these indices were chosen for their prominence in tasks such as leaf water content prediction [27], chlorophyll estimation [29], and general vegetation health assessment [30,31]. The spectral signature was down-sampled to obtain information at intervals of 5, 10, 20, 30, 40, 50, 75, and 100 nm. At a resolution of 100 nm, only 21 bands are preserved from the original 2151 bands, which is 1.02% of the original information.

Correlation-based feature selection was performed on the down-sampled data to identify the best predictors for each vegetation index at each dehydration stage. These features were used to train four machine learning regression algorithms: random forest, support vector machines, artificial neural network, and k-nearest neighbors. The models were evaluated using the root mean squared error and coefficient of determination.

### Dataset creation and curation

The dataset used in this study consists of 555 spectral signatures from 111 olive tree leaves at five different dehydration levels. The leaves were collected from trees located on the campus of Universidad Técnica Federico Santa María in the Valparaiso region of Chile. The sample collection process took place in August 2023.

The leaves were directly cut from tree branches and then placed in plastic bags to preserve their water moisture content. Within the next hour, the leaves were separated from the branches, following the recommendations of [27]. The spectral reflectance of each leaf was measured using a high-resolution ASD Terraspec4 spectrometer. The spectrometer was first calibrated by placing the probe flush against a white Spectralon reference background. Next, the samples were placed on the reference panel, and the probe was positioned flush against each sample.

After the initial set of measurements, the leaves were dried in a Memmert UN30 oven to introduce three dehydration stages at 65°C for 15, 30, and 45 minutes, respectively. This temperature was chosen based on common practices of combustion scientists [27], who typically dry samples at temperatures ranging from 60°C to 80°C. After the dehydration process, the spectral signature was measured again. This procedure was carried out three times, producing four sets of measurements: one at the fresh state of the leaves and three at different dehydration levels. Subsequently, the leaves were placed in the oven for 24 hours to remove any remaining water traces. After this period, the spectral signature was recorded once more.

**Fig 2:**
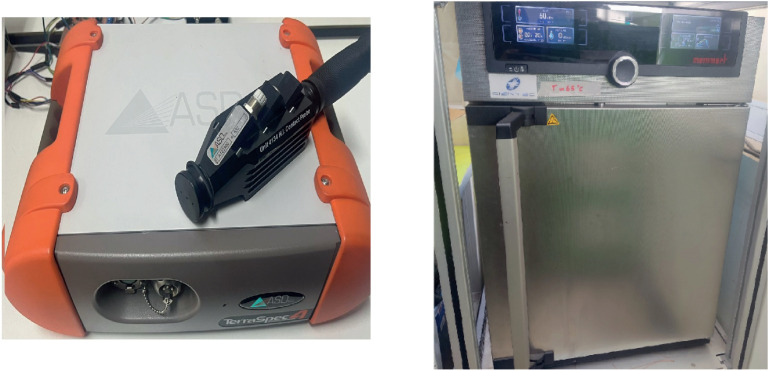
Instruments used to create the dataset. ADS Terraspec Hi-Res spectrometer (left). Memmert UN30 oven (right).

**Fig 3:**
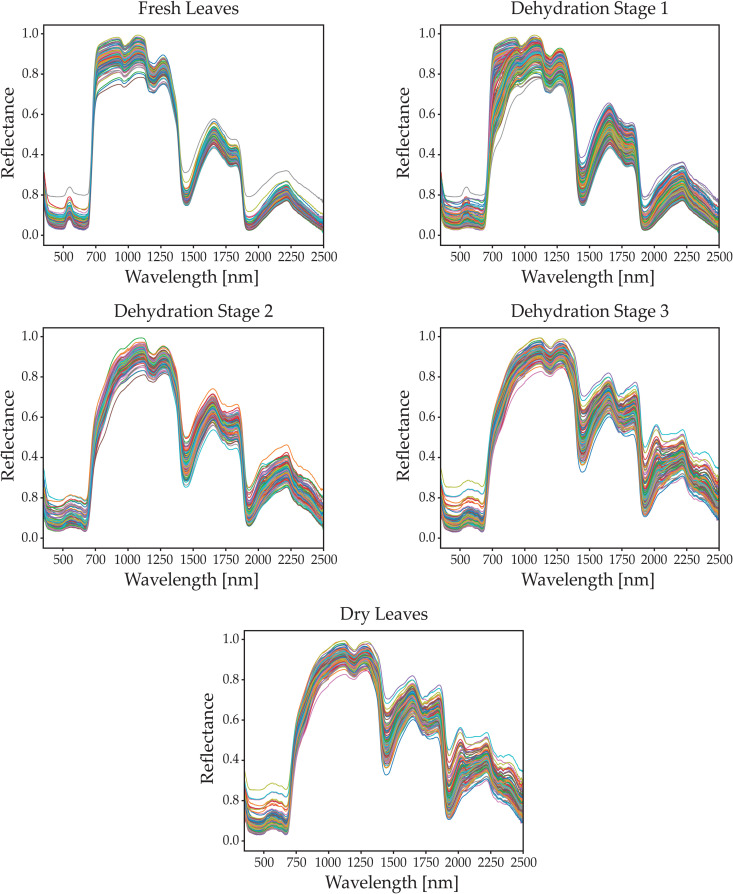
Resulting spectral signatures from olive leaves at different dehydration stages. Fresh leaves. First dehydration stage. Second dehydration stage. Third dehydration stage. Completely dry leaves.

The most important features of the equipment used to gather the information are presented in Table 1. The instruments are illustrated in Fig 2. The resulting spectral signatures are displayed in Fig 3, where each sub-figure represents a different dehydration stage.

**Table 1:** Equipment used to create the dataset.

Instrument	Technical Specification
ASD Terraspec 4 Hi-Res Spectrometer	Range: 350–2500 nm. Resolution: 3 nm at VNIR (350–1000 nm); 6 nm at SWIR (1001–2500 nm). Reproducibility: 0.1 nm. Accuracy: 0.5 nm.
Memmert UN30 Oven	Temperature: −5–100 °C with respect to room temperature. Digital PID control.

### Vegetation indices

Vegetation indices are a fundamental tool for the remote evaluation of vegetation. The electromagnetic reflectance of leaves or vegetation is highly influenced by their chemical and morphological features, such as water content, pigment content, and protein content, among others [32].

Selecting an appropriate vegetation index (VI) for a particular task is complex due to the wide variety of light spectra combinations, instrument resolution, and environmental conditions. For specific applications, VIs are selected using a tailored approach and validated with in situ measurements [32]. In the literature, various compilations of vegetation indices have been made for different purposes in agriculture [4,32–34].

This study focuses on the estimation of 25 vegetation indices, which have been tested for estimating leaf water content [26,27], leaf chlorophyll content [29], and nitrogen content [35]. Table 2 presents the vegetation indices used in this study and their formulas. In the table, *R*_*x*_ indicates the reflectance value at the *x* wavelength.

**Table 2:** Vegetation indices used in this study.

Name	Acronym	Equation	Reference
Theme Mapper 57	TM57	$R_{1650}/R_{2220}$	[27]
Fractional Water Band Index	FWBI	$R_{900}/\min(R_{930}-R_{980})$	[27]
Leaf Water Index	LWI	$R_{1300}/R_{1450}$	[27]
Shortwave Infrared Water Stress Index	SIWSI	$\frac{R_{1640}-R_{858}}{R_{1640}+R_{868}}$	[27]
Vogelmann Red Edge Index	VOG1	$R_{740}/R_{720}$	[29]
Structure Insensitive Pigment Index	SIPI	$\frac{R_{800}-R_{445}}{R_{800}+R_{680}}$	[29]
Optimal Vegetation Index	VOPT	$\left(1+0.45cdot\frac{R_{800}^{2}+1}{R_{670}+0.45}\right)$	[36]
Transformed Chlorophyll Absorption Reflectance Index	TCARI	$3(R_{700}-R_{670})-0.2(R_{700}-R_{550}cdot\frac{R_{700}}{R_{670}})$	[29]
Soil Adjusted Vegetation Index	SAVI	$1.5\cdot \frac{R_{800}-R_{670}}{R_{800}+R_{670}+0.5}$	[31]
Simple Ratio 800−500	SR800550	$R_{800}/R_{550}$	[30]
Chlorohpyll Index Red Edge	CIIRE	$R_{750}/R_{710}-1$	[30]
Leaf Chlorophyll Index	LCI	$\frac{R_{850}-R_{710}}{R_{850}+R_{680}}$	[30]
Anthocyanin Reflectance Index	ARI	$1/R_{550}-1/R_{700}$	[30]
Simple Pigment Ratio Index	SPRI2	$R_{750}/R_{556}$	[30]
Blue Red Pigment Index	BRI	$R_{400}/R_{690}$	[30]
SPAD Index	SPADI	$R_{650}/R_{940}$	[30]
Gitelson and Merzylak Index 1	GM1	$R_{750}/R_{550}$	[30]
Ration Vegetation Index 1	RVI1	$R_{750}/R_{705}$	[30]
Carontenoid Reflectance Index	CRI1	$1/R_{510}-1/R_{550}$	[30]
Red-Edge Vegetation Stress Index	RVSI	$0.5(R_{772}+R_{763})-R_{753}$	[30]
Normalized Difference Vegetation Index	NDVI	$\frac{R_{750}-R_{680}}{R_{750}+R_{680}}$	[30]
Normalized Difference Vegetation Index	NDVI3	$\frac{R_{780}-R_{715}}{R_{780}+R_{715}}$	[30]
Normalized Difference Vegetation Index	NDVI6	$\frac{R_{800}-R_{700}}{R_{800}+R_{700}}$	[30]
Pigment Specific Normalized Difference B	PSNDB	$\frac{R_{800}-R_{680}}{R_{800}+R_{680}}$	[30]
Lichtenhaler Index 2	LIC2	$\frac{R_{790}-R_{680}}{R_{790}+R_{680}}$	[30]

### Downsampling methodology

The precision of the ASD Terraspec enables the reconstruction of the spectral signature of the samples at a resolution of 1 nm, producing 2151 reflectance values ranging from 350 nm to 2500 nm.

For this study, we aim to identify the minimum number of wavelengths that allow the reconstruction of some of the most important vegetation indices used for the assessment of vegetation health status. To achieve this, we progressively reduce the number of wavelengths in the spectral signature of the samples. We begin by selecting wavelengths starting from 350 nm and then every 5 nm, resulting in a total of 431 wavelengths.

This process is replicated by selecting wavelengths at intervals of 10, 20, 30, 40, 50, 75, and 100 nm. Table 3 contains information on the sampling period, the resulting number of wavelengths obtained through the down-sampling process, and the percentage of information used at each sampling period.

**Table 3:** Downsampling periods and the number of wavelengths.

Sampling wavelength	Total Wavelengths	Percentage of Information
1 (original)	2151	100%
5	431	20%
10	216	10%
20	108	5%
30	72	3.35%
40	54	2.51%
50	44	2.05%
75	29	1.35%
100	21	1.02%

**Fig 4:**
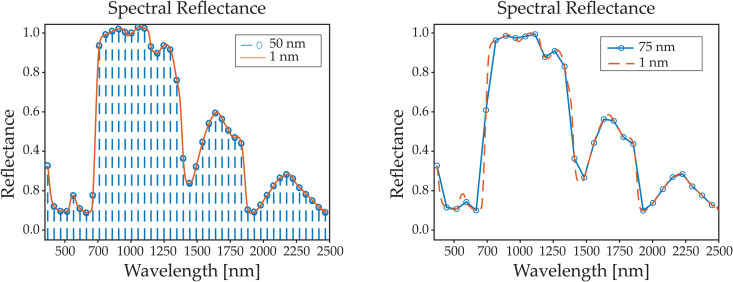
Down sampling of the spectral signature. Shows the sampling interval at 50 nm, considerably reducing the number of points from the original points (left). Shows a linear interpolation of the spectral wavelengths sampled every 75 nm, the linear interpolation is not able to capture all the features of the spectral signature (right).

Fig 4 illustrates sampling every 50 nm compared to the full spectral information, demonstrating a significant reduction in the total number of data points. As expected, performing a simple linear interpolation with the down-sampled information results in a significant loss of information compared to the complete spectral data. This effect can be observed in Fig 4, where the reconstructed reflectance at intervals of 75 nm using linear interpolation loses some of the shapes present in the full spectral data.

Fig 4 shows sampling every 50 nm compared to the full spectral information, it can be seen that the total number of points is reduced considerably.

### Techniques

The vegetation indices were predicted based on the spectral reflectance of the olive leaves at different dehydration stages using WEKA software [37–39]. A procedure was applied to reconstruct the different vegetation indices from the leaf reflectance and to determine the relationships between vegetation indices and spectral reflectance in the full spectrum (350 nm to 2500 nm) with 5, 10, 20, 30, 40, 50, 75, 100 nm down-sampling.

A total of 111 leaves were analyzed at each stage. First, the reflectance in the VNIR region was characterized. Then, a Correlation-based Feature Selection (CFS) subset evaluator and Best First algorithm were applied to the dataset to identify attributes with a higher impact. Input features for 25 different vegetation indices, 9 different down-sampling methods, and different dehydration stages were determined by the CFS attribute selection technique. Predictions were carried out using 4 different machine learning algorithms: random forest (RF), multilayer perceptron (MLP), support vector regression (SVR), and k-nearest neighbor (k-NN).

A k-fold cross-validation method was used to test each model. The k-fold value was set to 10 in this study. This method divided the dataset into 10 equal sections at a ratio of 9:1 for the training and test sets. The learning procedure was carried out, with nine parts serving as training sets and one new part serving as the test set [40].

The Pearson VII (PUK) kernel function was selected in the SVR algorithm. In MLP, the number of neurons in the hidden layer was twice the number of inputs in each model. The sigmoid activation function was used, the number of epochs was 500, the momentum coefficient was 0.2, and the learning rate was 0.3. In k-NN, a k value of 5 was chosen, and the Euclidean distance rule was used in the search process. Approximately 236610 spectrum and 2750 vegetation index data points were utilized for each stage (a total of 1419660 and 16500 data points, respectively). These values represent quantities used only in the 1 nm range. In addition, these values were reduced for all down-sampling methods.

Model performance was assessed using the following statistical metrics: coefficient of determination (R^2^) and root mean square error (RMSE). The statistical performance results were analyzed to evaluate the success of the predictions following the principles specified by [41]. While R measures the strength of the linear relationship between predicted and reference vegetation indices, RMSE and MAE quantifies prediction accuracy in absolute terms by capturing both systematic bias and random error. Therefore, the combined use of R and RMSE provides a more comprehensive assessment of model predictive performance.

## Results

This section presents the results of the techniques applied to the data for predicting vegetation indices (VIs) from down-sampled information. Both the feature selection algorithm and the machine learning methods are analyzed.

### Feature selection

**Fig 5:**
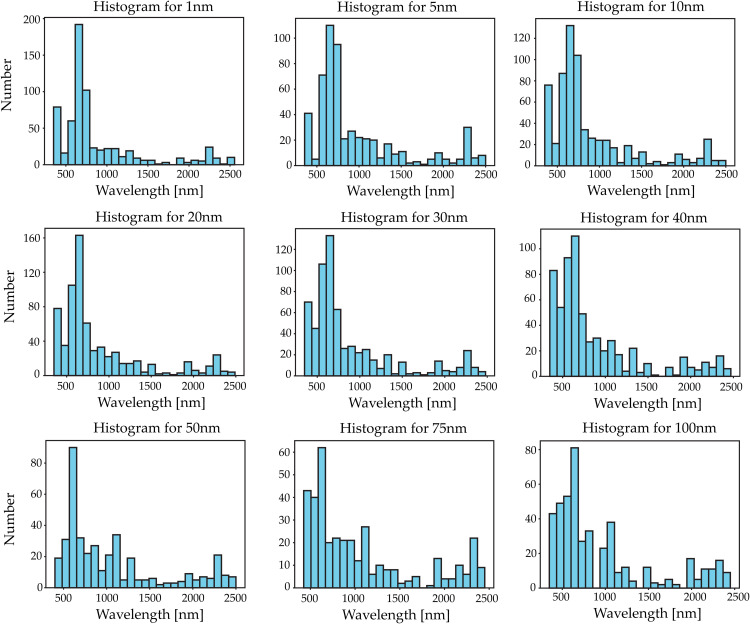
Histograms for each sampling period. Starting from the top left corner: Histogram at 1nm, 5nm, 10nm, 20nm, 30nm, 40nm, 50nm, 75nm, and 100nm at the bottom right corner.

Depending on the vegetation index, dehydration stage, and sampling wavelength, different bands of the spectrum were selected by the algorithm due to their stronger correlation with the target vegetation index. Fig 5 shows the histograms for each sampling wavelength, combining features from the different dehydration stages to create the histogram.

It is important to note that most of the wavelengths selected by the algorithm fall within the range of 500–1,000 nm, regardless of the sampling interval.

**Fig 6:**
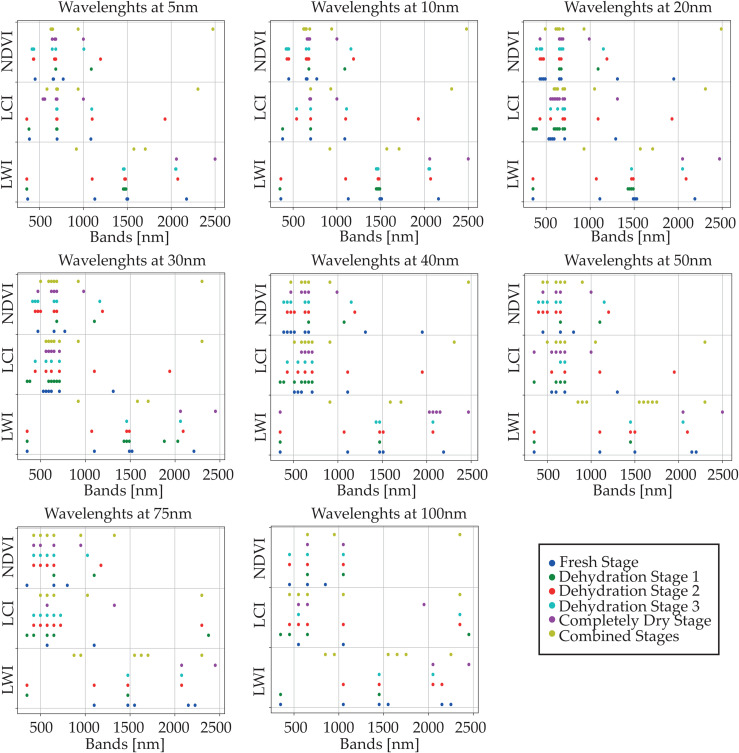
Bands used as input features chosen by CFS for predicting LWI, LCI, and NDVI indices. From the top left are the bands chosen at 5nm and the center bottom are the bands chosen for 100 nm as sampling wavelength.

For a more precise visualization of the bands at different sampling wavelengths and dehydration stages, Fig 6 illustrates the various bands used as features for predicting three vegetation indices: LWI, LCI, and NDVI. The bands are depicted at different dehydration stages and sampling wavelengths.

As shown in the figure, the algorithm selects bands within similar ranges regardless of the dehydration stage and sampling wavelength.

### Machine learning algorithms

**Fig 7:**
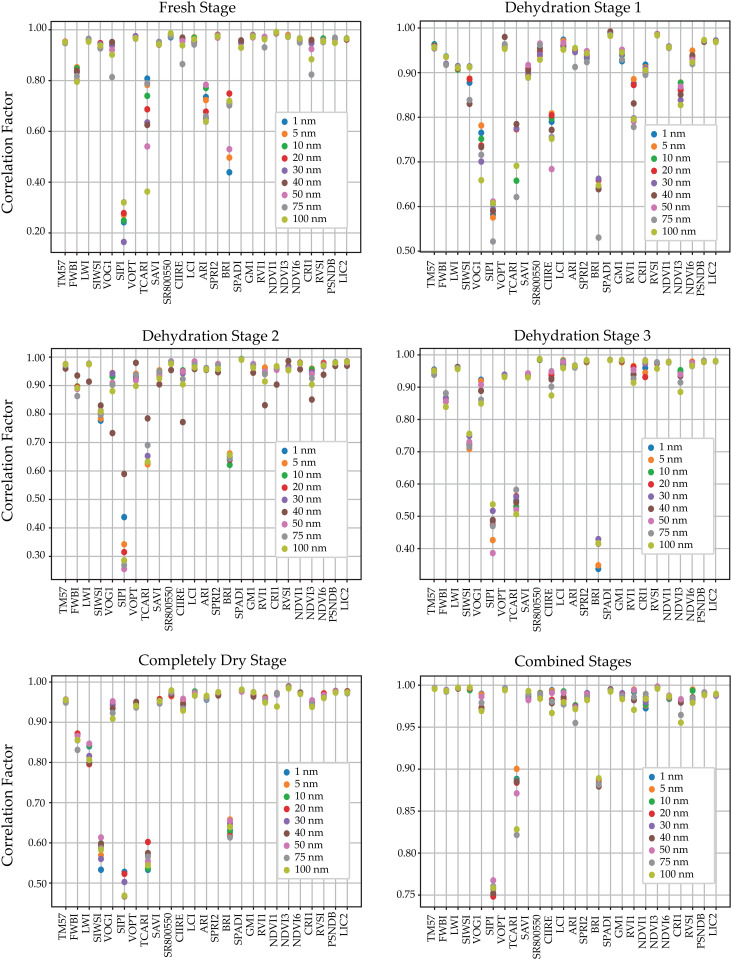
Coefficient of determination obtained from the different machine learning models for each vegetation index. The top left corner shows the fresh stage, followed by the first, second, and third dehydration stages, the fully dried stage, and the combined stages at the bottom left corner.

**Fig 8:**
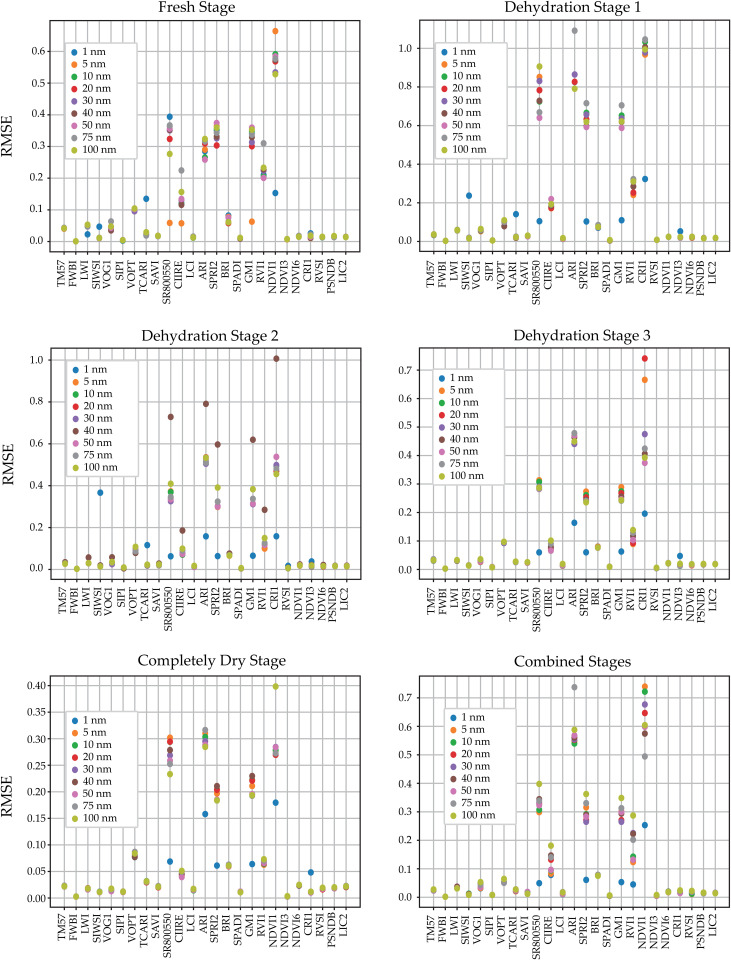
RMSE obtained from the different machine learning models for each vegetation index. The top left corner shows the fresh stage, followed by the first, second, and third dehydration stages, the fully dried dehydration stage, and the combined stages at the bottom left corner.

As stated in previous sections the performance of the ML algorithms was evaluated using the metrics of coefficient of determination and RMSE. Four ML models were used to predict twenty-five vegetation indices. Spectral features important for estimating VIs were selected using CFS feature selection for the complete data and metrics. The analysis showed that the four proposed algorithms produced similar results, thus we averaged the values to represent better and visualize the metrics obtained by the tested algorithms, the averaged coefficient of determination and RMSE values per down-sampled data are shown in Figs 7 and 8 respectively.

The k-fold cross-validation method was used to determine the performance of the ML models. Herein, the k value was chosen as 10, which means that the training/test dataset was divided into 10 parts. This method was repeated 10 times (with the same number of k values) using each subset as the test data. Finally, the model performance corresponds to the average of the k-pieces training. However, the effect of spectral reflectance values in different ranges on the prediction results was investigated using the downsampling method. It is important to note that each VI and downsampling implementation is defined within a different range. This can be understood by observing the scatter of the coefficient of determination in each row of the averages of the ML models presented in Fig 7.

As the figures show, in most cases, the reconstruction of the VIs is successful even with the down-sampling information at 100 nm. Most notably, the SIPI index presents the lowest coefficient of determination at every dehydration stage, reaching values as low as 0.15 in the fresh stage. Other indices with this trend are BRI and TCARI. However, when training the models with the information of the combined stages, all the models achieve coefficients of determination over 0.75, this can be explained by the fact that the models were trained with a larger dataset, thus producing more robust results.

The NDVI and SR800550 indices gave the highest coefficient of determination values for the fresh stage, while the SIPI index gave the lowest. SPADI and RVSI in dehydration stage 1, SPADI in dehydration stage 2, SR800550 in dehydration stage 3, and NDVI3 in the completely dry stage were the indices with higher success rates. However, the coefficient of determination results of the SIPI were much lower than those of the other indices.

In general, the performance results of the regressors were similar. In particular, the coefficient of determination values showed that the RF algorithm is more successful in predicting twenty-five different VIs. It was found that 5 and 10 nm down-sampled information had higher model success than the others, which is expected since most of the information was complete for predicting the vegetation indices.

As expected, the performance was lower for the 75 and 100 nm down-sampling methods. This is thought to be due to the loss of spectral reflectance values in between. Among the dehydration stages, the combined stages had the highest average coefficient of determination (0.9613), whereas dehydration stage 1 (0.8742) had the lowest average. This can be explained by the fact that more data were used during the training phase, therefore increasing the robustness of the models.

Comparing the performance of regressors with multiple metrics allows validation of the model results. The RMSE value helps to compare the performance of an index retrieval between the training and test sets. In this respect, the RMSE of the training and test sets were comparable, indicating that the models were not overfitting. The RMSE results for the averages of the four ML models are shown in Fig 8. The indices with the lowest RMSE values for the fresh stage, dehydration stage 1, dehydration stage 2, dehydration stage 3, fully dry stage, and combined stages were defined as the FWBI.

In addition, the RF model generally had the lowest RMSE values, followed by the MLP model. These values show that the RF regressor is better than the other ML models for predicting the vegetation indices. However, the RMSE results of the 75 and 100 nm down-sampling methods were high. The average RMSE values for all applications varied between 0.0780 (completely dry stage) and 0.2118 (dehydration stage 1).

Across all spectral resolutions ranging from 1 to 100 nm, the predictive performance of vegetation indices exhibited a gradual and resolution-dependent variation. Mean Absolute Error (MAE) values generally increased with decreasing spectral resolution; however, this increase remained limited for several widely used indices. In particular, NDVI-based indices, SAVI, LCI, SPADI, and RVSI consistently showed low MAE values even at coarser resolutions, indicating strong robustness to spectral down-sampling. Among the evaluated machine learning models, RF and MLP demonstrated the most stable and accurate performance across all resolutions, whereas SVR and kNN showed higher sensitivity to reduced spectral detail, especially for pigment-related indices such as ARI, CRI1, and GM1. Notably, acceptable prediction accuracy was preserved up to 100 nm spectral resolution for several indices, highlighting the potential of reduced-resolution spectral data for reliable vegetation index estimation.

For tables with the complete metrics for all the models, please see the supplementary material.

## Discussion

This study evaluated the potential of predicting vegetation indices (VIs) obtained from spectral reflections using machine learning (ML) models at different dehydration stages and down-sampling methods. Predicting VIs at different dehydration stages of the olive leaves based on spectral reflectance may yield varying performance results due to differences in crop types, dehydration conditions, and feature selection methods. In this study, selecting appropriate ML model parameters and input features, along with combining data from different treatments, increased the accuracy of the models. This integrative approach is not only novel in its design but also scalable and adaptable, enabling the reuse of the same framework for different crops, stress conditions, and acquisition setups. The framework’s flexibility demonstrates its applicability beyond a specific crop or condition.

The VNIR region concentrates the most pronounced changes in the measurements. A loss in the leaf’s moisture content can alter the reflection intensity. For olive leaves, the effects of the dehydration stages are evident in the VNIR region. In addition, each species has a specific shape and intensity of the spectral signature in the VNIR region. Based on the recorded reflectance, the dataset was constructed using the reflectance of each sample in the VNIR region. These changes can be observed in the histograms shown in Fig 5. The overlap of the average reflectance for olive leaves at different dehydration stages and as a result of different downsampling treatments supports the attributes chosen for the estimation. Similar approaches were reported by [26] in the *Eucalyptus globulus* species. However, few studies have systematically explored how spectral degradation (via down-sampling) affects the prediction of multiple vegetation indices under various dehydration stages—this study fills that gap.

At different dehydration stages and sampling wavelengths, bands within similar ranges were selected and used as features to predict different VIs. Figs 5 and 6 show that most of the bands selected by the algorithm fall within the VNIR region. This suggests a strong relationship between the VNIR SWIR regions, which can be utilized to characterize the foliage more precisely. The ability to identify this relationship through feature selection provides a theoretical foundation for compressing hyperspectral data into compact, informative subsets, potentially guiding the design of next-generation multi-spectral sensors.

By investigating this relationship in depth, new types of sensors could be developed that allow for the recording of similar electromagnetic spectra in this range to extract vegetation characteristics adequately. The proposed approach can also be used in combination with lower-cost sensors that specifically record information in the VNIR region to predict the full reflectance spectrum of leaves. This suggests an opportunity for cost-efficient remote sensing systems that retain hyperspectral-level intelligence while utilizing minimal hardware complexity. By reconstructing the spectrum, many vegetation indices can be calculated, enabling moisture estimation and other assessments. These results offer strong support for building lightweight, application-specific monitoring systems that leverage a reduced number of spectral bands while retaining high predictive accuracy.

The results clearly demonstrate that high predictive accuracy for several vegetation indices can be maintained even under substantially reduced spectral resolution, particularly for indices based on broad spectral features such as NDVI, SAVI, and water-related indices such as FWBI, LWI and SIWSI. The high performance of our approach in these indices can be attributed to the downsampling technique, which maintains key regions related to water absorption. This behavior can also be explained to the capabilities of the models to capture the relationships among the different regions of the spectrum, even in the absence of complete spectral information. In contrast, pigment-sensitive indices such as ARI, CRI1, and GM1 exhibited a marked increase in MAE with decreasing resolution, reflecting their dependence on narrow spectral features and higher susceptibility to information loss during down-sampling. The consistently strong performance of RF and MLP across all resolutions suggests that these models are better suited to capture nonlinear relationships and maintain robustness despite the lack of complete spectral information. Importantly, the preservation of acceptable accuracy levels up to 100 nm resolution provides strong evidence that low-cost multispectral or simplified hyperspectral sensing systems may be sufficient for practical vegetation health monitoring applications, particularly under controlled conditions; nevertheless, further validation on field-conditions is needed to corroborate the VI prediction using the downsampling of 100 nm, before starting application of real monitoring of crops. These findings have direct implications for sensor design, indicating that careful band selection may be more critical than high spectral resolution alone.

The coefficient of determination and RMSE values provide important information about the predictions of vegetation indices (VIs), especially in the non-downsampling (1 nm) and combined stages. The RVSI, VOPT, and SPADI indices achieved the highest coefficient of determination values of 0.9986, 0.9963, and 0.9959, respectively. However, in the combined stage with the highest downsampling wavelength (100 nm), the *R*^2^ values of RVSI, VOPT, and SPADI indices decreased slightly to 0.9955, 0.9936, and 0.9921. In addition, the coefficient of determination value for downsampling between 1 nm and 100 nm in the combined stage decreased from 0.9608 to 0.9552, which is still an acceptable level. A similar trend was observed in the non-downsampling (1 nm) and fresh stage, where the RVSI and VOPT indices achieved the highest coefficients of determination of 0.9863 and 0.9826, respectively. These findings confirm that acceptable levels of accuracy are maintained even under high spectral compression, which reinforces the feasibility of real-time field deployment using simplified spectral systems. These findings confirm that acceptable levels of accuracy are maintained even under high spectral compression, which enhances the feasibility of real-time field deployment using simplified spectral systems.

In general, the coefficient of determination values of the RF regressor are higher, indicating that the RF model is suitable for predicting the twenty-five VIs. All regressors were able to predict leaf reflectance values. In particular, the average coefficient of determination values for all downsampling treatments and models were higher than 0.8742, and the highest were obtained by the RF algorithm for the RVSI, TM57, and LWI indices, suggesting that RF is more effective at predicting VIs than other alternatives. RMSE values support the effectiveness of the proposed method; in all cases, RMSE values were lower than 0.2118. This consistently strong performance endorses the choice of ensemble-based regressors, especially when generalizability and model robustness are critical.

The high coefficients of determination observed in several vegetation indices can be attributed to the controlled experimental conditions and the highly correlated spectral bands within the same region of the spectrum. All spectral measurements were acquired using a high-resolution spectrometer under laboratory conditions, which significantly reduced sensor noise, illumination variability, and atmospheric effects commonly present in field-based or UAV-based measurements. Furthermore, the dataset exhibits a high degree of homogeneity, as all samples belong to the same species and were subjected to identical dehydration protocols. In these situations, changes in spectral reflectance are largely influenced by the changes in leaf water status due to the dehydration process, rather than driven by biological or environmental variations. Mathematical combinations of reflectance values at specific wavelengths form the basis of vegetation indices. We expect strong linear relationships between the predicted and reference indices when these wavelengths or spectrally correlated neighboring bands are available as model inputs. Correlation-based feature selection reinforces this effect by prioritizing the most informative spectral bands and minimizing redundancy. Comparable high coefficients of determination values have been reported in previous laboratory-based studies involving vegetation index reconstruction and moisture-related spectral analysis [26,27], supporting the validity of the results obtained in this work.

Important wavelengths for the prediction of vegetation indices were identified, particularly between *R*_600_ and *R*_750_ in the VNIR region. The study indicated that two or three wavelengths may be sufficient for the prediction of some vegetation indices, which may provide information about the different biophysical properties and health status of the plant. In addition, the proposed method can improve the output data from multi-spectral cameras. Such a minimal-band approach offers the potential for on-chip implementation in portable devices and drones, supporting high-throughput phenotyping and on-site decision-making.

The models created by training the algorithms separately for each dehydration stage performed worse compared to models trained for the combined stage. However, the performance results of the algorithms for other dehydration stages were similar due to dehydration under the same conditions (convective drying). Despite this limitation, it is evident that specific models, such as random forest (RF) and multilayer perceptron (MLP), achieve lower RMSE values than other models. Together, these insights validate the practicality and novelty of the proposed approach, which minimizes spectral requirements while maintaining robust VIs prediction performance. Overall, this study delivers a proof-of-concept for compressive yet accurate vegetation analysis, balancing spectral resolution, cost, and model generalization—a step forward in accessible remote sensing.

## Conclusion

This study presents the prediction of vegetation indices from down-sampled spectral signatures using correlation feature selection and machine learning algorithms. The approach is tested on a dataset composed of spectral signatures from olive leaves. Our work demonstrates that machine learning algorithms can predict vegetation indices using spectral signatures with a 100 nm spectral resolution, achieving a coefficient of determination of over 0.9. However, as expected, reconstruction with higher spectral resolutions of 5 nm and 10 nm produces better results. This confirms the feasibility of vegetation index estimation with reduced spectral data, enabling practical applications under hardware-limited conditions.

Correlation feature selection was used to select the bands for predicting each vegetation index at each dehydration stage and with each subsampled dataset. As shown in Fig 5, most of the selected bands are located within the range of 500–1000 nm, which supports the findings of [26] on reconstructing the SWIR region of the spectrum using the VNIR region. Our results reinforce the idea that meaningful spectral features are highly concentrated in the VNIR region, making it a suitable target for sensor optimization.

The selection of the machine learning algorithm was not highly critical, as only slight differences in performance were observed among the four algorithms tested. However, the RF algorithm yielded the best performance. Importantly, the proposed method does not rely on full-spectrum data acquisition but instead leverages a combination of spectral down-sampling, intelligent band selection, and model-based prediction, forming an efficient and adaptable framework for VIs estimation.

Unlike many previous approaches that focus on individual indices or require high-resolution input, our framework enables the prediction of multiple vegetation indices across diverse physiological stages, while maintaining high accuracy with minimal spectral input. Our study suggests using sensors with lower spectral resolution for health monitoring of vegetation, rather than spectrometers or hyperspectral cameras with spectral resolutions of 1 nm, such as the ASD spectrometer used in this study. Future work involves the validation of our approach on field-conditions rather than a controlled lab environment, for fine tunning of the models. Furthermore, this approach validated of field-conditions can be extended for real-time monitoring of crops using robotic platforms such as UAVs or other handheld devices, offering scalable solutions for precision agriculture.

## Supporting information

S1 File Prediction of vegetation indices from down-sampled hyperspectral data using machine learning: A novel framework for olive crop monitoring. **Data Tables**: This file contains all the metrics obtained by the machine learning algorithms in the prediction of the 24 different VIs, using as input the downsampled information at 1, 5, 10, 20, 30, 40, 50, 75 and 100 nm.
(PDF)

S2 File Inclusivity in global research questionnaire. (PDF)

## References

[1] Ruiz-Carrasco B, Fernández-Lobato L, López-Sánchez Y, Vera D. Life cycle assessment of olive oil production in turkey, a territory with an intensive production project. Agriculture. 2023;13(6):1192.

[2] Office of Agricultural Studies and Policies. Chilean agriculture overview. 2019.

[3] Vougioukas SG. Agricultural Robotics. Annu Rev Control Robot Auton Syst. 2019;2(1):365–92. doi: 10.1146/annurev-control-053018-023617

[4] Estrada JS, Fuentes A, Reszka P, Auat Cheein F. Machine learning assisted remote forestry health assessment: a comprehensive state of the art review. Front Plant Sci. 2023;14:1139232. doi: 10.3389/fpls.2023.1139232 37332724 PMC10272373

[5] Lu J, Cheng D, Geng C, Zhang Z, Xiang Y, Hu T. Combining plant height, canopy coverage and vegetation index from UAV-based RGB images to estimate leaf nitrogen concentration of summer maize. Biosystems Eng. 2021;202:42–54. doi: 10.1016/j.biosystemseng.2020.11.010

[6] Eugenio FC, Grohs M, Schuh M, Venancio LP, Schons C, Badin TL, et al. Estimated flooded rice grain yield and nitrogen content in leaves based on RPAS images and machine learning. Field Crops Res. 2023;292:108823.

[7] Brinkhoff J, Dunn BW, Robson AJ. Rice nitrogen status detection using commercial-scale imagery. Int J Appl Earth Observ Geoinform. 2021;105:102627. doi: 10.1016/j.jag.2021.102627

[8] Marang IJ, Filippi P, Weaver TB, Evans BJ, Whelan BM, Bishop TFA, et al. Machine Learning Optimised Hyperspectral Remote Sensing Retrieves Cotton Nitrogen Status. Remote Sens. 2021;13(8):1428. doi: 10.3390/rs13081428

[9] Shanmugapriya P, Latha KR, Pazhanivelan S, Kumaraperumal R, Karthikeyan G, Sudarmanian NS. Cotton yield prediction using drone derived LAI and chlorophyll content. J Agrometeorol. 2022;24(4):348–52. doi: 10.54386/jam.v24i4.1770

[10] Messina G, Praticò S, Badagliacca G, Di Fazio S, Monti M, Modica G. Monitoring Onion Crop “Cipolla Rossa di Tropea Calabria IGP” Growth and Yield Response to Varying Nitrogen Fertilizer Application Rates Using UAV Imagery. Drones. 2021;5(3):61. doi: 10.3390/drones5030061

[11] Peng J, Manevski K, Kørup K, Larsen R, Andersen MN. Random forest regression results in accurate assessment of potato nitrogen status based on multispectral data from different platforms and the critical concentration approach. Field Crops Res. 2021;268:108158. doi: 10.1016/j.fcr.2021.108158

[12] Liu J, Zhu Y, Tao X, Chen X, Li X. Rapid prediction of winter wheat yield and nitrogen use efficiency using consumer-grade unmanned aerial vehicles multispectral imagery. Front Plant Sci. 2022;13:1032170. doi: 10.3389/fpls.2022.1032170 36352879 PMC9638066

[13] Amarasingam N, Gonzalez F, Arachchige S, Salgadoe A, Unupen WLM, Hettiarachchige AS, et al. Predicting canopy chlorophyll content in sugarcane crops using machine learning algorithms and spectral vegetation indices derived from uav multispectral imagery. Remote Sens. 2022;14(5):1140.

[14] Simic Milas A, Romanko M, Reil P, Abeysinghe T, Marambe A. The importance of leaf area index in mapping chlorophyll content of corn under different agricultural treatments using UAV images. Int J Remote Sens. 2018;39(15–16):5415–31. doi: 10.1080/01431161.2018.1455244

[15] Longfei Z, Ran M, Xing Y, Yigui L, Zehua H, Zhengang L, et al. Improved Yield Prediction of Ratoon Rice Using Unmanned Aerial Vehicle-Based Multi-Temporal Feature Method. Rice Sci. 2023;30(3):247–56. doi: 10.1016/j.rsci.2023.03.008

[16] Teodoro PE, Teodoro LPR, Baio FHR, da Silva Junior CA, dos Santos RG, Marques Ramos AP, et al. Predicting days to maturity, plant height, and grain yield in soybean: A machine and deep learning approach using multispectral data. Remote Sens. 2021;13(22):4632.

[17] Tedesco D, Freire de Oliveira M, dos Santos AF, Costa Silva EH, Rolim G de S, Pereira da Silva R. Use of remote sensing to characterize the phenological development and to predict sweet potato yield in two growing seasons. Eur J Agron. 2021;129:126337.

[18] dos Santos LM, Ferraz GA e S, Marin DB, Carvalho MA de F, Dias JEL, Alecrim A de O, et al. Vegetation Indices Applied to Suborbital Multispectral Images of Healthy Coffee and Coffee Infested with Coffee Leaf Miner. AgriEngineering. 2022;4(1):311–9. doi: 10.3390/agriengineering4010021

[19] Santos LB, Bastos LM, de Oliveira MF, Soares PLM, Ciampitti IA, da Silva RP. Identifying Nematode Damage on Soybean through Remote Sensing and Machine Learning Techniques. Agronomy. 2022;12(10):2404. doi: 10.3390/agronomy12102404

[20] Simões IOPS, do Amaral LR. UAV-based multispectral data for sugarcane resistance phenotyping of orange and brown rust. Smart Agric Technol. 2023;4:100144.

[21] Cao X, Du H, Tong X, Dai Q, Lin S. A Prism-Mask System for Multispectral Video Acquisition. IEEE Trans Pattern Anal Mach Intell. 2011;33(12):2423–35. doi: 10.1109/TPAMI.2011.80 21519097

[22] Zhao J, Kumar A, Banoth BN, Marathi B, Rajalakshmi P, Rewald B, et al. Deep-Learning-Based Multispectral Image Reconstruction from Single Natural Color RGB Image—Enhancing UAV-Based Phenotyping. Remote Sens. 2022;14(5):1272. doi: 10.3390/rs14051272

[23] Shi Z, Chen C, Xiong Z, Liu D, Wu F. HSCNN+: Advanced CNN-Based Hyperspectral Recovery from RGB Images. In: 2018 IEEE/CVF Conference on Computer Vision and Pattern Recognition Workshops (CVPRW), 2018. 1052–8. 10.1109/cvprw.2018.00139

[24] Zhang Y, Yang W, Zhang W, Yu J, Zhang J, Yang Y, et al. Two-step ResUp&Down generative adversarial network to reconstruct multispectral image from aerial RGB image. Comput Electron Agric. 2022;192:106617. doi: 10.1016/j.compag.2021.106617

[25] Gkillas A, Kosmopoulos D, Berberidis K. Cost-efficient coupled learning methods for recovering near-infrared information from RGB signals: Application in precision agriculture. Comput Electron Agric. 2023;209:107833. doi: 10.1016/j.compag.2023.107833

[26] Arevalo-Ramirez T, Villacrés J, Fuentes A, Reszka P, Auat Cheein FA. Moisture content estimation of Pinus radiata and Eucalyptus globulus from reconstructed leaf reflectance in the SWIR region. Biosyst Eng. 2020;193:187–205. doi: 10.1016/j.biosystemseng.2020.03.004

[27] Villacrés J, Arevalo-Ramirez T, Fuentes A, Reszka P, Auat Cheein F. Foliar Moisture Content from the Spectral Signature for Wildfire Risk Assessments in Valparaíso-Chile. Sensors (Basel). 2019;19(24):5475. doi: 10.3390/s19245475 31842283 PMC6960617

[28] Villacrés J, Fuentes A, Reszka P, Cheein FA. Retrieval of Vegetation Indices Related to Leaf Water Content from a Single Index: A Case Study of Eucalyptus globulus (Labill.) and Pinus radiata (D. Don.). Plants (Basel). 2021;10(4):697. doi: 10.3390/plants10040697 33916338 PMC8067298

[29] Yang H, Ming B, Nie C, Xue B, Xin J, Lu X, et al. Maize Canopy and Leaf Chlorophyll Content Assessment from Leaf Spectral Reflectance: Estimation and Uncertainty Analysis across Growth Stages and Vertical Distribution. Remote Sens. 2022;14(9):2115. doi: 10.3390/rs14092115

[30] El-Hendawy S, Dewir YH, Elsayed S, Schmidhalter U, Al-Gaadi K, Tola E, et al. Combining Hyperspectral Reflectance Indices and Multivariate Analysis to Estimate Different Units of Chlorophyll Content of Spring Wheat under Salinity Conditions. Plants (Basel). 2022;11(3):456. doi: 10.3390/plants11030456 35161437 PMC8839343

[31] Huete AR. A soil-adjusted vegetation index (SAVI). Remote Sens Environ. 1988;25(3):295–309. doi: 10.1016/0034-4257(88)90106-x

[32] Xue J, Su B. Significant Remote Sensing Vegetation Indices: A Review of Developments and Applications. J Sens. 2017;2017:1–17. doi: 10.1155/2017/1353691

[33] Radočaj D, Šiljeg A, Marinović R, Jurišić M. State of Major Vegetation Indices in Precision Agriculture Studies Indexed in Web of Science: A Review. Agriculture. 2023;13(3):707. doi: 10.3390/agriculture13030707

[34] Govender M, Govender P, Weiersbye I, Witkowski E, Ahmed F. Review of commonly used remote sensing and ground-based technologies to measure plant water stress. Water SA. 2009;35(5). doi: 10.4314/wsa.v35i5.49201

[35] Rubo S, Zinkernagel J. Exploring hyperspectral reflectance indices for the estimation of water and nitrogen status of spinach. Biosyst Eng. 2022;214:58–71. doi: 10.1016/j.biosystemseng.2021.12.008

[36] Yang R, Kan J. Classification of Tree Species in Different Seasons and Regions Based on Leaf Hyperspectral Images. Remote Sens. 2022;14(6):1524. doi: 10.3390/rs14061524

[37] Bouckaert RR, Frank E, Hall M, Kirkby R, Reutemann P, Seewald A, et al. Weka manual for version 3-9-1. Hamilton, New Zealand: University of Waikato; 2016. p. 1–341.

[38] Frank E, Hall MA, Witten IH. The WEKA workbench. Morgan Kaufmann; 2016.

[39] Witten IH. Data mining: practical machine learning tools and techniques. 2nd edition. Morgan Kaufmann; 2005.

[40] Ropelewska E, Sabanci K, Aslan MF, Çetin N. Rapid Detection of Changes in Image Textures of Carrots Caused by Freeze-Drying using Image Processing Techniques and Machine Learning Algorithms. Sustainability. 2023;15(8):7011. doi: 10.3390/su15087011

[41] Colton T. Statistics in medicine little. Boston: Brown and Company; 1974. p. 164–8.

