# Supplementary material for: Prediction of vegetation indices from down-sampled hyperspectral data using machine learning: A novel framework for olive crop monitoring
Source: PLoS One. 2026 Mar 27;21(3):e0323158. doi: 10.1371/journal.pone.0323158 (PMC13029797; doi:10.1371/journal.pone.0323158)
Supplement: S1 File — Data Tables: This file contains all the metrics obtained by the machine learning algorithms in the prediction of the 24 different VIs, using as input the downsampled information at 1, 5, 10, 20, 30, 40, 50, 75 and 100 nm. (PDF) [file pone.0323158.s001.pdf]

Prediction of vegetation indices from down-sampled hyperspectral data using machine learning: A novel framework for olive crop monitoring

Juan Sebastián Estrada[1], Necati Cetin[2], Kamil Sacilik[2], Zhen Guo[3,4], Fernando Auat Cheein[4,5,*]

[1] Department of Electronic Engineering, Universidad Tecnica Federico Santa Maria, Av. España 1680, Valparaíso, Chile

[2] Faculty of Agriculture, Department of Agricultural Machinery and Technologies Engineering, Ankara University, Ankara, Turkey

[3] School of Agricultural Engineering and Food Science, Shandong University of Technology, Zhangdian District, Zibo, Shandong 255000, China

[4] Department of Agricultural Engineering, Harper Adams University, Newport, Shropshire TF10 8NB, UK

[5] Heriot-Watt University, Scotland, UK

[*] Corresponding author: FAuat@harper-adams.ac.uk, f.auat@hw.ac.uk

1 Suplementary Materials: Data tables

This appendix includes data tables detailing the input bands selected by the CFS algorithm and the evaluation metrics. As stated in the text, sampled data at resolutions of 1, 5, 10, 20, 30, 40, 50, 75, and 100 nm was used to predict 25 vegetation indices (VIs) using four machine learning algorithms. These algorithms were tested across five different dehydration stages as well as a combination of stages. This analysis resulted in 54 tables, which are presented below.

VIs	Inputs	R^2				$RMSE$			
		RF	MLP	SVR	kNN	RF	MLP	SVR	kNN
TM57	352, 2311, 2327, 2328, 2346, 2364, 2372	0.9635	0.9515	0.9524	0.9669	0.0332	0.0393	0.0375	0.0325
FWBI	350, 748	0.9361	0.9201	0.9482	0.9230	0.0030	0.0034	0.0027	0.0034
LWI	352, 1462, 1468, 1473, 1474, 1475	0.9089	0.8925	0.9287	0.9341	0.0588	0.0720	0.0523	0.0508
SIWSI	765, 898, 1886, 1890, 1891	0.8775	0.9360	0.9394	0.8870	0.2372	0.1298	0.1445	0.0167
VOG1	366, 699, 701, 2157	0.7653	0.7345	0.7713	0.7551	0.0552	0.0374	0.0347	0.0353
SIPI	679, 682, 685, 1343	0.5818	0.2884	0.4309	0.4922	0.0052	0.0070	0.0056	0.0055
VOPT	686, 687, 688, 832, 896	0.9556	0.9969	0.9916	0.9609	0.1080	0.0279	0.0478	0.1042
TCARI	720	0.6579	0.6761	0.7115	0.6967	0.1413	0.1523	0.1377	0.1340
SAVI	687, 688, 926	0.8965	0.9272	0.9414	0.9039	0.0293	0.0249	0.0224	0.0284
SR800550	379, 557, 564	0.9402	0.9282	0.8940	0.9357	0.1049	0.1242	0.1576	0.1073
CIIRE	366, 697, 698	0.7900	0.7836	0.8148	0.7985	0.1804	0.1899	0.1766	0.1762
LCI	366, 379, 696, 697, 698, 699, 700, 701	0.9737	0.9797	0.9819	0.9722	0.0129	0.0117	0.0108	0.0134
ARI	551, 726	0.9508	0.9468	0.8344	0.9031	0.8257	0.8768	1.3086	1.1429
SPRI2	380, 563, 574, 581	0.9322	0.9429	0.8812	0.9343	0.1035	0.0974	0.1523	0.1021
BRI	688, 1369	0.6411	0.5108	0.6306	0.6447	0.0708	0.0886	0.0719	0.0703
SPADI	367, 649, 687, 688, 1091	0.9891	0.9985	0.9979	0.9887	0.0055	0.0021	0.0025	0.0069
GM1	380, 563, 574	0.9255	0.9360	0.8871	0.9239	0.1099	0.1062	0.1486	0.1095
RVI1	366, 694, 695, 696, 926	0.8756	0.9007	0.8629	0.8519	0.2480	0.2248	0.2747	0.2732
CRI1	380, 388, 415, 845	0.9178	0.9222	0.8129	0.9092	0.3234	0.4827	1.1691	0.3419
RVSI	771, 907	0.9839	0.9874	0.9886	0.9754	0.0085	0.0073	0.0073	0.0072
NDVI1	684, 686, 1088	0.9561	0.9672	0.9771	0.9576	0.0242	0.0210	0.0176	0.0250
NDVI3	361, 702, 703	0.8632	0.8707	0.8905	0.8866	0.0524	0.0515	0.0463	0.0480
NDVI6	352, 693, 1088	0.9378	0.9692	0.9647	0.9285	0.0216	0.0153	0.0165	0.0236
PSNDB	689, 1088	0.9727	0.9777	0.9867	0.9698	0.0165	0.0149	0.0116	0.0188
LIC2	684, 686, 687, 1088	0.9694	0.9787	0.9858	0.9707	0.0183	0.0153	0.0125	0.0193

Results for 1 nm at first dehydration stage

VIs	Inputs	R^2				$RMSE$			
		RF	MLP	SVR	kNN	RF	MLP	SVR	kNN
TM57	1088, 2161, 2164, 2165, 2555, 2259, 2264, 2401, 2480	0.9754	0.9848	0.9825	0.9791	0.0272	0.0214	0.0230	0.0270
FWBI	736, 739, 741, 745, 746, 748, 790, 2243	0.8895	0.9044	0.9117	0.9056	0.0029	0.0029	0.0028	0.0027
LWI	355, 1105, 1476, 1477, 2069	0.9748	0.9919	0.9803	0.9796	0.0305	0.0173	0.0288	0.0289
SIWSI	359, 1026, 1890	0.7769	0.8450	0.8488	0.8033	0.3667	0.2842	0.3333	0.3747
VOG1	706, 707, 708, 709, 1088, 1937	0.9361	0.9474	0.9490	0.9300	0.0265	0.0258	0.0238	0.0281
SIPI	351, 359, 877	0.4379	0.2669	0.4068	0.4165	0.0080	0.0095	0.0082	0.0097
VOPT	680, 681, 885	0.9379	0.9637	0.9722	0.9469	0.0871	0.0686	0.0582	0.0623
TCARI	751	0.6274	0.5984	0.7318	0.5458	0.1161	0.1265	0.1019	0.1314
SAVI	669, 675, 887	0.9529	0.9737	0.9820	0.9560	0.0193	0.0154	0.0120	0.0193
SR800550	557, 558, 560, 565, 1307	0.9783	0.9799	0.9414	0.9739	0.0628	0.0661	0.1407	0.0704
CIIRE	701, 702, 703, 704, 1079	0.9482	0.9583	0.9587	0.9481	0.0742	0.0696	0.0662	0.0755
LCI	355, 696, 698, 700, 702, 703, 1120	0.9818	0.9920	0.9919	0.9830	0.0123	0.0083	0.0082	0.0131
ARI	360, 553, 554, 555, 557, 558, 559, 560, 1307	0.9600	0.9519	0.8755	0.9527	0.1580	0.2491	0.5817	0.1909
SPRI2	559, 564, 565, 589, 1309	0.9754	0.9756	0.9415	0.9648	0.0636	0.0655	0.1201	0.0713
BRI	351, 353, 354, 1324	0.6494	0.5079	0.6440	0.5815	0.0667	0.0781	0.0665	0.0800
SPADI	438, 647, 649, 650, 651, 653, 655, 658, 1284	0.9932	0.9989	0.9981	0.9930	0.0051	0.0021	0.0027	0.0060
GM1	559, 564, 565, 589, 1309	0.9748	0.9762	0.9404	0.9655	0.0655	0.0658	0.1237	0.0725
RVI1	697, 698, 699, 701, 702, 1131	0.9614	0.9623	0.9598	0.9558	0.1021	0.1079	0.1048	0.1107
CRİ1	389, 409, 410, 901	0.9679	0.9393	0.8675	0.9406	0.1583	0.3152	0.9497	0.2151
RVSI	780, 788, 793, 867	0.9678	0.9762	0.9791	0.9645	0.0173	0.0144	0.0131	0.0188
NDVI1	442, 443, 675, 679, 680, 683, 1193	0.9794	0.9846	0.9871	0.9816	0.0188	0.0167	0.0151	0.0186
NDVI3	702, 703, 704, 705, 706, 707, 1088, 2060	0.9534	0.9719	0.9730	0.9404	0.0383	0.0304	0.0296	0.0415
NDVI6	360, 691, 692, 693, 694, 695, 1229	0.9800	0.9862	0.9874	0.9804	0.0131	0.0114	0.0104	0.0143
PSNDB	426, 592, 642, 643, 647, 1193	0.9818	0.9907	0.9906	0.9840	0.0153	0.0112	0.0111	0.0156
LIC2	442, 443, 675, 679, 680, 683, 1193	0.9840	0.9903	0.9915	0.9864	0.0151	0.0120	0.0111	0.0149

Results for 1 nm at second dehydration stage

VIs	Inputs	R^2				$RMSE$			
		RF	MLP	SVR	kNN	RF	MLP	SVR	kNN
TM57	2173, 2255, 2257, 2258, 2259, 2260, 2269, 2338, 2492	0.9544	0.9429	0.9575	0.9502	0.0310	0.0353	0.0301	0.0328
FWBI	744, 752, 755 763, 796, 803, 2235	0.8646	0.8426	0.8835	0.7581	0.0031	0.0035	0.0028	0.0043
LWI	1454, 1461, 1464, 2050, 2052	0.9575	0.9570	0.9617	0.9389	0.0323	0.0340	0.0312	0.0386
SIWSI	372, 1068, 1075, 1885, 1886, 1890	0.7088	0.6750	0.7427	0.6038	0.0147	0.0158	0.0140	0.0175
VOG1	702, 704, 705, 706, 707, 708, 709, 710, 1939	0.9230	0.9343	0.9221	0.8941	0.0261	0.0245	0.0263	0.0308
SIPI	697, 1129	0.4853	0.3401	0.3138	0.4134	0.0078	0.0088	0.0088	0.0083
VOPT	438, 440, 924	0.9364	0.9513	0.9628	0.9488	0.0940	0.0823	0.0722	0.0929
TCARI	743, 770, 776, 893	0.5482	0.6066	0.6552	0.6122	0.0265	0.0259	0.0243	0.0265
SAVI	440, 681, 1003	0.9400	0.9478	0.9699	0.9458	0.0239	0.0223	0.0169	0.0257
SR800550	373, 374, 587, 589, 590, 591, 1153	0.9854	0.9870	0.9175	0.9801	0.0599	0.0499	0.1035	0.0710
CIIRE	702, 704	0.9287	0.9342	0.9322	0.9302	0.0776	0.0747	0.0758	0.0772
LCI	422, 697, 698, 699, 700, 702	0.9807	0.9741	0.9841	0.9762	0.0153	0.0101	0.0121	0.0152
ARI	352, 378, 562, 564, 1138	0.9666	0.9467	0.8600	0.9185	0.1639	0.2512	1.5641	0.1988
SPRI2	374, 378, 589, 590, 591, 1153	0.9791	0.9813	0.9201	0.9750	0.0602	0.0525	0.1532	0.0700
BRI	355, 1269	0.3370	0.2623	0.4306	0.3188	0.0803	0.0901	0.0735	0.0958
SPADI	438, 459, 492, 648, 649, 651, 683, 694, 1133	0.9845	0.9983	0.9984	0.9816	0.0094	0.0030	0.0028	0.0119
GM1	374, 378, 589, 590, 591, 1153	0.9776	0.9787	0.9193	0.9747	0.0629	0.0571	0.1527	0.0542
RVI1	697, 698, 700, 1089	0.9628	0.9669	0.9356	0.9546	0.0929	0.0927	0.1250	0.1066
CRİ1	378, 1125	0.9596	0.9324	0.8462	0.9255	0.1960	0.4834	1.0937	0.2561
RVSI	779, 785, 789, 795, 798, 864	0.9746	0.9760	0.9797	0.9746	0.0047	0.0045	0.0042	0.0047
NDVI1	415, 430, 431, 435, 644, 683, 1001	0.9770	0.9892	0.9853	0.9787	0.0221	0.0154	0.0188	0.0246
NDVI3	704, 706, 707, 1933	0.9477	0.9522	0.9564	0.9260	0.0474	0.0431	0.0401	0.0560
NDVI6	691, 692, 693, 694, 695, 696, 1139	0.9785	0.9865	0.9827	0.9740	0.0149	0.0120	0.0138	0.0179
PSNDB	378, 426, 639, 641, 643, 645, 1158	0.9816	0.9920	0.9888	0.9799	0.0175	0.0113	0.0144	0.0211
LIC2	415, 431, 444, 651, 683, 684, 685, 942	0.9813	0.9940	0.9914	0.9847	0.0185	0.0106	0.0136	0.0189

Results for 1 nm at third dehydration stage

VIs	Inputs	R^2				$RMSE$			
		RF	MLP	SVR	kNN	RF	MLP	SVR	kNN
TM57	1319, 2267, 2269, 2270, 2271, 2273	0.9559	0.9705	0.9677	0.9553	0.0218	0.0179	0.0187	0.0232
FWBI	735, 738, 740, 747, 749, 2500	0.8707	0.8739	0.8875	0.7985	0.0025	0.0026	0.0024	0.0031
LWI	2059, 2500	0.8402	0.7912	0.8461	0.8409	0.0164	0.0201	0.0160	0.0162
SIWSI	764, 835, 852	0.5331	0.5681	0.6803	0.4356	0.0118	0.0115	0.0099	0.0143
VOG1	700, 701, 1000	0.9515	0.9579	0.9535	0.9160	0.0130	0.0131	0.0127	0.0171
SIPI	692, 970	0.5279	0.4364	0.5889	0.4837	0.0114	0.0131	0.0108	0.0135
VOPT	670, 675, 943	0.9506	0.9611	0.9727	0.9578	0.0770	0.0697	0.0572	0.0747
TCARI	888, 1990	0.5330	0.4450	0.5568	0.4614	0.0317	0.0353	0.0308	0.0370
SAVI	670, 672, 981	0.9552	0.9639	0.9775	0.9612	0.0207	0.0188	0.0146	0.0208
SR800550	359, 690, 691, 697, 1000	0.9664	0.9738	0.9463	0.9565	0.0687	0.0643	0.1116	0.0776
CIIRE	695, 696, 697, 698, 699, 1000, 1944	0.9568	0.9619	0.9572	0.9445	0.0401	0.0419	0.0400	0.0462
LCI	541, 695, 696, 697, 698, 1000	0.9749	0.9843	0.9820	0.9714	0.0149	0.0123	0.0130	0.0164
ARI	350, 356, 531, 537, 543, 547, 549, 998	0.9563	0.9494	0.9122	0.9275	0.1581	0.2084	0.5720	0.1678
SPRI2	350, 690, 691, 694, 1000	0.9667	0.9748	0.9443	0.9578	0.0611	0.0563	0.2780	0.0685
BRI	367, 368, 2245	0.6314	0.5312	0.6615	0.4830	0.0616	0.0726	0.0592	0.0813
SPADI	641, 647, 649, 682, 683, 684, 685, 1000	0.9799	0.9931	0.9930	0.9792	0.0111	0.0065	0.0066	0.0121
GM1	350, 690, 691, 693, 1000	0.9657	0.9672	0.9441	0.9571	0.0639	0.0653	0.1014	0.0714
RVI1	695, 696, 697, 698, 1000, 1937	0.9572	0.9646	0.9593	0.9486	0.0669	0.0655	0.0653	0.0746
CRİ1	350, 353, 373, 412, 688, 691, 1000	0.9691	0.8721	0.9800	0.9062	0.1796	1.0853	0.2332	0.2428
RVSI	795, 796, 801, 810, 817, 818, 834	0.9877	0.9910	0.9926	0.9859	0.0029	0.0026	0.0023	0.0032
NDVI1	681, 682, 683, 685, 996	0.9739	0.9803	0.9793	0.9730	0.0234	0.0204	0.0209	0.0251
NDVI3	701	0.9396	0.9593	0.9615	0.9556	0.0482	0.0407	0.0395	0.0421
NDVI6	692, 693, 694, 697, 1000	0.9721	0.9837	0.9814	0.9742	0.0162	0.0129	0.0133	0.0163
PSNDB	687, 688, 689, 691, 996	0.9776	0.9879	0.9859	0.9786	0.0187	0.0138	0.0148	0.0194
LIC2	679, 681, 682, 683, 685, 983	0.9761	0.9856	0.9848	0.9777	0.0210	0.0165	0.0169	0.0217

Results for 1 nm at fully dry stage

VIs	Inputs	R^2				$RMSE$			
		RF	MLP	SVR	kNN	RF	MLP	SVR	kNN
TM57	922, 1712, 2252, 2286, 2298	0.9967	0.9984	0.9878	0.9950	0.0239	0.0172	0.0462	0.0296
FWBI	355, 760, 821, 1143, 1381	0.9943	0.9970	0.9945	0.9833	0.0018	0.0013	0.0018	0.0030
LWI	922, 1577, 1704	0.9965	0.9968	0.9932	0.9950	0.0343	0.0333	0.0478	0.0407
SIWSI	846, 1386	0.9946	0.9929	0.9944	0.9929	0.0121	0.0139	0.0122	0.0138
VOG1	707, 859, 1741	0.9897	0.9841	0.9677	0.9845	0.0314	0.0391	0.0551	0.0382
SIPI	689, 690, 981, 1927	0.7499	0.7064	0.7287	0.6234	0.0082	0.0092	0.0085	0.0108
VOPT	418, 680, 787, 2311	0.9960	0.9994	0.9959	0.9938	0.0533	0.0221	0.0534	0.0657
TCARI	712, 715, 716, 1157, 1335, 1347, 1618, 1925	0.8883	0.8473	0.8068	0.8052	0.0225	0.0268	0.0293	0.0306
SAVI	652, 694, 885, 2110	0.9871	0.9861	0.9852	0.9836	0.0162	0.0167	0.0172	0.0184
SR800550	558, 580, 581, 586, 704, 1019	0.9903	0.9893	0.8972	0.9837	0.0495	0.0564	0.1222	0.0547
CIIRE	704, 708, 787, 2300	0.9940	0.9981	0.9858	0.9955	0.0784	0.0469	0.1223	0.0676
LCI	697, 698, 699, 700, 706, 943, 2474	0.9922	0.9956	0.9940	0.9922	0.0106	0.0079	0.0094	0.0109
ARI	350, 544, 549, 729, 733, 1143	0.9738	0.9840	0.8786	0.9513	0.5653	0.4502	1.2089	0.7702
SPRI2	571, 696, 697, 931, 1399	0.9824	0.9820	0.9139	0.9699	0.0610	0.0674	0.2384	0.0758
BRI	353, 354, 771, 961, 1403	0.8814	0.8492	0.8735	0.8109	0.0778	0.0885	0.0803	0.1010
SPADI	641, 642, 643, 644, 645, 646, 647, 648, 649, 650, 1001	0.9935	0.9980	0.9968	0.9952	0.0059	0.0033	0.0042	0.0052
GM1	576, 593, 696, 702, 937	0.9878	0.9860	0.9087	0.9812	0.0532	0.0622	0.2641	0.0595
RVI1	707, 822, 2300	0.9919	0.9956	0.9695	0.9932	0.0451	0.0398	0.1031	0.0429
CRİ1	391, 392, 696, 858, 1259	0.9725	0.9605	0.8570	0.9556	0.2532	0.4388	2.0972	0.2689
RVSI	708, 768, 769, 770, 1385	0.9984	0.9994	0.9994	0.9974	0.0050	0.0030	0.0032	0.0065
NDVI1	638, 639, 640, 642, 647, 938, 2474	0.9837	0.9851	0.9843	0.9840	0.0200	0.0197	0.0198	0.0201
NDVI3	700, 707, 915, 1293, 2310	0.9832	0.9927	0.9909	0.9797	0.0150	0.0099	0.0110	0.0164
NDVI6	696, 697, 698, 772, 963, 2310	0.9943	0.9989	0.9936	0.9923	0.0118	0.0052	0.0126	0.0138
PSNDB	593, 693, 694, 695, 932, 2474	0.9889	0.9893	0.9880	0.9878	0.0158	0.0156	0.0164	0.0169
LIC2	643, 922, 2474	0.9876	0.9885	0.9899	0.9836	0.0156	0.0150	0.0140	0.0190

Results for 1 nm combined stages

VIs	Inputs	R^2				$RMSE$			
		RF	MLP	SVR	kNN	RF	MLP	SVR	kNN
TM57	705, 1125, 2250, 2255, 2265, 2270, 2280, 2295, 2330, 2335	0.9532	0.9753	0.9681	0.9306	0.0408	0.0293	0.0345	0.0487
FWBI	355, 735, 740, 745, 1515, 2495	0.8525	0.8611	0.8871	0.8040	0.0008	0.0008	0.0007	0.0010
LWI	365, 1130, 1495, 1500, 1505, 2175	0.9609	0.9949	0.9815	0.9611	0.0484	0.0171	0.0352	0.0470
SIWSI	365, 1095, 1505, 1525	0.9406	0.9833	0.9754	0.9041	0.0108	0.0057	0.0069	0.0134
VOG1	355, 700, 705, 710, 1150	0.9447	0.9764	0.9796	0.9279	0.0363	0.0237	0.0222	0.0409
SIPI	350, 355, 360, 730	0.2710	0.4052	0.4964	0.3203	0.0042	0.0042	0.0036	0.0048
VOPT	515, 685, 770	0.9695	0.9984	0.9959	0.9754	0.1018	0.0214	0.0374	0.0814
TCARI	445, 725	0.7830	0.8193	0.8522	0.7283	0.0193	0.0180	0.0161	0.0223
SAVI	360, 485, 655, 665, 1135	0.9478	0.9895	0.9962	0.9492	0.0171	0.0077	0.0044	0.0168
SR800550	360, 530, 540, 545, 700, 1305	0.9745	0.9910	0.9469	0.9633	0.0588	0.0320	0.1209	0.0620
CIIRE	355, 700, 705, 710, 1150	0.9644	0.9872	0.9765	0.9594	0.0576	0.0326	0.0475	0.0561
LCI	385, 700, 1085	0.9702	0.9931	0.9953	0.9475	0.0122	0.0055	0.0045	0.0148
ARI	700, 705, 1315	0.7232	0.7077	0.7092	0.6529	0.2900	0.3056	0.2950	0.3445
SPRI2	360, 530, 535, 545, 700, 1305	0.9702	0.9880	0.9481	0.9564	0.3650	0.2401	0.5110	0.4371
BRI	700, 705, 1295	0.4967	0.2065	0.5733	0.2672	0.0778	0.1070	0.0724	0.1128
SPADI	380, 655, 770	0.9499	0.9936	0.9984	0.9382	0.0097	0.0036	0.0018	0.0111
GM1	360, 530, 535, 545, 700, 1305	0.9706	0.9887	0.9481	0.9570	0.0628	0.0375	0.1176	0.0671
RVI1	550, 700, 705, 1150	0.9718	0.9901	0.9652	0.9718	0.2023	0.1191	0.2316	0.2014
CRİ1	390, 395, 410, 415, 420, 425, 1320	0.9864	0.9778	0.8810	0.9667	0.6642	0.8478	2.1203	1.0342
RVSI	705, 785, 840, 1085	0.9788	0.9883	0.9934	0.9785	0.0069	0.0054	0.0039	0.0070
NDVI1	450, 655, 660, 770	0.9643	0.9947	0.9977	0.9564	0.0152	0.0057	0.0036	0.0162
NDVI3	700, 705, 710, 1115	0.9608	0.9937	0.9945	0.9700	0.0109	0.0044	0.0041	0.0094
NDVI6	360, 535, 560, 580, 695, 700, 1310	0.9567	0.9928	0.9947	0.9537	0.0144	0.0059	0.0049	0.0152
PSNDB	420, 435, 490, 610, 625, 630, 635, 640, 1315	0.9638	0.9906	0.9971	0.9659	0.0147	0.0075	0.0039	0.0146
LIC2	450, 655, 660, 770	0.9660	0.9941	0.9977	0.9581	0.0140	0.0056	0.0034	0.0150

Results for 5 nm fresh stage

VIs	Inputs	R^2				$RMSE$			
		RF	MLP	SVR	kNN	RF	MLP	SVR	kNN
TM57	365, 2250, 2310, 2325, 2330, 2345, 2365	0.9562	0.9490	0.9523	0.9301	0.0360	0.0410	0.0378	0.0453
FWBI	745, 750	0.9350	0.9210	0.9498	0.9138	0.0030	0.0034	0.0026	0.0034
LWI	355, 1455, 1460, 1470, 1475, 1485	0.9079	0.8878	0.9259	0.9077	0.0591	0.0729	0.0534	0.0595
SIWSI	765, 915, 1885, 1890, 2000	0.8868	0.9421	0.9416	0.9044	0.0169	0.0113	0.0118	0.0145
VOG1	360, 695, 700, 2150	0.7815	0.7088	0.7652	0.7245	0.0533	0.0622	0.0571	0.0607
SIPI	675, 680, 685, 1345	0.5754	0.2861	0.4312	0.4772	0.0052	0.0071	0.0056	0.0056
VOPT	680, 685, 690, 835, 870	0.9614	0.9981	0.9899	0.9648	0.1029	0.0216	0.0534	0.0995
TCARI	720	0.6579	0.6761	0.7115	0.6967	0.0235	0.0240	0.0217	0.0220
SAVI	680, 685, 940	0.8936	0.9206	0.9358	0.8942	0.0296	0.0259	0.0232	0.0296
SR800550	375, 380, 550, 555, 560, 565	0.9374	0.9301	0.8931	0.9325	0.8517	0.9138	1.1168	0.8814
CIIRE	365, 695, 700	0.8087	0.7823	0.8060	0.7933	0.1720	0.1912	0.1818	0.1781
LCI	380, 695, 700	0.9708	0.9740	0.9778	0.9711	0.0136	0.0131	0.0119	0.0137
ARI	550, 730,	0.9508	0.9440	0.8368	0.9074	0.8267	0.8967	1.4874	1.1241
SPRI2	380, 565, 575, 580	0.9351	0.9401	0.8804	0.9350	0.6596	0.6384	0.8985	0.6603
BRI	685, 1370	0.6451	0.5101	0.6301	0.6408	0.0759	0.0902	0.0759	0.0757
SPADI	645, 685, 1090	0.9921	0.9983	0.9979	0.9880	0.0048	0.0022	0.0025	0.0073
GM1	380, 560, 575, 580	0.9415	0.9422	0.8830	0.9341	0.6404	0.6403	0.9036	0.6795
RVI1	365, 690, 695, 915	0.8853	0.9280	0.8687	0.8354	0.2407	0.1908	0.2692	0.2864
CRİ1	380, 415, 845	0.9117	0.9253	0.8113	0.9113	0.9681	0.8800	1.3808	0.9698
RVSI	770, 775	0.9857	0.9869	0.9901	0.9801	0.0080	0.0075	0.0066	0.0096
NDVI1	685, 1090	0.9567	0.9623	0.9770	0.9533	0.0241	0.0225	0.0176	0.0259
NDVI3	360, 700, 705	0.8778	0.8704	0.8819	0.8638	0.0196	0.0205	0.0196	0.0205
NDVI6	695, 1090	0.9492	0.9574	0.9666	0.9423	0.0195	0.0180	0.0163	0.0214
PSNDB	690, 1090	0.9721	0.9752	0.9856	0.9683	0.0167	0.0157	0.0120	0.0192
LIC2	685, 1090	0.9703	0.9739	0.9856	0.9664	0.0180	0.0169	0.0126	0.0203

Results for 5 nm first dehydration stage

VIs	Inputs	R^2				$RMSE$			
		RF	MLP	SVR	kNN	RF	MLP	SVR	kNN
TM57	1090, 2160, 2165, 2170, 2255, 2260, 2265, 2435, 2480	0.9750	0.9850	0.9828	0.9800	0.0274	0.0212	0.0228	0.0263
FWBI	735, 740, 745, 750, 755, 760, 2245	0.8973	0.9038	0.9120	0.9094	0.0028	0.0028	0.0026	0.0027
LWI	355, 1100, 1470, 1480, 2075	0.9747	0.9924	0.9808	0.9798	0.0305	0.0168	0.0283	0.0288
SIWSI	360, 1025, 1890	0.7845	0.8461	0.8485	0.8042	0.0151	0.0130	0.0130	0.0145
VOG1	700, 705, 710, 715, 1950	0.9343	0.9319	0.9332	0.9286	0.0269	0.0276	0.0271	0.0281
SIPI	350, 355, 360, 870	0.3424	0.2320	0.3583	0.4572	0.0086	0.0098	0.0084	0.0079
VOPT	675, 680, 885,	0.9414	0.9652	0.9719	0.9482	0.0848	0.0670	0.0584	0.0813
TCARI	745, 755	0.6232	0.5951	0.7351	0.7035	0.0228	0.0234	0.0192	0.0201
SAVI	675, 680, 885	0.9534	0.9748	0.9834	0.9583	0.0193	0.0151	0.0116	0.0188
SR800550	555, 560, 565, 570, 1305	0.9802	0.9818	0.9400	0.9747	0.3729	0.3639	0.6623	0.4335
CIIRE	695, 700, 705, 710, 1100, 2490	0.9510	0.9537	0.9578	0.9487	0.0724	0.0749	0.0669	0.0757
LCI	355, 695, 700, 1100, 1930	0.9849	0.9950	0.9918	0.9731	0.0114	0.0068	0.0082	0.0159
ARI	360, 545, 550, 555, 560, 565, 570, 1305	0.9601	0.9569	0.8731	0.9569	0.5146	0.5408	0.9110	0.5365
SPRI2	560, 565, 570, 590, 1305	0.9760	0.9754	0.9402	0.9631	0.2984	0.3061	0.4771	0.3741
BRI	350, 355, 360, 1330	0.6615	0.5659	0.6306	0.6329	0.0658	0.0736	0.0675	0.0677
SPADI	470, 500, 640, 645, 650, 655, 660, 685, 1210	0.9916	0.9988	0.9981	0.9923	0.0056	0.0021	0.0027	0.0061
GM1	555, 560, 565, 590, 1305	0.9755	0.9768	0.9394	0.9623	0.3122	0.3091	0.4981	0.3923
RVI1	690, 695, 700, 705, 1100	0.9634	0.9616	0.9566	0.9566	0.0997	0.1080	0.1084	0.1099
CRİ1	400, 410, 910	0.9625	0.9345	0.8669	0.9565	0.4945	0.6905	0.9169	0.5339
RVSI	765, 770, 775, 780, 785, 790, 850, 880, 885	0.9671	0.9757	0.9788	0.9633	0.0056	0.0049	0.0044	0.0061
NDVI1	430, 435, 670, 675, 680, 685, 1195	0.9806	0.9845	0.9870	0.9810	0.0183	0.0167	0.0152	0.0188
NDVI3	705, 1095, 1930	0.9595	0.9692	0.9676	0.9430	0.0125	0.0115	0.0110	0.0153
NDVI6	555, 605, 635, 690, 695, 1245	0.9789	0.9887	0.9869	0.9768	0.0135	0.0104	0.0106	0.0151
PSNDB	430, 590, 640, 645, 650, 1200	0.9810	0.9905	0.9902	0.9840	0.0156	0.0113	0.0113	0.0155
LIC2	435, 440, 670, 675, 680, 685, 1195	0.9850	0.9902	0.9914	0.9862	0.0146	0.0121	0.0112	0.0150

Results for 5 nm second dehydration stage

VIs	Inputs	R^2				$RMSE$			
		RF	MLP	SVR	kNN	RF	MLP	SVR	kNN
TM57	2175, 2255, 2260, 2270, 2335	0.9517	0.9501	0.9578	0.9486	0.0319	0.0334	0.0299	0.0334
FWBI	750, 755, 760, 765, 800, 2235	0.8629	0.8422	0.8836	0.8627	0.0030	0.0034	0.0028	0.0030
LWI	1455, 1460, 1465, 2050, 2055	0.9578	0.9567	0.9616	0.9624	0.0322	0.0341	0.0312	0.0307
SIWSI	380, 1075, 1885, 1890	0.7088	0.6835	0.7344	0.7063	0.0147	0.0156	0.0141	0.0148
VOG1	705, 710, 1930	0.9193	0.9285	0.9220	0.9275	0.0267	0.0257	0.0263	0.0256
SIPI	695, 700	0.4262	0.3838	0.3393	0.4083	0.0084	0.0086	0.0082	0.0081
VOPT	430, 440, 925	0.9359	0.9509	0.9625	0.9456	0.0943	0.0826	0.0726	0.0946
TCARI	735, 745, 755, 770, 785, 900, 905	0.5286	0.6349	0.6569	0.5549	0.0270	0.0256	0.0243	0.0262
SAVI	440, 675, 1005	0.9372	0.9484	0.9698	0.9472	0.0243	0.0222	0.0170	0.0256
SR800550	365, 370, 580, 585, 590, 595, 1150	0.9844	0.9868	0.9158	0.9806	0.3132	0.2910	0.8813	0.3708
CIIRE	700, 705	0.9442	0.9323	0.9297	0.9372	0.0690	0.0758	0.0774	0.0738
LCI	695, 700, 1095	0.9833	0.9919	0.9911	0.9747	0.0129	0.0089	0.0092	0.0182
ARI	350, 355, 375, 555, 560, 565, 570, 1135	0.9670	0.9457	0.8619	0.9527	0.4414	0.5648	1.0135	0.5283
SPRI2	355, 370, 580, 585, 590, 595, 1150	0.9772	0.9801	0.9182	0.9731	0.2735	0.2568	0.6331	0.3072
BRI	355, 1270	0.3481	0.2620	0.4288	0.3108	0.0797	0.0902	0.0735	0.0793
SPADI	435, 440, 480, 640, 645, 650, 680, 695, 1135	0.9845	0.9984	0.9984	0.9816	0.0094	0.0029	0.0028	0.0119
GM1	355, 370, 580, 585, 590, 595, 1150	0.9769	0.9782	0.9162	0.9726	0.2889	0.2820	0.6577	0.3246
RVI1	695, 700, 1095	0.9656	0.9645	0.9362	0.9521	0.0894	0.0954	0.1235	0.1103
CRI1	370, 375, 1095	0.9450	0.9124	0.8363	0.9390	0.6658	0.8621	1.1172	0.7037
RVSI	795, 900	0.9758	0.9750	0.9795	0.9748	0.0045	0.0049	0.0042	0.0048
NDVI1	415, 420, 430, 435, 645, 685, 1005	0.9764	0.9887	0.9855	0.9792	0.0224	0.0158	0.0185	0.0242
NDVI3	705, 2065	0.9411	0.9427	0.9538	0.9417	0.0145	0.0142	0.0128	0.0146
NDVI6	690, 695, 1110	0.9793	0.9849	0.9813	0.9718	0.0146	0.0126	0.0141	0.0197
PSNDB	390, 415, 625, 630, 635, 645, 1155	0.9810	0.9912	0.9886	0.9795	0.0177	0.0118	0.0141	0.0211
LIC2	415, 430, 435, 650, 680, 685, 945	0.9811	0.9941	0.9910	0.9833	0.0186	0.0106	0.0138	0.0206

Results for 5 nm third dehydration stage

VIs	Inputs	R^2				$RMSE$			
		RF	MLP	SVR	kNN	RF	MLP	SVR	kNN
TM57	1315, 2260, 2265, 2270, 2275, 2280	0.9534	0.9704	0.9688	0.9541	0.0224	0.0180	0.0183	0.0234
FWBI	730, 735, 740, 745, 755, 2500	0.8721	0.8726	0.8854	0.8885	0.0025	0.0026	0.0024	0.0023
LWI	2060, 2500	0.8423	0.7904	0.8465	0.8417	0.0163	0.0202	0.0160	0.0162
SIWSI	760, 835, 870	0.5694	0.5696	0.6837	0.6463	0.0114	0.0115	0.0098	0.0105
VOG1	700, 1000	0.9522	0.9524	0.9529	0.9423	0.0129	0.0141	0.0128	0.0144
SIPI	690, 970	0.5230	0.4230	0.5845	0.5969	0.0114	0.0132	0.0109	0.0107
VOPT	670, 675, 945	0.9504	0.9608	0.9725	0.9577	0.0773	0.0700	0.0574	0.0748
TCARI	855, 1990	0.5471	0.4480	0.5608	0.4573	0.0312	0.0352	0.0308	0.0336
SAVI	670, 675, 975	0.9577	0.9649	0.9779	0.9609	0.0201	0.0186	0.0145	0.0208
SR800550	350, 590, 690, 695, 1000	0.9647	0.9753	0.9476	0.9562	0.3020	0.2606	0.3722	0.3395
CIIRE	695, 700, 1945	0.9527	0.9556	0.9456	0.9430	0.0419	0.0420	0.0450	0.0464
LCI	540, 555, 560, 690, 695, 700, 1000	0.9733	0.9853	0.9840	0.9740	0.0153	0.0118	0.0122	0.0156
ARI	350, 355, 375, 525, 540,<br>545, 550, 555, 560, 1000	0.9594	0.9621	0.9107	0.9487	0.3103	0.3005	0.4694	0.3495
SPRI2	350, 630, 690, 695, 1000	0.9710	0.9817	0.9437	0.9583	0.1969	0.1621	0.2791	0.2384
BRI	355, 375, 385, 390, 1680, 2245	0.6580	0.5431	0.6512	0.6650	0.0593	0.0705	0.0602	0.0591
SPADI	685, 1000	0.9770	0.9905	0.9927	0.9732	0.0119	0.0077	0.0067	0.0145
GM1	350, 685, 690, 695, 1000	0.9699	0.9826	0.9431	0.9582	0.2110	0.1642	0.2952	0.2498
RVI1	690, 695, 700, 1000, 1935	0.9626	0.9660	0.9587	0.9390	0.0627	0.0622	0.0658	0.0804
CRİ1	350, 365, 375, 430, 690, 1000	0.9714	0.9804	0.8725	0.9362	0.2769	0.2324	0.5774	0.4088
RVSI	795, 800, 810, 815, 835	0.9879	0.9904	0.9926	0.9859	0.0029	0.0028	0.0023	0.0032
NDVI1	645, 675, 680, 685, 1000	0.9730	0.9822	0.9788	0.9707	0.0238	0.0194	0.0212	0.0259
NDVI3	700, 705	0.9489	0.9565	0.9600	0.9519	0.0109	0.0101	0.0098	0.0106
NDVI6	630, 690, 695, 700, 1000	0.9723	0.9841	0.9817	0.9722	0.0162	0.0127	0.0132	0.0170
PSNDB	645, 685, 690, 695, 1000	0.9770	0.9891	0.9852	0.9764	0.0189	0.0132	0.0152	0.0204
LIC2	650, 670, 675, 680, 685, 1000	0.9773	0.9860	0.9844	0.9779	0.0205	0.0162	0.0171	0.0215

Results for 5 nm fully dry stage

VIs	Inputs	R^2				$RMSE$			
		RF	MLP	SVR	kNN	RF	MLP	SVR	kNN
TM57	920, 1710, 2255, 2285	0.9968	0.9984	0.9876	0.9943	0.0237	0.0172	0.0466	0.0316
FWBI	355, 760, 790, 1140, 1380	0.9939	0.9964	0.9919	0.9881	0.0018	0.0014	0.0021	0.0026
LWI	920, 1575, 1705	0.9965	0.9968	0.9932	0.9966	0.0343	0.0336	0.0477	0.0336
SIWSI	850, 1385	0.9947	0.9931	0.9946	0.9953	0.0119	0.0137	0.0120	0.0113
VOG1	695, 700, 705, 855, 860, 895, 1210, 1215, 1745	0.9890	0.9965	0.9918	0.9856	0.0325	0.0184	0.0278	0.0367
SIPI	685, 690, 695, 980, 1925, 1930	0.7604	0.7224	0.7267	0.7505	0.0080	0.0089	0.0085	0.0081
VOPT	415, 680, 785, 1095, 2310	0.9964	0.9996	0.9960	0.9917	0.0517	0.0179	0.0533	0.0763
TCARI	705, 710, 715, 1135, 1335, 1345, 1610, 1925	0.9003	0.8641	0.8105	0.8777	0.0213	0.0252	0.0289	0.0236
SAVI	470, 695, 885, 1510	0.9867	0.9840	0.9844	0.9823	0.0165	0.0179	0.0177	0.0191
SR800550	565, 575, 580, 585, 705, 1030	0.9908	0.9898	0.8970	0.9827	0.2990	0.3252	1.0386	0.4131
CIIRE	700, 705, 790, 2300	0.9932	0.9982	0.9860	0.9945	0.0832	0.0458	0.1216	0.0743
LCI	585, 695, 700, 705, 940, 2305	0.9922	0.9961	0.9943	0.9918	0.0106	0.0075	0.0092	0.0110
ARI	350, 540, 545, 550, 730, 735, 1145	0.9753	0.9859	0.8840	0.9700	0.5488	0.4173	1.1877	0.6047
SPRI2	595, 695, 700, 925	0.9865	0.9832	0.9114	0.9766	0.3155	0.3703	0.8284	0.4148
BRI	350, 355, 770, 965, 1400	0.8839	0.8483	0.8706	0.8847	0.0770	0.0889	0.0811	0.0769
SPADI	645, 940, 1910	0.9933	0.9982	0.9965	0.9855	0.0061	0.0031	0.0044	0.0097
GM1	575, 595, 695, 700, 935	0.9874	0.9859	0.9097	0.9818	0.3017	0.3253	0.8322	0.3630
RVI1	695, 700, 705, 775, 825, 1145, 2300	0.9948	0.9997	0.9797	0.9922	0.1236	0.0297	0.2468	0.1494
CRİ1	390, 395, 695, 855, 1265	0.9761	0.9671	0.8580	0.9698	0.7396	0.8821	1.7497	0.8210
RVSI	705, 765, 770, 775, 1385	0.9983	0.9992	0.9991	0.9972	0.0051	0.0035	0.0037	0.0066
NDVI1	630, 635, 640, 645, 650, 940, 2475,	0.9844	0.9862	0.9841	0.9841	0.0196	0.0192	0.0199	0.0201
NDVI3	700, 705, 915, 1295, 2310	0.9827	0.9930	0.9910	0.9837	0.0152	0.0097	0.0110	0.0147
NDVI6	695, 700, 770, 960, 2310	0.9948	0.9990	0.9936	0.9914	0.0114	0.0051	0.0127	0.0147
PSNDB	595, 630, 635, 695, 935, 2475	0.9886	0.9913	0.9883	0.9882	0.0160	0.0140	0.0163	0.0165
LIC2	630, 635, 640, 645, 650, 655, 955, 1475	0.9881	0.9915	0.9891	0.9876	0.0152	0.0129	0.0146	0.0159

Results for 5 nm combined stages

VIs	Inputs	R^2				$RMSE$			
		RF	MLP	SVR	kNN	RF	MLP	SVR	kNN
TM57	710, 1090, 2270, 2330	0.9510	0.9617	0.9675	0.9353	0.0418	0.0366	0.0346	0.0515
FWBI	360, 740, 750, 1520	0.8483	0.8501	0.8817	0.8734	0.0008	0.0009	0.0008	0.0008
LWI	360, 1130, 1490, 1500, 1510, 2160	0.9617	0.9965	0.9836	0.9650	0.0483	0.0141	0.0325	0.0489
SIWSI	360, 1090, 1500, 1520	0.9393	0.9866	0.9736	0.9349	0.0110	0.0052	0.0071	0.0116
VOG1	550, 700, 710, 1110	0.9475	0.9838	0.9829	0.9582	0.0359	0.0200	0.0204	0.0349
SIPI	350, 360, 730	0.2490	0.4248	0.5092	0.3392	0.0043	0.0041	0.0036	0.0040
VOPT	510, 680, 770	0.9704	0.9988	0.9959	0.9693	0.1012	0.0189	0.0378	0.1038
TCARI	720, 730	0.7398	0.6273	0.7271	0.7453	0.0209	0.0265	0.0215	0.0207
SAVI	440, 660, 670, 1140	0.9472	0.9865	0.9955	0.9479	0.0173	0.0086	0.0049	0.0193
SR800550	360, 530, 540, 560, 700, 1310	0.9748	0.9903	0.9438	0.9755	0.3646	0.2262	0.5677	0.4132
CIIRE	550, 700, 710, 1110	0.9659	0.9927	0.9794	0.9778	0.1182	0.0557	0.0926	0.1076
LCI	380, 700, 1090	0.9687	0.9927	0.9953	0.9426	0.0123	0.0057	0.0045	0.0179
ARI	560, 700, 1310	0.7709	0.8341	0.7301	0.7352	0.2653	0.2360	0.2847	0.2827
SPRI2	360, 530, 540, 550, 700, 1300	0.9718	0.9873	0.9471	0.9744	0.3555	0.2462	0.5190	0.3880
BRI	350, 700, 710, 1320	0.7162	0.7504	0.8054	0.7063	0.0611	0.0583	0.0524	0.0621
SPADI	380, 650, 770	0.9503	0.9943	0.9986	0.9241	0.0097	0.0034	0.0017	0.0130
GM1	360, 530, 540, 550, 700, 1300	0.9716	0.9880	0.9479	0.9747	0.3484	0.2310	0.4974	0.3788
RVI1	540, 550, 700, 1300	0.9707	0.9845	0.9424	0.9746	0.2058	0.1499	0.2920	0.2187
CRİ1	380, 390, 410, 420, 430, 440, 1320	0.9893	0.9837	0.8782	0.9756	0.5911	0.7266	2.7280	0.9070
RVSI	700, 790, 840, 1090	0.9753	0.9785	0.9886	0.9772	0.0075	0.0072	0.0051	0.0078
NDVI1	450, 650, 660, 770	0.9647	0.9946	0.9977	0.9513	0.0151	0.0057	0.0036	0.0198
NDVI3	550, 700, 710, 1130	0.9582	0.9934	0.9944	0.9600	0.0114	0.0044	0.0041	0.0122
NDVI6	590, 700, 1090	0.9658	0.9966	0.9978	0.9517	0.0133	0.0042	0.0032	0.0170
PSNDB	420, 450, 470, 490, 610, 620, 630, 640, 1320	0.9633	0.9895	0.9968	0.9566	0.0147	0.0079	0.0041	0.0177
LIC2	450, 650, 660, 770	0.9663	0.9943	0.9977	0.9504	0.014	0.0055	0.0034	0.0188

Results for 10 nm fresh stage

VIs	Inputs	R^2				$RMSE$			
		RF	MLP	SVR	kNN	RF	MLP	SVR	kNN
TM57	360, 2250, 2260, 2310, 2330, 2350, 2400, 2410	0.9615	0.9491	0.9512	0.9610	0.0338	0.0400	0.0381	0.0352
FWBI	350, 750	0.9352	0.9194	0.9476	0.9210	0.0030	0.0034	0.0027	0.0034
LWI	350, 1450, 1460, 1470, 1480, 1490	0.9067	0.8846	0.9254	0.9236	0.0595	0.0741	0.0535	0.0544
SIWSI	760, 900, 1890, 2000, 2010	0.8855	0.9367	0.9271	0.9065	0.0168	0.0120	0.0130	0.0164
VOG1	360, 700, 2150	0.7516	0.7288	0.7708	0.7494	0.0565	0.0600	0.0566	0.0566
SIPI	660, 670, 680, 1350	0.6061	0.2806	0.4354	0.4869	0.0050	0.0071	0.0056	0.0055
VOPT	670, 680, 690, 840, 860	0.9634	0.9979	0.9865	0.9639	0.1008	0.0224	0.0610	0.0993
TCARI	720	0.6579	0.6761	0.7115	0.6967	0.0235	0.0240	0.0217	0.0220
SAVI	680, 690, 940	0.8969	0.9181	0.9343	0.8977	0.0293	0.0263	0.0235	0.0293
SR800550	360, 380, 540, 550, 560, 570, 580	0.9549	0.9589	0.8895	0.9459	0.7250	0.7034	1.1329	0.7928
CIIRE	360, 690, 700	0.7973	0.7421	0.8035	0.7921	0.1764	0.2029	0.1813	0.1788
LCI	380, 700	0.9604	0.9596	0.9772	0.9667	0.0158	0.0167	0.0121	0.0147
ARI	550, 730	0.9508	0.9440	0.8368	0.9074	0.8267	0.8967	1.4874	1.1241
SPRI2	380, 560, 580	0.9336	0.9404	0.8827	0.9231	0.6659	0.6361	0.8916	0.7172
BRI	690, 1370	0.6589	0.5113	0.6306	0.6542	0.0746	0.0902	0.0758	0.0745
SPADI	650, 690, 1090	0.9918	0.9983	0.9977	0.9871	0.0049	0.0022	0.0026	0.0075
GM1	380, 560, 570, 580	0.9392	0.9408	0.8842	0.9328	0.6519	0.6472	0.8982	0.6862
RVI1	380, 690, 700	0.7931	0.7760	0.8015	0.7868	0.3123	0.3393	0.3190	0.3164
CRI1	380, 400, 860	0.8986	0.9223	0.8100	0.9020	1.0338	0.8955	1.3807	1.0179
RVSI	770, 900	0.9845	0.9873	0.9887	0.9760	0.0084	0.0074	0.0072	0.0109
NDVI1	680, 1090	0.9586	0.9652	0.9769	0.9519	0.0236	0.0216	0.0177	0.0264
NDVI3	360, 700, 710	0.8772	0.8735	0.8741	0.8611	0.0196	0.0204	0.0202	0.0207
NDVI6	360, 690, 1090	0.9386	0.9580	0.9575	0.9271	0.0215	0.0178	0.0180	0.0236
PSNDB	690, 1090	0.9721	0.9752	0.9856	0.9683	0.0167	0.0157	0.0120	0.0192
LIC2	680, 1090	0.9720	0.9763	0.9853	0.9650	0.0175	0.0161	0.0127	0.0207

Results for 10 nm first stage

VIs	Inputs	R^2				$RMSE$			
		RF	MLP	SVR	kNN	RF	MLP	SVR	kNN
TM57	1090, 2160, 2170, 2180, 2250, 2260, 2270, 2450, 2480	0.9754	0.9853	0.9830	0.9796	0.0272	0.0210	0.0227	0.0266
FWBI	730, 740, 750, 760, 770, 2240	0.8964	0.9030	0.9122	0.9025	0.0028	0.0028	0.0026	0.0028
LWI	360, 1100, 1470, 1480, 2070	0.9757	0.9919	0.9807	0.9776	0.0300	0.0174	0.0285	0.0299
SIWSI	360, 1030, 1890	0.7987	0.8455	0.8490	0.8022	0.0147	0.0131	0.0130	0.0146
VOG1	700, 710, 720, 1960	0.9318	0.9743	0.9334	0.9175	0.0274	0.0181	0.0271	0.0301
SIPI	350, 910	0.3151	0.4166	0.3642	0.4072	0.0090	0.0083	0.0083	0.0083
VOPT	670, 680, 890	0.9368	0.9607	0.9686	0.9428	0.0874	0.0711	0.0619	0.0851
TCARI	750	0.6325	0.5968	0.7300	0.6940	0.0229	0.0238	0.0193	0.0204
SAVI	670, 680, 890	0.9496	0.9719	0.9806	0.9550	0.0200	0.0159	0.0126	0.0195
SR800550	530, 550, 560, 570, 1300	0.9805	0.9829	0.9367	0.9762	0.3692	0.3535	0.6769	0.4210
CIIRE	690, 700, 710, 1100	0.9535	0.9491	0.9572	0.9471	0.0706	0.0775	0.0675	0.0761
LCI	540, 700, 1100, 1930	0.9845	0.9950	0.9919	0.9767	0.0115	0.0069	0.0082	0.0152
ARI	360, 530, 540, 550, 560, 570, 580, 1310	0.9603	0.9578	0.8714	0.9588	0.5135	0.5352	0.9161	0.5247
SPRI2	560, 570, 580, 590, 1310	0.9754	0.9753	0.9404	0.9682	0.3014	0.3061	0.4762	0.3490
BRI	350, 1360	0.6214	0.5194	0.6280	0.5827	0.0705	0.0774	0.0676	0.0717
SPADI	440, 630, 640, 650, 660, 670, 680, 1210	0.9929	0.9987	0.9981	0.9921	0.0051	0.0022	0.0027	0.0063
GM1	560, 570, 580, 590, 1310	0.9751	0.9762	0.9398	0.9687	0.3146	0.3127	0.4986	0.3590
RVI1	700, 1100	0.9488	0.9622	0.9588	0.9467	0.1172	0.1070	0.1057	0.1217
CRİ1	400, 410, 910	0.9625	0.9345	0.8669	0.9565	0.4945	0.6905	0.9169	0.5339
RVSI	760, 770, 780, 790, 850, 900	0.9683	0.9811	0.9785	0.9635	0.0055	0.0044	0.0045	0.0061
NDVI1	430, 450, 650, 660, 670, 680, 1190	0.9802	0.9843	0.9865	0.9789	0.0185	0.0168	0.0155	0.0197
NDVI3	700, 710, 1100, 1930	0.9571	0.9696	0.9727	0.9485	0.0128	0.0112	0.0102	0.0146
NDVI6	590, 600, 610, 690, 700, 1140	0.9750	0.9854	0.9862	0.9779	0.0147	0.0118	0.0110	0.0146
PSNDB	430, 590, 630, 640, 650, 1200	0.9810	0.9901	0.9902	0.9836	0.0156	0.0116	0.0113	0.0156
LIC2	440, 450, 650, 660, 670, 680, 1190	0.9850	0.9900	0.9913	0.9840	0.0147	0.0123	0.0113	0.0159

Results for 10 nm second stage

VIs	Inputs	R^2				$RMSE$			
		RF	MLP	SVR	kNN	RF	MLP	SVR	kNN
TM57	2180, 2260, 2270, 2320	0.9480	0.9387	0.9576	0.9486	0.0330	0.0371	0.0299	0.0334
FWBI	740, 750, 760, 780, 790, 800, 2240	0.8669	0.8430	0.8825	0.8549	0.0029	0.0034	0.0028	0.0031
LWI	1450, 1460, 1470, 2050, 2060	0.9572	0.9565	0.9614	0.9619	0.0324	0.0341	0.0314	0.0309
SIWSI	1070, 1890, 1980	0.7301	0.6560	0.7527	0.7153	0.0142	0.0164	0.0137	0.0146
VOG1	710, 1930	0.8885	0.8919	0.9132	0.9037	0.0312	0.0308	0.0278	0.0293
SIPI	700	0.3861	0.4098	0.3894	0.3864	0.0088	0.0084	0.0081	0.0083
VOPT	430, 440, 930	0.9368	0.9479	0.9608	0.9459	0.0937	0.0849	0.0741	0.0949
TCARI	740, 750, 760, 770, 780, 900, 910	0.5308	0.6072	0.6551	0.5568	0.0268	0.0264	0.0243	0.0262
SAVI	440, 680, 1010	0.9365	0.9467	0.9685	0.9455	0.0245	0.0225	0.0173	0.0259
SR800550	350, 370, 570, 580, 590, 600, 1150	0.9851	0.9885	0.9152	0.9766	0.3075	0.2691	0.8915	0.4020
CIIRE	700, 1930	0.9491	0.9330	0.9313	0.9437	0.0660	0.0758	0.0773	0.0722
LCI	540, 700, 1110	0.9811	0.9917	0.9915	0.9751	0.0136	0.0090	0.0089	0.0183
ARI	350, 560, 570, 1110	0.9667	0.9424	0.8634	0.9506	0.4429	0.5804	0.9757	0.5440
SPRI2	350, 370, 580, 590, 600, 610, 1150	0.9792	0.9812	0.9166	0.9743	0.2611	0.2488	0.6351	0.3031
BRI	350, 1270	0.4154	0.2435	0.4262	0.3375	0.0772	0.0909	0.0740	0.0785
SPADI	440, 450, 480, 600, 630, 640, 650, 680, 1130	0.9845	0.9977	0.9985	0.9820	0.0095	0.0034	0.0028	0.0118
GM1	350, 370, 580, 590, 600, 610, 1150	0.9791	0.9800	0.9142	0.9740	0.2743	0.2700	0.6647	0.3186
RVI1	700, 1110	0.9529	0.9617	0.9374	0.9423	0.1038	0.0950	0.1202	0.1203
CRİ1	370, 1110	0.9313	0.8980	0.8395	0.9254	0.7408	0.9112	1.1061	0.7747
RVSI	770, 780, 790, 800, 810, 890	0.9757	0.9746	0.9788	0.9735	0.0046	0.0047	0.0043	0.0048
NDVI1	420, 430, 440, 450, 650, 680, 1160	0.9764	0.9886	0.9853	0.9783	0.0224	0.0157	0.0184	0.0248
NDVI3	700, 710	0.9526	0.9416	0.9544	0.9511	0.0130	0.0144	0.0127	0.0136
NDVI6	690, 1110	0.9756	0.9685	0.9762	0.9564	0.0158	0.0180	0.0156	0.0233
PSNDB	390, 420, 590, 630, 640, 690, 1150	0.9803	0.9911	0.9881	0.9774	0.0180	0.0119	0.0142	0.0215
LIC2	420, 430, 440, 650, 660, 680, 960	0.9810	0.9937	0.9908	0.9836	0.0186	0.0108	0.0138	0.0203

Results for 10 nm third stage

VIs	Inputs	R^2				$RMSE$			
		RF	MLP	SVR	kNN	RF	MLP	SVR	kNN
TM57	1320, 2260, 2270, 2280, 2290, 2300	0.9512	0.9709	0.9688	0.9568	0.0229	0.0178	0.0183	0.0227
FWBI	730, 740, 750, 760, 2500,	0.8668	0.8822	0.8844	0.8875	0.0026	0.0026	0.0024	0.0023
LWI	2060, 2500	0.8423	0.7904	0.8465	0.8417	0.0163	0.0202	0.0160	0.0162
SIWSI	750, 760, 770, 830, 860, 870, 880	0.5988	0.5357	0.6828	0.6391	0.0110	0.0119	0.0099	0.0105
VOG1	700, 1000	0.9522	0.9524	0.9529	0.9423	0.0129	0.0141	0.0128	0.0144
SIPI	690, 970	0.5230	0.4230	0.5845	0.5969	0.0114	0.0132	0.0109	0.0107
VOPT	670, 680, 950	0.9499	0.9582	0.9714	0.9546	0.0776	0.0720	0.0586	0.0773
TCARI	870, 1990	0.5366	0.4464	0.5590	0.4922	0.0316	0.0353	0.0309	0.0326
SAVI	660, 670, 970	0.9561	0.9654	0.9779	0.9609	0.0205	0.0184	0.0145	0.0209
SR800550	350, 570, 590, 690, 1000	0.9665	0.9742	0.9458	0.9577	0.2942	0.2619	0.3814	0.3346
CIIRE	700, 1940	0.9460	0.9331	0.9417	0.9307	0.0448	0.0498	0.0466	0.0511
LCI	690, 700, 1000	0.9765	0.9831	0.9826	0.9707	0.0145	0.0126	0.0126	0.0167
ARI	350, 380, 530, 540, 550, 560, 1000	0.9611	0.9650	0.9106	0.9423	0.3033	0.2942	0.4697	0.3695
SPRI2	350, 590, 630, 690, 1000	0.9687	0.9734	0.9429	0.9615	0.2045	0.1907	0.2813	0.2308
BRI	370, 380, 390, 1680, 2110	0.6264	0.5232	0.6488	0.6390	0.0616	0.0720	0.0604	0.0608
SPADI	600, 640, 650, 660, 670, 680, 1000	0.9811	0.9928	0.9929	0.9796	0.0108	0.0067	0.0066	0.0120
GM1	350, 590, 630, 690, 1000	0.9664	0.9698	0.9430	0.9592	0.2225	0.2132	0.2938	0.2484
RVI1	350, 630, 690, 700, 1000, 1930	0.9606	0.9674	0.9586	0.9345	0.0644	0.0630	0.0658	0.0835
CRİ1	350, 370, 380, 430, 690, 1000	0.9709	0.9810	0.8713	0.9389	0.2785	0.2286	0.5810	0.4018
RVSI	770, 780, 790, 800, 810, 820, 830, 840, 850	0.9894	0.9908	0.9922	0.9881	0.0028	0.0026	0.0023	0.0031
NDVI1	660, 670, 680, 690, 1000	0.9736	0.9829	0.9784	0.9717	0.0235	0.0190	0.0214	0.0255
NDVI3	700, 1000	0.9546	0.9634	0.9635	0.9475	0.0103	0.0097	0.0092	0.0113
NDVI6	590, 630, 690, 700, 1000	0.9705	0.9836	0.9802	0.9690	0.0167	0.0128	0.0138	0.0178
PSNDB	630, 640, 650, 690, 1000	0.9774	0.9877	0.9853	0.9783	0.0187	0.0139	0.0152	0.0197
LIC2	650, 660, 670, 680, 690, 1000	0.9769	0.9864	0.9842	0.9769	0.0207	0.0160	0.0173	0.0220

Results for 10 nm fully dry stage

VIs	Inputs	R^2				$RMSE$			
		RF	MLP	SVR	kNN	RF	MLP	SVR	kNN
TM57	920, 1710, 2250, 2290, 2300	0.9967	0.9984	0.9879	0.9951	0.0241	0.0168	0.0461	0.0293
FWBI	360, 760, 790, 1140, 1380,	0.9939	0.9963	0.9919	0.9875	0.0018	0.0014	0.0021	0.0026
LWI	920, 1570, 1710	0.9965	0.9969	0.9931	0.9967	0.0340	0.0328	0.0481	0.0335
SIWSI	830, 1390	0.9941	0.9921	0.9934	0.9942	0.0126	0.0146	0.0133	0.0124
VOG1	700, 860, 1740	0.9864	0.9762	0.9601	0.9852	0.0359	0.0474	0.0616	0.0372
SIPI	640, 680, 690, 980, 1920, 1930	0.7582	0.7591	0.7399	0.7528	0.0080	0.0084	0.0084	0.0081
VOPT	500, 680, 790, 2310	0.9961	0.9994	0.9961	0.9942	0.0527	0.0215	0.0522	0.0639
TCARI	710, 720, 1140, 1160, 1610, 1680, 1950	0.8874	0.8403	0.8018	0.8757	0.0226	0.0276	0.0297	0.0237
SAVI	640, 650, 880, 2310	0.9869	0.9866	0.9872	0.9859	0.0163	0.0164	0.0160	0.0170
SR800550	560, 570, 580, 590, 700, 1030	0.9903	0.9883	0.8981	0.9835	0.3071	0.3480	1.0315	0.4008
CIIRE	700, 790, 2300	0.9913	0.9880	0.9653	0.9906	0.0943	0.1127	0.1904	0.0971
LCI	700, 930, 2310	0.9926	0.9953	0.9937	0.9882	0.0105	0.0083	0.0095	0.0136
ARI	350, 540, 550, 560, 730, 740, 1140	0.9761	0.9873	0.8940	0.9704	0.5398	0.3961	1.1443	0.6013
SPRI2	570, 600, 700, 920	0.9885	0.9871	0.9114	0.9810	0.2916	0.3234	0.8331	0.3749
BRI	350, 360, 770, 970, 1400	0.8823	0.8515	0.8708	0.8865	0.0775	0.0888	0.0810	0.0763
SPADI	610, 620, 630, 640, 650, 660, 670, 690, 1010	0.9938	0.9981	0.9968	0.9945	0.0057	0.0032	0.0042	0.0056
GM1	570, 590, 700, 940	0.9880	0.9856	0.9083	0.9803	0.2943	0.3323	0.8357	0.3784
RVI1	700, 830, 2300	0.9931	0.9916	0.9546	0.9919	0.1426	0.1634	0.3690	0.1522
CRİ1	380, 390, 640, 860, 1260	0.9771	0.9690	0.8567	0.9672	0.7218	0.8750	1.7567	0.8552
RVSI	700, 760, 770, 780, 1380	0.9981	0.9988	0.9986	0.9969	0.0055	0.0043	0.0047	0.0070
NDVI1	620, 630, 640, 650, 690, 940, 2480	0.9848	0.9867	0.9839	0.9838	0.0194	0.0188	0.0200	0.0203
NDVI3	600, 700, 920, 1300, 2310	0.9816	0.9939	0.9899	0.9776	0.0157	0.0090	0.0116	0.0172
NDVI6	630, 700, 770, 960, 2310	0.9935	0.9989	0.9933	0.9903	0.0126	0.0052	0.0129	0.0155
PSNDB	600, 630, 640, 700, 920, 1400	0.9906	0.9943	0.9906	0.9889	0.0146	0.0113	0.0147	0.0160
LIC2	620, 630, 640, 650, 660, 690, 950, 1480	0.9888	0.9919	0.9891	0.9875	0.0148	0.0126	0.0146	0.0160

Results for 10 nm combined stages

VIs	Inputs	R^2				$RMSE$			
		RF	MLP	SVR	kNN	RF	MLP	SVR	kNN
TM57	1130, 2170, 2250, 2270, 2290, 2310, 2330, 2350	0.9526	0.9799	0.9656	0.9475	0.0411	0.0265	0.0363	0.0447
FWBI	350, 730, 750, 770, 1530	0.8380	0.8486	0.8823	0.8602	0.0009	0.0008	0.0008	0.0008
LWI	350, 1110, 1490, 1510, 1530, 2190	0.9609	0.9973	0.9902	0.9663	0.0486	0.0126	0.0245	0.0489
SIWSI	350, 750, 10,90, 1490, 1510, 1530, 2210	0.9475	0.9964	0.9881	0.9496	0.0106	0.0028	0.0048	0.0105
VOG1	530, 550, 710, 1110	0.9381	0.9781	0.9807	0.9570	0.0388	0.0229	0.0214	0.0354
SIPI	350, 370, 730	0.2784	0.3775	0.4511	0.3993	0.0044	0.0043	0.0037	0.0039
VOPT	510, 690, 770	0.9696	0.9978	0.9961	0.9657	0.0997	0.0250	0.0356	0.1079
TCARI	450, 730	0.6869	0.6916	0.7474	0.6982	0.0227	0.0231	0.0205	0.0221
SAVI	490, 650, 670, 1130	0.9521	0.9884	0.9959	0.9481	0.0170	0.0082	0.0048	0.0191
SR800550	530, 550, 570, 1290	0.9803	0.9921	0.9419	0.9774	0.3237	0.2018	0.5784	0.3984
CIIRE	550, 570, 710, 1110	0.9586	0.9930	0.9796	0.9762	0.1307	0.0550	0.0920	0.1106
LCI	530, 550, 570, 590, 710, 1290	0.9538	0.9917	0.9932	0.9547	0.0143	0.0060	0.0054	0.0158
ARI	690, 710, 1110	0.6776	0.6782	0.7110	0.7162	0.3094	0.3209	0.2931	0.2910
SPRI2	530, 550, 570, 1290	0.9800	0.9896	0.9432	0.9747	0.3033	0.2159	0.5309	0.3834
BRI	350, 690, 710, 1310	0.7489	0.7650	0.8027	0.7484	0.0581	0.0567	0.0528	0.0585
SPADI	370, 650, 770	0.9486	0.9938	0.9987	0.9151	0.0100	0.0035	0.0017	0.0138
GM1	530, 550, 570, 1290	0.9793	0.9900	0.9451	0.9750	0.3007	0.2060	0.5076	0.3741
RVI1	530, 550, 710, 1110	0.9652	0.9932	0.9690	0.9795	0.2263	0.1023	0.2179	0.1940
CRİ1	390, 410, 430, 450, 470, 1310	0.9901	0.9839	0.8767	0.9731	0.5686	0.7231	2.1845	0.9541
RVSI	550, 790, 830, 1090	0.9742	0.9775	0.9871	0.9740	0.0076	0.0074	0.0054	0.0081
NDVI1	430, 450, 470, 490, 650, 670, 1310, 1950	0.9603	0.9906	0.9949	0.9496	0.0159	0.0077	0.0054	0.0196
NDVI3	530, 550, 710, 1110	0.9491	0.9933	0.9941	0.9598	0.0125	0.0045	0.0042	0.0123
NDVI6	530, 550, 570, 590, 1310	0.9535	0.9882	0.9906	0.9489	0.0149	0.0073	0.0065	0.0174
PSNDB	410, 450, 470, 590, 610, 630, 650, 670, 1310	0.9639	0.9914	0.9970	0.9576	0.0145	0.0072	0.0040	0.0176
LIC2	430, 450, 470, 490, 630, 650, 670, 1310, 1950	0.9612	0.9937	0.9960	0.9490	0.0148	0.0059	0.0045	0.0185

Results for 20 nm fresh stage

VIs	Inputs	R^2				$RMSE$			
		RF	MLP	SVR	kNN	RF	MLP	SVR	kNN
TM57	370, 2310, 2350	0.9542	0.9519	0.9504	0.9602	0.0368	0.0398	0.0383	0.0355
FWBI	350, 750	0.9352	0.9194	0.9476	0.9210	0.0030	0.0034	0.0027	0.0034
LWI	350,1430, 1450, 1470, 1490	0.9093	0.8965	0.9244	0.9192	0.0587	0.0694	0.0539	0.0557
SIWSI	770, 890, 1890, 1990, 2010	0.8855	0.9460	0.9348	0.8977	0.0167	0.0110	0.0124	0.0169
VOG1	350, 370, 690, 2130, 2150	0.7366	0.6833	0.7395	0.7313	0.0578	0.0640	0.0592	0.0583
SIPI	650, 670, 690, 1350	0.5916	0.2872	0.4274	0.4904	0.0051	0.0071	0.0056	0.0055
VOPT	650, 670, 690, 830, 870	0.9633	0.9987	0.9911	0.9662	0.1005	0.0176	0.0507	0.0977
TCARI	710, 730	0.7732	0.6795	0.6760	0.7731	0.0195	0.0234	0.0236	0.0196
SAVI	670, 690, 910	0.9092	0.9404	0.9530	0.9143	0.0275	0.0226	0.0201	0.0271
SR800550	370, 530, 550, 570	0.9474	0.9470	0.8918	0.9385	0.7832	0.7967	1.1244	0.8431
CIIRE	350, 370, 630, 690, 2150	0.8044	0.7743	0.7900	0.7897	0.1737	0.1897	0.1874	0.1805
LCI	350, 370, 390, 590, 610, 630, 650, 690, 710	0.9626	0.9714	0.9720	0.9646	0.0155	0.0139	0.0133	0.0152
ARI	550, 730	0.9508	0.9440	0.8368	0.9074	0.8267	0.8967	1.4874	1.1241
SPRI2	370, 550, 570, 590	0.9404	0.9499	0.8769	0.9344	0.6330	0.5850	0.9103	0.6642
BRI	690, 1370	0.6589	0.5113	0.6306	0.6542	0.0746	0.0902	0.0758	0.0745
SPADI	650, 690, 1090	0.9918	0.9983	0.9977	0.9871	0.0049	0.0022	0.0026	0.0075
GM1	370, 550, 570, 590	0.9452	0.9503	0.8819	0.9370	0.6201	0.5956	0.9115	0.6656
RVI1	370, 690, 910	0.8722	0.8126	0.8359	0.8279	0.2529	0.3012	0.2878	0.2891
CRİ1	390, 430, 870	0.9034	0.9141	0.7998	0.9060	1.0067	0.9405	1.4285	1.0054
RVSI	770, 910	0.9844	0.9873	0.9889	0.9746	0.0084	0.0074	0.0072	0.0111
NDVI1	650, 670, 1090	0.9556	0.9671	0.9760	0.9549	0.0243	0.0210	0.0180	0.0254
NDVI3	350, 370, 690, 710, 2150	0.8602	0.8322	0.8646	0.8551	0.0208	0.0229	0.0209	0.0211
NDVI6	350, 690, 1090	0.9343	0.9583	0.9566	0.9252	0.0222	0.0178	0.0181	0.0241
PSNDB	690, 1090	0.9721	0.9752	0.9856	0.9683	0.0167	0.0157	0.0120	0.0192
LIC2	650, 670, 690, 1090	0.9700	0.9786	0.9846	0.9695	0.0181	0.0153	0.0130	0.0194

Results for 20 nm first dehydration stage

VIs	Inputs	R^2				$RMSE$			
		RF	MLP	SVR	kNN	RF	MLP	SVR	kNN
TM57	1090, 2150, 2170, 2190, 2250, 2270, 2330, 2450	0.9756	0.9849	0.9830	0.9791	0.0271	0.0213	0.0228	0.0271
FWBI	730, 750, 770, 790, 2250,	0.8947	0.9034	0.9123	0.9016	0.0028	0.0028	0.0026	0.0028
LWI	350, 1070, 1470, 1490, 2090	0.9771	0.9945	0.9835	0.9793	0.0293	0.0143	0.0260	0.0290
SIWSI	1030, 1890	0.8034	0.8027	0.8563	0.8185	0.0145	0.0148	0.0128	0.0141
VOG1	370, 710, 1930	0.9436	0.9132	0.9356	0.9271	0.0252	0.0313	0.0267	0.0290
SIPI	350, 910	0.3151	0.4166	0.3642	0.4072	0.0090	0.0083	0.0083	0.0083
VOPT	650, 670, 890	0.9320	0.9604	0.9675	0.9406	0.0904	0.0713	0.0631	0.0867
TCARI	750	0.6325	0.5968	0.7300	0.6940	0.0229	0.0238	0.0193	0.0204
SAVI	650, 670, 890	0.9455	0.9723	0.9795	0.9537	0.0206	0.0159	0.0129	0.0198
SR800550	530, 550, 570, 590, 1310	0.9833	0.9866	0.9369	0.9780	0.3423	0.3126	0.6768	0.4106
CIIRE	690, 710, 1090, 1930	0.9502	0.9538	0.9551	0.9442	0.0736	0.0753	0.0691	0.0796
LCI	430, 550, 690, 710, 1090, 1930	0.9821	0.9951	0.9923	0.9817	0.0126	0.0070	0.0080	0.0133
ARI	550, 1090	0.9567	0.9632	0.8594	0.9504	0.5355	0.5090	0.9686	0.5840
SPRI2	530, 550, 570, 590, 1310	0.9754	0.9751	0.9390	0.9654	0.3020	0.3087	0.4798	0.3655
BRI	350, 1350	0.6564	0.5277	0.6286	0.6195	0.0669	0.0768	0.0676	0.0688
SPADI	450, 470, 490, 610, 630, 650, 670, 690, 1210	0.9914	0.9988	0.9981	0.9918	0.0056	0.0021	0.0027	0.0062
GM1	530, 550, 570, 590, 1310	0.9755	0.9761	0.9389	0.9658	0.3120	0.3137	0.4996	0.3764
RVI1	550, 570, 590, 610, 690, 710, 1090	0.9467	0.9639	0.9498	0.9492	0.1196	0.1073	0.1160	0.1187
CRİ1	390, 410, 890	0.9661	0.9360	0.8677	0.9589	0.4690	0.6817	0.9104	0.5223
RVSI	770, 790, 850	0.9663	0.9738	0.9779	0.9638	0.0057	0.0051	0.0045	0.0060
NDVI1	430, 450, 470, 650, 670, 1190	0.9796	0.9844	0.9865	0.9783	0.0187	0.0168	0.0154	0.0201
NDVI3	550, 710, 1930	0.9472	0.9253	0.9469	0.9440	0.0141	0.0168	0.0141	0.0147
NDVI6	590, 690, 1090	0.9770	0.9811	0.9789	0.9716	0.0140	0.0133	0.0135	0.0166
PSNDB	430, 590, 610, 630, 650, 1190	0.9806	0.9900	0.9903	0.9844	0.0158	0.0117	0.0112	0.0151
LIC2	430, 450, 470, 650, 670, 690, 1190	0.9845	0.9912	0.9911	0.9827	0.0149	0.0116	0.0115	0.0165

Results for 20 nm second dehydration stage

VIs	Inputs	R^2				$RMSE$			
		RF	MLP	SVR	kNN	RF	MLP	SVR	kNN
TM57	430, 450, 470, 650, 670, 690, 1190	0.9487	0.9420	0.9574	0.9495	0.0328	0.0363	0.0300	0.0332
FWBI	730, 750, 770, 790, 810, 2230	0.8647	0.8441	0.8803	0.8608	0.0030	0.0033	0.0028	0.0030
LWI	1470, 2050	0.9583	0.9395	0.9616	0.9609	0.0319	0.0397	0.0314	0.0311
SIWSI	1070, 1890, 1990	0.7219	0.6627	0.7535	0.7112	0.0144	0.0163	0.0137	0.0146
VOG1	710, 1930	0.8885	0.8919	0.9132	0.9037	0.0312	0.0308	0.0278	0.0293
SIPI	690, 890	0.4737	0.3869	0.3346	0.5590	0.0080	0.0084	0.0082	0.0072
VOPT	430, 450, 930	0.9328	0.9474	0.9608	0.9476	0.0964	0.0853	0.0739	0.0939
TCARI	730, 750, 770, 790, 890, 910	0.5623	0.6359	0.6534	0.5749	0.0261	0.0252	0.0243	0.0257
SAVI	430, 670, 1010	0.9365	0.9464	0.9682	0.9460	0.0244	0.0226	0.0174	0.0257
SR800550	350, 370, 570, 590, 610, 630, 1150	0.9870	0.9894	0.9140	0.9768	0.2868	0.2570	0.9033	0.3981
CIIRE	690, 710	0.9375	0.9187	0.9176	0.9300	0.0728	0.0831	0.0830	0.0775
LCI	550, 630, 690, 710	0.9760	0.9722	0.9812	0.9681	0.0152	0.0158	0.0131	0.0178
ARI	350, 550, 570, 1110	0.9657	0.9438	0.8630	0.9508	0.4497	0.5734	0.9775	0.5436
SPRI2	350, 370, 570, 590, 610, 1150	0.9806	0.9822	0.9160	0.9705	0.2531	0.2417	0.6335	0.3236
BRI	350, 1270	0.4154	0.2435	0.4262	0.3375	0.0772	0.0909	0.0740	0.0785
SPADI	450, 470, 490, 590, 630, 650, 670, 690, 1130	0.9844	0.9977	0.9984	0.9818	0.0095	0.0035	0.0029	0.0119
GM1	350, 370, 570, 590, 610, 630, 1150	0.9801	0.9810	0.9141	0.9736	0.2675	0.2638	0.6654	0.3212
RVI1	630, 690, 710	0.9626	0.9566	0.9155	0.9413	0.0931	0.1019	0.1415	0.1166
CRİ1	370, 1110	0.9313	0.8980	0.8395	0.9254	0.7408	0.9112	1.1061	0.7747
RVSI	770, 790, 810, 870	0.9738	0.9745	0.9785	0.9746	0.0047	0.0047	0.0043	0.0047
NDVI1	390, 430, 450, 650, 670, 690, 1150	0.9778	0.9887	0.9846	0.9782	0.0217	0.0156	0.0188	0.0248
NDVI3	550, 710	0.9353	0.9275	0.9481	0.9346	0.0151	0.0160	0.0135	0.0154
NDVI6	690, 1110	0.9756	0.9685	0.9762	0.9564	0.0158	0.0180	0.0156	0.0233
PSNDB	390, 430, 590, 630, 650, 690, 1150	0.9805	0.9914	0.9877	0.9770	0.0179	0.0117	0.0146	0.0219
LIC2	430, 450, 470, 490, 650, 670, 970	0.9808	0.9935	0.9906	0.9834	0.0187	0.0109	0.0138	0.0211

Results for 20 nm third dehydration stage

VIs	Inputs	R^2				$RMSE$			
		RF	MLP	SVR	kNN	RF	MLP	SVR	kNN
TM57	1310, 2250, 2270, 2290, 2310, 2330	0.9526	0.9701	0.9708	0.9556	0.0226	0.0181	0.0177	0.0229
FWBI	730, 750	0.8716	0.8730	0.8899	0.8610	0.0026	0.0027	0.0023	0.0026
LWI	2050, 2470	0.7958	0.7565	0.8283	0.8116	0.0184	0.0214	0.0168	0.0175
SIWSI	750, 770, 830, 850, 870	0.5923	0.5914	0.6801	0.6291	0.0111	0.0112	0.0099	0.0106
VOG1	690, 710, 1950	0.9442	0.9411	0.9379	0.9261	0.0139	0.0147	0.0146	0.0160
SIPI	690, 970	0.5230	0.4230	0.5845	0.5969	0.0114	0.0132	0.0109	0.0107
VOPT	650, 670, 950	0.9483	0.9585	0.9705	0.9520	0.0789	0.0716	0.0595	0.0794
TCARI	790, 810, 830, 850, 870, 1010, 1090, 1110, 1910, 1930, 1950, 1990	0.6022	0.4308	0.5663	0.5692	0.0296	0.0359	0.0307	0.0308
SAVI	650, 670, 970	0.9575	0.9679	0.9775	0.9604	0.0202	0.0177	0.0147	0.0208
SR800550	350, 570, 590, 690, 990	0.9665	0.9744	0.9456	0.9580	0.2945	0.2615	0.3821	0.3326
CIIRE	690, 1950	0.9430	0.9392	0.9367	0.9292	0.0460	0.0496	0.0484	0.0520
LCI	550, 570, 590, 610, 630, 650, 690, 710, 1310	0.9722	0.9829	0.9794	0.9723	0.0156	0.0128	0.0136	0.0161
ARI	350, 410, 510, 530, 550, 570, 990	0.9632	0.9702	0.9103	0.9465	0.2949	0.2766	0.4737	0.3560
SPRI2	350, 590, 630, 690, 990	0.9689	0.9736	0.9432	0.9598	0.2038	0.1902	0.2799	0.2349
BRI	370, 390, 2110	0.6170	0.4946	0.6485	0.6441	0.0626	0.0718	0.0602	0.0604
SPADI	490, 610, 630, 650, 670, 690, 990	0.9807	0.9928	0.9929	0.9788	0.0109	0.0067	0.0066	0.0122
GM1	350, 590, 630, 690, 990	0.9668	0.9701	0.9433	0.9569	0.2211	0.2127	0.2932	0.2543
RVI1	350, 590, 610, 630, 650, 690, 990, 1930	0.9593	0.9572	0.9466	0.9355	0.0655	0.0696	0.0746	0.0825
CRİ1	350, 370, 410, 430, 690, 990	0.9726	0.9813	0.8709	0.9442	0.2697	0.2261	0.5840	0.3854
RVSI	770, 790, 810, 830, 850	0.9887	0.9903	0.9923	0.9881	0.0028	0.0028	0.0023	0.0031
NDVI1	430, 650, 670, 690, 990	0.9732	0.9831	0.9783	0.9696	0.0237	0.0189	0.0214	0.0265
NDVI3	570, 690, 710	0.9495	0.9576	0.9550	0.9566	0.0108	0.0101	0.0104	0.0101
NDVI6	590, 610, 630, 690, 990, 1930	0.9715	0.9774	0.9755	0.9533	0.0164	0.0151	0.0153	0.0215
PSNDB	590, 630, 650, 690, 990	0.9778	0.9889	0.9851	0.9771	0.0185	0.0132	0.0152	0.0202
LIC2	470, 650, 670, 690, 970	0.9772	0.9869	0.9840	0.9745	0.0206	0.0158	0.0173	0.0230

Results for 20 nm fully dry dehydration stage

VIs	Inputs	R^2				$RMSE$			
		RF	MLP	SVR	kNN	RF	MLP	SVR	kNN
TM57	930, 1710, 2250, 2290	0.9966	0.9985	0.9879	0.9942	0.0242	0.0166	0.0462	0.0317
FWBI	750, 830, 1270	0.9935	0.9956	0.9918	0.9943	0.0019	0.0016	0.0022	0.0018
LWI	930, 1570, 1710	0.9968	0.9974	0.9929	0.9968	0.0327	0.0303	0.0488	0.0329
SIWSI	810, 890, 1210, 1390, 2290	0.9963	0.9991	0.9979	0.9957	0.0101	0.0049	0.0076	0.0107
VOG1	610, 850, 1210, 1750	0.9733	0.9703	0.9548	0.9724	0.0499	0.0528	0.0648	0.0506
SIPI	690, 990, 1930	0.7483	0.7062	0.7287	0.7613	0.0082	0.0091	0.0085	0.0080
VOPT	510, 670, 790, 2310	0.9961	0.9994	0.9961	0.9943	0.0526	0.0212	0.0525	0.0633
TCARI	710, 1130, 1170, 1610, 1950	0.8839	0.8461	0.8043	0.8619	0.0229	0.0267	0.0295	0.0249
SAVI	630, 650, 890, 2310	0.9848	0.9843	0.9832	0.9827	0.0175	0.0177	0.0183	0.0187
SR800550	370, 530, 550, 570, 590, 610, 1030, 2310	0.9889	0.9859	0.9022	0.9773	0.3277	0.3728	1.0006	0.4710
CIIRE	570, 790, 1210, 2310	0.9825	0.9746	0.9566	0.9775	0.1322	0.1611	0.2086	0.1497
LCI	590, 610, 630, 690, 710, 1050, 2310	0.9831	0.9898	0.9852	0.9864	0.0156	0.0122	0.0147	0.0141
ARI	350, 370, 530, 550, 730, 750	0.9749	0.9866	0.8974	0.9652	0.5527	0.4065	1.1276	0.6496
SPRI2	530, 550, 570, 590, 630, 890, 910, 1290, 2310	0.9902	0.9963	0.9355	0.9814	0.2708	0.1659	0.7072	0.3740
BRI	350, 870, 1410	0.8876	0.8390	0.8727	0.8871	0.0758	0.0924	0.0806	0.0762
SPADI	630, 650, 910	0.9949	0.9983	0.9966	0.9943	0.0052	0.0030	0.0043	0.0059
GM1	530, 550, 570, 590, 630, 910, 930, 1310	0.9899	0.9951	0.9266	0.9829	0.2706	0.1907	0.7487	0.3554
RVI1	590, 810, 2310	0.9821	0.9751	0.9464	0.9768	0.2248	0.2739	0.3975	0.2553
CRİ1	390, 410, 630, 850, 1270	0.9817	0.9655	0.8603	0.9713	0.6467	0.8969	1.7350	0.8007
RVSI	590, 750, 770, 790, 890, 1210	0.9971	0.9993	0.9978	0.9960	0.0067	0.0034	0.0058	0.0079
NDVI1	490, 610, 630, 650, 690, 930, 2490	0.9856	0.9890	0.9847	0.9840	0.0189	0.0172	0.0196	0.0201
NDVI3	590, 610, 630, 710, 890, 910, 1290, 1710, 2170, 2310	0.9792	0.9979	0.9946	0.9804	0.0167	0.0053	0.0085	0.0161
NDVI6	590, 610, 630, 690, 930, 950, 1330, 2310	0.9828	0.9902	0.9847	0.9847	0.0204	0.0154	0.0192	0.0193
PSNDB	590, 610, 630, 650, 690, 930, 1310, 2470	0.9883	0.9965	0.9935	0.9907	0.0162	0.0089	0.0122	0.0147
LIC2	610, 630, 650, 670, 690, 950, 1470	0.9890	0.9924	0.9890	0.9871	0.0147	0.0122	0.0147	0.0162

Results for 20 nm combined stages

VIs	Inputs	R^2				$RMSE$			
		RF	MLP	SVR	kNN	RF	MLP	SVR	kNN
TM57	710, 800, 2270, 2330	0.9491	0.9604	0.9644	0.9350	0.0425	0.0372	0.0362	0.0513
FWBI	350, 740, 770, 1490	0.8331	0.8572	0.8819	0.8516	0.0009	0.0008	0.0008	0.0008
LWI	350, 1100, 1490, 1520, 2210	0.9637	0.9961	0.9876	0.9663	0.0474	0.0149	0.0275	0.0494
SIWSI	350, 1100, 1490, 1520	0.9425	0.9846	0.9767	0.9435	0.0108	0.0054	0.0067	0.0111
VOG1	530, 560, 710, 1100	0.9404	0.9784	0.9806	0.9575	0.0381	0.0227	0.0215	0.0355
SIPI	350, 740	0.1646	0.3428	0.4670	0.1720	0.0046	0.0043	0.0037	0.0043
VOPT	470, 590, 770,	0.9749	0.9973	0.9959	0.9684	0.0956	0.0281	0.0370	0.1060
TCARI	710, 740	0.6353	0.5532	0.6420	0.6892	0.0244	0.0276	0.0240	0.0224
SAVI	440, 470, 650, 1130	0.9520	0.9878	0.9957	0.9543	0.0169	0.0084	0.0047	0.0179
SR800550	380, 530, 560, 590, 710, 1310	0.9773	0.9895	0.9457	0.9773	0.3510	0.2339	0.5658	0.3853
CIIRE	530, 560, 710, 1100	0.9580	0.9932	0.9804	0.9759	0.1320	0.0544	0.0903	0.1125
LCI	530, 560, 590, 620, 710,1310	0.9507	0.9895	0.9917	0.9552	0.0148	0.0067	0.0060	0.0162
ARI	560, 710, 1100	0.6512	0.6566	0.6990	0.6766	0.3180	0.3252	0.2979	0.3083
SPRI2	380, 530, 560, 590, 710, 1310	0.9775	0.9892	0.9464	0.9747	0.3258	0.2231	0.5238	0.3726
BRI	350, 560, 710, 1310	0.7108	0.7400	0.7838	0.7247	0.0616	0.0594	0.0548	0.0608
SPADI	380, 650, 770	0.9503	0.9943	0.9986	0.9241	0.0097	0.0034	0.0017	0.0130
GM1	380, 530, 560, 590, 710, 1310	0.9781	0.9893	0.9453	0.9752	0.3134	0.2154	0.5114	0.3620
RVI1	530, 560, 710, 1100	0.9661	0.9938	0.9694	0.9801	0.2235	0.0978	0.2171	0.1926
CRİ1	380, 410, 440, 470, 500, 1310	0.9913	0.9824	0.8733	0.9725	0.5340	0.7544	2.2179	0.9592
RVSI	560, 800, 830, 1100	0.9741	0.9758	0.9867	0.9755	0.0077	0.0077	0.0055	0.0080
NDVI1	470, 650, 770	0.9663	0.9940	0.9977	0.9440	0.0149	0.0061	0.0036	0.0211
NDVI3	530, 560, 710, 1130	0.9466	0.9922	0.9937	0.9611	0.0127	0.0048	0.0043	0.0121
NDVI6	530, 560, 590, 620, 1310	0.9545	0.9889	0.9912	0.9462	0.0148	0.0071	0.0063	0.0178
PSNDB	500, 620, 650, 770	0.9680	0.9965	0.9980	0.9512	0.0140	0.0044	0.0033	0.0192
LIC2	470, 650, 770	0.9665	0.9940	0.9976	0.9433	0.0140	0.0057	0.0035	0.0200

Results for 30 nm fresh stage

VIs	Inputs	R^2				$RMSE$			
		RF	MLP	SVR	kNN	RF	MLP	SVR	kNN
TM57	710, 800, 2270, 2330	0.9491	0.9604	0.9644	0.9350	0.0425	0.0372	0.0362	0.0513
FWBI	350, 740, 770, 1490	0.8331	0.8572	0.8819	0.8516	0.0009	0.0008	0.0008	0.0008
LWI	350, 1100, 1490, 1520, 2210	0.9637	0.9961	0.9876	0.9663	0.0474	0.0149	0.0275	0.0494
SIWSI	350, 1100, 1490, 1520	0.9425	0.9846	0.9767	0.9435	0.0108	0.0054	0.0067	0.0111
VOG1	530, 560, 710, 1100	0.9404	0.9784	0.9806	0.9575	0.0381	0.0227	0.0215	0.0355
SIPI	350, 740	0.1646	0.3428	0.4670	0.1720	0.0046	0.0043	0.0037	0.0043
VOPT	470, 590, 770,	0.9749	0.9973	0.9959	0.9684	0.0956	0.0281	0.0370	0.1060
TCARI	710, 740	0.6353	0.5532	0.6420	0.6892	0.0244	0.0276	0.0240	0.0224
SAVI	440, 470, 650, 1130	0.9520	0.9878	0.9957	0.9543	0.0169	0.0084	0.0047	0.0179
SR800550	380, 530, 560, 590, 710, 1310	0.9773	0.9895	0.9457	0.9773	0.3510	0.2339	0.5658	0.3853
CIIRE	530, 560, 710, 1100	0.9580	0.9932	0.9804	0.9759	0.1320	0.0544	0.0903	0.1125
LCI	530, 560, 590, 620, 710,1310	0.9507	0.9895	0.9917	0.9552	0.0148	0.0067	0.0060	0.0162
ARI	560, 710, 1100	0.6512	0.6566	0.6990	0.6766	0.3180	0.3252	0.2979	0.3083
SPRI2	380, 530, 560, 590, 710, 1310	0.9775	0.9892	0.9464	0.9747	0.3258	0.2231	0.5238	0.3726
BRI	350, 560, 710, 1310	0.7108	0.7400	0.7838	0.7247	0.0616	0.0594	0.0548	0.0608
SPADI	380, 650, 770	0.9503	0.9943	0.9986	0.9241	0.0097	0.0034	0.0017	0.0130
GM1	380, 530, 560, 590, 710, 1310	0.9781	0.9893	0.9453	0.9752	0.3134	0.2154	0.5114	0.3620
RVI1	530, 560, 710, 1100	0.9661	0.9938	0.9694	0.9801	0.2235	0.0978	0.2171	0.1926
CRİ1	380, 410, 440, 470, 500, 1310	0.9913	0.9824	0.8733	0.9725	0.5340	0.7544	2.2179	0.9592
RVSI	560, 800, 830, 1100	0.9741	0.9758	0.9867	0.9755	0.0077	0.0077	0.0055	0.0080
NDVI1	470, 650, 770	0.9663	0.9940	0.9977	0.9440	0.0149	0.0061	0.0036	0.0211
NDVI3	530, 560, 710, 1130	0.9466	0.9922	0.9937	0.9611	0.0127	0.0048	0.0043	0.0121
NDVI6	530, 560, 590, 620, 1310	0.9545	0.9889	0.9912	0.9462	0.0148	0.0071	0.0063	0.0178
PSNDB	500, 620, 650, 770	0.9680	0.9965	0.9980	0.9512	0.0140	0.0044	0.0033	0.0192
LIC2	470, 650, 770	0.9665	0.9940	0.9976	0.9433	0.0140	0.0057	0.0035	0.0200

Results for 30 nm first dehydration stage

VIs	Inputs	R^2				$RMSE$			
		RF	MLP	SVR	kNN	RF	MLP	SVR	kNN
TM57	710, 800, 2270, 2330	0.9491	0.9604	0.9644	0.9350	0.0425	0.0372	0.0362	0.0513
FWBI	350, 740, 770, 1490	0.8331	0.8572	0.8819	0.8516	0.0009	0.0008	0.0008	0.0008
LWI	350, 1100, 1490, 1520, 2210	0.9637	0.9961	0.9876	0.9663	0.0474	0.0149	0.0275	0.0494
SIWSI	350, 1100, 1490, 1520	0.9425	0.9846	0.9767	0.9435	0.0108	0.0054	0.0067	0.0111
VOG1	530, 560, 710, 1100	0.9404	0.9784	0.9806	0.9575	0.0381	0.0227	0.0215	0.0355
SIPI	350, 740	0.1646	0.3428	0.4670	0.1720	0.0046	0.0043	0.0037	0.0043
VOPT	470, 590, 770,	0.9749	0.9973	0.9959	0.9684	0.0956	0.0281	0.0370	0.1060
TCARI	710, 740	0.6353	0.5532	0.6420	0.6892	0.0244	0.0276	0.0240	0.0224
SAVI	440, 470, 650, 1130	0.9520	0.9878	0.9957	0.9543	0.0169	0.0084	0.0047	0.0179
SR800550	380, 530, 560, 590, 710, 1310	0.9773	0.9895	0.9457	0.9773	0.3510	0.2339	0.5658	0.3853
CIIRE	530, 560, 710, 1100	0.9580	0.9932	0.9804	0.9759	0.1320	0.0544	0.0903	0.1125
LCI	530, 560, 590, 620, 710,1310	0.9507	0.9895	0.9917	0.9552	0.0148	0.0067	0.0060	0.0162
ARI	560, 710, 1100	0.6512	0.6566	0.6990	0.6766	0.3180	0.3252	0.2979	0.3083
SPRI2	380, 530, 560, 590, 710, 1310	0.9775	0.9892	0.9464	0.9747	0.3258	0.2231	0.5238	0.3726
BRI	350, 560, 710, 1310	0.7108	0.7400	0.7838	0.7247	0.0616	0.0594	0.0548	0.0608
SPADI	380, 650, 770	0.9503	0.9943	0.9986	0.9241	0.0097	0.0034	0.0017	0.0130
GM1	380, 530, 560, 590, 710, 1310	0.9781	0.9893	0.9453	0.9752	0.3134	0.2154	0.5114	0.3620
RVI1	530, 560, 710, 1100	0.9661	0.9938	0.9694	0.9801	0.2235	0.0978	0.2171	0.1926
CRİ1	380, 410, 440, 470, 500, 1310	0.9913	0.9824	0.8733	0.9725	0.5340	0.7544	2.2179	0.9592
RVSI	560, 800, 830, 1100	0.9741	0.9758	0.9867	0.9755	0.0077	0.0077	0.0055	0.0080
NDVI1	470, 650, 770	0.9663	0.9940	0.9977	0.9440	0.0149	0.0061	0.0036	0.0211
NDVI3	530, 560, 710, 1130	0.9466	0.9922	0.9937	0.9611	0.0127	0.0048	0.0043	0.0121
NDVI6	530, 560, 590, 620, 1310	0.9545	0.9889	0.9912	0.9462	0.0148	0.0071	0.0063	0.0178
PSNDB	500, 620, 650, 770	0.9680	0.9965	0.9980	0.9512	0.0140	0.0044	0.0033	0.0192
LIC2	470, 650, 770	0.9665	0.9940	0.9976	0.9433	0.0140	0.0057	0.0035	0.0200

Results for 30 nm second dehydration stage

VIs	Inputs	R^2				$RMSE$			
		RF	MLP	SVR	kNN	RF	MLP	SVR	kNN
TM57	2180, 2270, 2330	0.9475	0.9403	0.9575	0.9432	0.0332	0.0366	0.0300	0.0349
FWBI	740, 770, 800, 2240	0.8651	0.8426	0.8829	0.8573	0.0030	0.0034	0.0028	0.0030
LWI	1460, 2060	0.9565	0.9382	0.9619	0.9627	0.0326	0.0401	0.0313	0.0304
SIWSI	380, 1070, 1100, 1460, 1880, 1970, 2000	0.7491	0.7039	0.7704	0.7192	0.0138	0.0152	0.0133	0.0145
VOG1	710, 1940	0.8896	0.8919	0.9133	0.9042	0.0311	0.0308	0.0278	0.0292
SIPI	620, 650, 710	0.5168	0.5668	0.3189	0.4839	0.0077	0.0080	0.0083	0.0077
VOPT	440, 680, 920	0.9387	0.9544	0.9664	0.9564	0.0926	0.0795	0.0682	0.0876
TCARI	740, 770, 890	0.5598	0.6009	0.6563	0.5657	0.0263	0.0260	0.0243	0.0260
SAVI	440, 680, 1010	0.9365	0.9467	0.9685	0.9455	0.0245	0.0225	0.0173	0.0259
SR800550	350, 380, 530, 560, 590, 620, 1010	0.9867	0.9906	0.9115	0.9796	0.2899	0.2443	0.9074	0.3779
CIIRE	620, 710	0.9239	0.9100	0.9187	0.9181	0.0800	0.0873	0.0826	0.0834
LCI	440, 560, 620, 710	0.9707	0.9664	0.9797	0.9689	0.0167	0.0174	0.0137	0.0177
ARI	350, 560, 590, 1100	0.9670	0.9417	0.8600	0.9481	0.4407	0.5836	0.9858	0.5573
SPRI2	350, 380, 590, 620, 650,1160	0.9831	0.9870	0.9124	0.9703	0.2366	0.2068	0.6566	0.3253
BRI	350, 1280	0.4292	0.2437	0.4254	0.3698	0.0763	0.0909	0.0740	0.0768
SPADI	440,470, 500, 590, 620, 650, 680, 1130	0.9841	0.9979	0.9984	0.9811	0.0095	0.0033	0.0028	0.0125
GM1	350, 380, 590, 620, 650, 1160	0.9833	0.9866	0.9082	0.9709	0.2460	0.2199	0.6846	0.3378
RVI1	560, 620, 710	0.9407	0.9530	0.9141	0.9194	0.1162	0.1047	0.1391	0.1350
CRİ1	350, 380,410, 1010	0.9727	0.9678	0.8270	0.9527	0.4751	0.5166	1.2050	0.6225
RVSI	770, 800, 860	0.9757	0.9749	0.9785	0.9736	0.0046	0.0046	0.0043	0.0048
NDVI1	410, 440, 470, 650, 680, 1160	0.9776	0.9885	0.9847	0.9768	0.0218	0.0157	0.0187	0.0254
NDVI3	560, 710	0.9342	0.9282	0.9484	0.9329	0.0152	0.0159	0.0135	0.0156
NDVI6	620, 1100	0.9675	0.9579	0.9721	0.9518	0.0181	0.0207	0.0167	0.0243
PSNDB	410, 440, 590, 620, 650, 680, 1160	0.9800	0.9913	0.9868	0.9788	0.0182	0.0117	0.0153	0.0217
LIC2	410, 440, 470, 500, 650, 680, 950	0.9809	0.9946	0.9905	0.9824	0.0188	0.0101	0.0140	0.0215

Results for 30 nm third dehydration stage

VIs	Inputs	R^2				$RMSE$			
		RF	MLP	SVR	kNN	RF	MLP	SVR	kNN
TM57	1310, 2270, 2300, 2330	0.9558	0.9704	0.9633	0.9497	0.0219	0.0180	0.0199	0.0245
FWBI	740	0.8553	0.8758	0.8914	0.8669	0.0027	0.0027	0.0023	0.0025
LWI	2060, 2450	0.8160	0.7487	0.8249	0.8062	0.0175	0.0217	0.0170	0.0177
SIWSI	770, 830, 860	0.5601	0.5759	0.6771	0.6261	0.0114	0.0114	0.0099	0.0107
VOG1	590, 710, 1940	0.9432	0.9300	0.9312	0.9181	0.0140	0.0159	0.0154	0.0168
SIPI	620, 980	0.5027	0.3888	0.5611	0.5681	0.0117	0.0135	0.0111	0.0110
VOPT	650, 680, 950	0.9452	0.9555	0.9700	0.9499	0.0810	0.0743	0.0600	0.0812
TCARI	800, 830, 860, 890, 1010, 1910, 1940, 2000	0.5681	0.4676	0.5667	0.5687	0.0306	0.0349	0.0307	0.0309
SAVI	470, 680, 980	0.9530	0.9600	0.9757	0.9545	0.0211	0.0198	0.0152	0.0219
SR800550	350, 560, 590, 620, 980	0.9722	0.9794	0.9447	0.9609	0.2687	0.2357	0.3851	0.3212
CIIRE	350, 560, 590, 620, 650, 680, 710, 1940	0.9442	0.9339	0.9301	0.9257	0.0454	0.0504	0.0508	0.0525
LCI	560, 590, 620, 650, 710	0.9711	0.9730	0.9684	0.9691	0.0160	0.0155	0.0172	0.0167
ARI	350, 380, 410, 500, 530, 560, 590, 1130	0.9631	0.9737	0.9107	0.9528	0.2953	0.2586	0.4684	0.3346
SPRI2	350, 590, 620, 650, 980	0.9744	0.9818	0.9424	0.9626	0.1852	0.1584	0.2819	0.2269
BRI	350, 380, 2240	0.6491	0.5308	0.6455	0.6388	0.0601	0.0708	0.0605	0.0614
SPADI	650, 680, 980	0.9804	0.9928	0.9929	0.9773	0.0110	0.0067	0.0066	0.0133
GM1	350, 590, 620, 650, 980	0.9744	0.9810	0.9415	0.9607	0.1947	0.1696	0.2990	0.2435
RVI1	350, 560, 590, 620, 650, 680, 980, 1940	0.9526	0.9418	0.9395	0.9338	0.0704	0.0803	0.0793	0.0833
CRİ1	350, 380, 410, 620, 680, 980	0.9697	0.9825	0.8718	0.9475	0.2844	0.2187	0.5797	0.3752
RVSI	800, 830	0.9870	0.9887	0.9923	0.9860	0.0030	0.0030	0.0023	0.0033
NDVI1	470, 620, 650, 680, 980	0.9734	0.9842	0.9782	0.9715	0.0236	0.0183	0.0215	0.0258
NDVI3	560, 710	0.9436	0.9422	0.9479	0.9477	0.0114	0.0118	0.0110	0.0110
NDVI6	560, 590, 620, 650, 980, 1940	0.9663	0.9673	0.9702	0.9510	0.0178	0.0179	0.0168	0.0221
PSNDB	590, 620, 650, 680, 980	0.9771	0.9873	0.9836	0.9786	0.0188	0.0141	0.0160	0.0195
LIC2	470, 620, 650, 680, 980	0.9762	0.9875	0.9839	0.9752	0.0210	0.0154	0.0174	0.0229

Results for 30 nm fully dry stage

VIs	Inputs	R^2				$RMSE$			
		RF	MLP	SVR	kNN	RF	MLP	SVR	kNN
TM57	920, 1700, 2270, 2300	0.9967	0.9979	0.9872	0.9943	0.0241	0.0192	0.0475	0.0315
FWBI	770, 800, 830, 1250, 1280	0.9939	0.9958	0.9917	0.9939	0.0018	0.0015	0.0022	0.0018
LWI	920, 1580, 1700	0.9963	0.9965	0.9932	0.9965	0.0351	0.0348	0.0477	0.0344
SIWSI	800, 890, 1310, 1850, 2300	0.9972	0.9990	0.9978	0.9962	0.0088	0.0053	0.0078	0.0101
VOG1	620, 860, 1220, 1760	0.9717	0.9696	0.9526	0.9713	0.0513	0.0534	0.0665	0.0517
SIPI	590, 650, 680, 980, 1910, 1940	0.7573	0.6933	0.7244	0.7559	0.0080	0.0092	0.0086	0.0081
VOPT	500, 680, 800, 2300	0.9964	0.9994	0.9958	0.9947	0.0509	0.0201	0.0546	0.0609
TCARI	710, 1130, 1160, 1610, 1940	0.8857	0.8436	0.8054	0.8646	0.0227	0.0271	0.0295	0.0247
SAVI	620, 650, 890, 2300	0.9847	0.9849	0.9834	0.9827	0.0176	0.0174	0.0182	0.0188
SR800550	380, 530, 560, 590, 620, 1040, 2300	0.9884	0.9861	0.9034	0.9767	0.3350	0.3689	0.9929	0.4788
CIIRE	590, 800, 1190, 2300,	0.9789	0.9704	0.9553	0.9736	0.1451	0.1736	0.2119	0.1619
LCI	560, 590, 620, 650, 710, 920, 2300	0.9860	0.9967	0.9940	0.9899	0.0143	0.0070	0.0094	0.0122
ARI	530,560, 740	0.9747	0.9484	0.8535	0.9661	0.5547	0.7935	1.3036	0.6433
SPRI2	380, 530, 560, 590, 620, 890, 920, 1310	0.9906	0.9969	0.9329	0.9819	0.2651	0.1553	0.7226	0.3664
BRI	350, 770, 980, 1400	0.8823	0.8461	0.8705	0.8792	0.0775	0.0908	0.0811	0.0788
SPADI	620, 650, 920	0.9952	0.9984	0.9967	0.9944	0.0050	0.0029	0.0043	0.0059
GM1	380, 530, 560, 590, 620, 890, 920, 1310	0.9904	0.9964	0.9314	0.9815	0.2650	0.1708	0.7291	0.3672
RVI1	590, 830, 2300	0.9821	0.9736	0.9422	0.9770	0.2253	0.2820	0.4111	0.2547
CRİ1	380, 410, 440, 620, 860, 890, 1250	0.9799	0.9875	0.8586	0.9703	0.6768	0.5417	1.7526	0.8150
RVSI	740, 770, 800, 2300	0.9980	0.9998	0.9995	0.9972	0.0056	0.0017	0.0029	0.0067
NDVI1	500, 590, 620, 650, 680, 920, 2300	0.9874	0.9918	0.9856	0.9857	0.0177	0.0144	0.0190	0.0190
NDVI3	590, 620, 650, 710, 890, 920, 1310, 1700, 2180, 2300	0.9792	0.9984	0.9952	0.9804	0.0167	0.0046	0.0080	0.0161
NDVI6	500, 590, 620, 650, 920, 950, 1340, 2300	0.9831	0.9918	0.9876	0.9829	0.0202	0.0141	0.0173	0.0204
PSNDB	500, 590, 620, 650, 680, 920, 1310, 2480	0.9894	0.9968	0.9935	0.9902	0.0154	0.0085	0.0122	0.0150
LIC2	500, 590, 620, 650, 680, 950	0.9894	0.9915	0.9889	0.9868	0.0144	0.0129	0.0148	0.0162

Results for 30 nm combined stages

VIs	Inputs	R^2				$RMSE$			
		RF	MLP	SVR	kNN	RF	MLP	SVR	kNN
TM57	710, 1110, 2150, 2190, 2230, 2270, 2310, 2350, 2390	0.9493	0.9789	0.9665	0.9388	0.0423	0.0272	0.0356	0.0482
FWBI	350, 750, 790, 1510	0.8355	0.8554	0.8713	0.8310	0.0009	0.0008	0.0008	0.0009
LWI	350, 1110, 1470, 1510, 2190	0.9626	0.9972	0.9876	0.9624	0.0483	0.0127	0.0275	0.0509
SIWSI	350, 1110, 1510, 2190	0.9302	0.9334	0.9671	0.9317	0.0118	0.0112	0.0079	0.0119
VOG1	550, 710, 1110	0.9519	0.9809	0.9808	0.9593	0.0351	0.0216	0.0214	0.0348
SIPI	350, 1150	0.3200	0.3035	0.4744	0.2696	0.0042	0.0045	0.0037	0.0041
VOPT	470, 670, 790	0.9656	0.9990	0.9961	0.9693	0.1044	0.0177	0.0380	0.1038
TCARI	710, 750	0.6254	0.5171	0.6167	0.6553	0.0246	0.0298	0.0248	0.0234
SAVI	430, 670, 1150, 1990	0.9415	0.9886	0.9947	0.9430	0.0182	0.0080	0.0053	0.0203
SR800550	390, 550, 590, 710, 1310	0.9770	0.9887	0.9413	0.9757	0.3527	0.2434	0.5755	0.3986
CIIRE	550, 710, 1110	0.9692	0.9931	0.9794	0.9769	0.1158	0.0552	0.0925	0.1103
LCI	510, 550, 590, 710, 1110	0.9552	0.9978	0.9977	0.9638	0.0143	0.0032	0.0031	0.0145
ARI	550, 710, 1110	0.6386	0.6570	0.6992	0.6866	0.3229	0.3245	0.2977	0.3039
SPRI2	390, 550, 590, 710, 1310	0.9764	0.9878	0.9430	0.9731	0.3312	0.2377	0.5253	0.3835
BRI	350, 550, 710, 1310	0.7032	0.7372	0.7780	0.7188	0.0623	0.0596	0.0553	0.0613
SPADI	470, 630, 670, 790	0.9583	0.9931	0.9983	0.9431	0.0094	0.0038	0.0019	0.0122
GM1	390, 550, 590, 710, 1310	0.9751	0.9882	0.9409	0.9736	0.3294	0.2268	0.5206	0.3734
RVI1	550, 590, 710, 1110	0.9647	0.9951	0.9685	0.9797	0.2282	0.0865	0.2211	0.1922
CRİ1	390, 430, 470, 510, 1310	0.9900	0.9840	0.8738	0.9718	0.5734	0.7271	2.1880	0.9747
RVSI	550, 790, 830, 1110	0.9753	0.9777	0.9871	0.9746	0.0075	0.0073	0.0054	0.0081
NDVI1	390, 430, 470, 510, 630, 670, 1310, 1950	0.9597	0.9941	0.9950	0.9482	0.0159	0.0061	0.0053	0.0198
NDVI3	550, 710, 1110	0.9587	0.9918	0.9940	0.9638	0.0116	0.0050	0.0043	0.0118
NDVI6	510, 550, 590, 630, 710, 1310	0.9544	0.9913	0.9930	0.9515	0.0150	0.0063	0.0056	0.0172
PSNDB	470, 630, 790	0.9683	0.9967	0.9981	0.9509	0.0140	0.0044	0.0031	0.0192
LIC2	390, 430, 470, 510, 630, 670, 1310, 1950	0.9616	0.9936	0.9959	0.9490	0.0147	0.0060	0.0046	0.0187

Results for 40 nm fresh stage

VIs	Inputs	R^2				$RMSE$			
		RF	MLP	SVR	kNN	RF	MLP	SVR	kNN
TM57	350, 2190, 2270, 2310, 2350, 2390, 2430	0.9598	0.9480	0.9476	0.9629	0.0345	0.0403	0.0393	0.0342
FWBI	350, 750	0.9352	0.9194	0.9476	0.9210	0.0030	0.0034	0.0027	0.0034
LWI	350, 1470	0.9138	0.8787	0.9238	0.9121	0.0573	0.0734	0.0541	0.0581
SIWSI	870, 1990	0.8301	0.8276	0.8663	0.8455	0.0192	0.0192	0.0173	0.0189
VOG1	350, 630, 1990, 2150	0.7332	0.6876	0.7336	0.6918	0.0581	0.0640	0.0598	0.0617
SIPI	630, 670, 1350	0.5896	0.3720	0.4293	0.5274	0.0051	0.0078	0.0056	0.0054
VOPT	670, 830	0.9799	0.9912	0.9887	0.9786	0.0789	0.0460	0.0569	0.0800
TCARI	710, 750	0.7847	0.6914	0.6601	0.7208	0.0190	0.0227	0.0241	0.0214
SAVI	630, 670, 910	0.9043	0.9431	0.9537	0.9131	0.0282	0.0221	0.0199	0.0272
SR800550	350, 510, 550, 590	0.9544	0.9624	0.8895	0.9557	0.7284	0.6730	1.1350	0.7232
CIIRE	350, 390, 590, 630, 2150	0.7717	0.7392	0.7759	0.7583	0.1859	0.2027	0.1932	0.1913
LCI	350, 390, 510, 590, 630, 670, 710	0.9580	0.9618	0.9676	0.9605	0.0163	0.0158	0.0144	0.0159
ARI	550, 750	0.9551	0.9398	0.8467	0.9133	0.7909	0.9229	1.4548	1.0956
SPRI2	350, 510, 550, 590, 630	0.9469	0.9510	0.8763	0.9457	0.5968	0.5852	0.9164	0.6056
BRI	670, 1350	0.6388	0.4780	0.6002	0.6140	0.0759	0.0930	0.0781	0.0779
SPADI	630, 670, 1110	0.9916	0.9980	0.9973	0.9878	0.0050	0.0024	0.0028	0.0075
GM1	390, 550, 590	0.9452	0.9483	0.8769	0.9381	0.6195	0.6078	0.9316	0.6609
RVI1	350, 390, 630, 670, 910	0.8310	0.8469	0.8311	0.7937	0.2848	0.2723	0.2917	0.3126
CRI1	390, 430, 870	0.9034	0.9141	0.7998	0.9060	1.0067	0.9405	1.4285	1.0054
RVSI	750, 790	0.9864	0.9864	0.9869	0.9808	0.0078	0.0076	0.0077	0.0095
NDVI1	670, 1070	0.9571	0.9655	0.9766	0.9542	0.0240	0.0215	0.0178	0.0258
NDVI3	350, 630, 710	0.8508	0.8071	0.8619	0.8546	0.0214	0.0245	0.0210	0.0212
NDVI6	630, 1070	0.9382	0.9288	0.9457	0.9315	0.0214	0.0231	0.0206	0.0231
PSNDB	630, 670, 1110	0.9690	0.9793	0.9824	0.9652	0.0176	0.0144	0.0133	0.0199
LIC2	670, 1110	0.9697	0.9753	0.9844	0.9669	0.0182	0.0164	0.0131	0.0205

Results for 40 nm first dehydration stage

VIs	Inputs	R^2				$RMSE$			
		RF	MLP	SVR	kNN	RF	MLP	SVR	kNN
TM57	590, 2270	0.9587	0.9521	0.9617	0.9523	0.0349	0.0385	0.0337	0.0388
FWBI	750, 2190	0.8864	0.9024	0.9108	0.9028	0.0030	0.0028	0.0026	0.0028
LWI	350, 1070, 1470, 1510, 2070	0.9751	0.9976	0.9886	0.9787	0.0303	0.0095	0.0221	0.0292
SIWSI	1070, 1470	0.8081	0.8040	0.8657	0.8290	0.0144	0.0148	0.0124	0.0137
VOG1	470, 710, 1950	0.9374	0.9098	0.9329	0.9357	0.0263	0.0316	0.0272	0.0270
SIPI	350, 910	0.3151	0.4166	0.3642	0.4072	0.0090	0.0083	0.0083	0.0083
VOPT	470, 670, 910	0.9201	0.9403	0.9528	0.9247	0.0970	0.0866	0.0759	0.0967
TCARI	750	0.6325	0.5968	0.7300	0.6940	0.0229	0.0238	0.0193	0.0204
SAVI	470, 670, 910	0.9388	0.9590	0.9707	0.9449	0.0217	0.0188	0.0155	0.0214
SR800550	510, 550, 590, 630, 1310	0.9853	0.9896	0.9384	0.9839	0.3222	0.2750	0.6660	0.3574
CIIRE	630, 710, 1110	0.9428	0.9423	0.9554	0.9410	0.0786	0.0825	0.0690	0.0807
LCI	430, 550, 630, 710, 1110, 1950	0.9812	0.9937	0.9919	0.9790	0.0127	0.0081	0.0081	0.0141
ARI	550, 1110	0.9563	0.9632	0.8598	0.9520	0.5380	0.5091	0.9671	0.5800
SPRI2	390, 550, 590, 630, 1310	0.9766	0.9779	0.9376	0.9727	0.2943	0.2894	0.4811	0.3261
BRI	350, 1350	0.6564	0.5277	0.6286	0.6195	0.0669	0.0768	0.0676	0.0688
SPADI	430, 470, 510, 590, 630, 670, 1190	0.9924	0.9986	0.9980	0.9919	0.0053	0.0023	0.0028	0.0064
GM1	390, 550, 590, 630, 1310	0.9762	0.9793	0.9379	0.9739	0.3076	0.2907	0.4996	0.3310
RVI1	550, 590, 630, 710, 1110	0.9400	0.9626	0.9494	0.9418	0.1265	0.1115	0.1164	0.1261
CRİ1	390, 430, 1310	0.9682	0.9448	0.8684	0.9507	0.4564	0.6241	0.9118	0.5676
RVSI	790, 870	0.9653	0.9706	0.9780	0.9624	0.0058	0.0055	0.0045	0.0062
NDVI1	430, 470, 510, 630, 670, 1190	0.9794	0.9879	0.9867	0.9761	0.0188	0.0149	0.0153	0.0209
NDVI3	550, 710, 1950	0.9466	0.9254	0.9468	0.9432	0.0141	0.0168	0.0141	0.0148
NDVI6	510, 550, 590, 630, 670, 1230	0.9700	0.9772	0.9763	0.9676	0.0160	0.0150	0.0142	0.0172
PSNDB	430, 510, 590, 630, 670, 1190	0.9799	0.9895	0.9897	0.9836	0.0160	0.0120	0.0115	0.0154
LIC2	430, 470, 510, 630, 670, 1190	0.9836	0.9923	0.9910	0.9815	0.0153	0.0109	0.0115	0.0171

Results for 40 nm second dehydration stage

VIs	Inputs	R^2				$RMSE$			
		RF	MLP	SVR	kNN	RF	MLP	SVR	kNN
TM57	2190, 2270, 2350	0.9503	0.9420	0.9576	0.9458	0.0323	0.0363	0.0300	0.0343
FWBI	750, 790, 2030	0.8552	0.8542	0.8869	0.8614	0.0031	0.0032	0.0027	0.0030
LWI	1430, 1470, 2070	0.9625	0.9469	0.9606	0.9613	0.0304	0.0375	0.0315	0.0312
SIWSI	1070, 1470, 1990	0.7230	0.6982	0.7825	0.7175	0.0144	0.0153	0.0130	0.0145
VOG1	710, 1950	0.8887	0.8920	0.9131	0.9024	0.0312	0.0308	0.0278	0.0296
SIPI	630, 710,	0.4886	0.4169	0.3398	0.5454	0.0080	0.0084	0.0082	0.0074
VOPT	430, 670, 950	0.9328	0.9404	0.9577	0.9399	0.0963	0.0903	0.0766	0.0989
TCARI	750, 790, 910	0.5432	0.6111	0.6541	0.5534	0.0266	0.0256	0.0243	0.0262
SAVI	430, 670, 1150	0.9330	0.9395	0.9649	0.9379	0.0250	0.0239	0.0182	0.0272
SR800550	350, 390, 550, 590, 630, 1150	0.9871	0.9910	0.9101	0.9728	0.2838	0.2370	0.9168	0.4270
CIIRE	630, 710	0.9253	0.9107	0.9192	0.9189	0.0793	0.0870	0.0824	0.0830
LCI	430, 550, 630, 710	0.9710	0.9680	0.9799	0.9687	0.0166	0.0170	0.0137	0.0177
ARI	350, 550, 590, 1110	0.9633	0.9433	0.8598	0.9500	0.4643	0.5761	0.9845	0.5484
SPRI2	350, 390, 550, 590, 630, 1150	0.9819	0.9851	0.9145	0.9695	0.2441	0.2215	0.6427	0.3268
BRI	350,1270	0.4154	0.2435	0.4262	0.3375	0.0772	0.0909	0.0740	0.0785
SPADI	430, 470, 510, 550, 590, 630, 670, 1110	0.9838	0.9983	0.9985	0.9797	0.0097	0.0030	0.0028	0.0127
GM1	350, 390, 550, 590, 630, 1150	0.9819	0.9848	0.9126	0.9695	0.2560	0.2355	0.6634	0.3425
RVI1	590, 630, 710	0.9381	0.9428	0.9132	0.9242	0.1184	0.1156	0.1414	0.1316
CRI1	350, 390, 430, 1030	0.9800	0.9772	0.8247	0.9595	0.4060	0.4373	1.2662	0.5799
RVSI	790, 910	0.9757	0.9754	0.9799	0.9736	0.0046	0.0048	0.0042	0.0049
NDVI1	390, 430, 470, 630, 670, 1150	0.9783	0.9888	0.9844	0.9776	0.0215	0.0154	0.0188	0.0252
NDVI3	550, 710	0.9353	0.9275	0.9481	0.9346	0.0151	0.0160	0.0135	0.0154
NDVI6	630, 1110	0.9676	0.9589	0.9717	0.9546	0.0180	0.0204	0.0168	0.0240
PSNDB	390, 430, 590, 630, 670, 1150	0.9796	0.9900	0.9880	0.9771	0.0182	0.0126	0.0142	0.0217
LIC2	390, 430, 470, 510, 630, 670, 990	0.9809	0.9948	0.9898	0.9810	0.0188	0.0099	0.0142	0.0220

Results for 40 nm third dehydration stage

VIs	Inputs	R^2				$RMSE$			
		RF	MLP	SVR	kNN	RF	MLP	SVR	kNN
TM57	1310, 2270	0.9565	0.9620	0.9667	0.9507	0.0217	0.0205	0.0189	0.0254
FWBI	750	0.8559	0.8714	0.8846	0.8584	0.0027	0.0027	0.0024	0.0026
LWI	350, 2030, 2070, 2110, 2150, 2470	0.7991	0.7787	0.8177	0.8177	0.0181	0.0204	0.0172	0.0173
SIWSI	750, 830, 870	0.5975	0.5774	0.6793	0.6313	0.0110	0.0114	0.0099	0.0106
VOG1	590, 630, 710, 1950	0.9345	0.9337	0.9335	0.9307	0.0150	0.0156	0.0152	0.0156
SIPI	630, 990	0.4670	0.3956	0.5620	0.5449	0.0121	0.0135	0.0111	0.0112
VOPT	470, 670, 950	0.9505	0.9614	0.9703	0.9515	0.0772	0.0695	0.0597	0.0786
TCARI	790, 830, 870, 910, 1110, 1910, 1950, 1990	0.5745	0.5143	0.5657	0.5455	0.0305	0.0327	0.0307	0.0315
SAVI	470, 670, 990	0.9539	0.9608	0.9754	0.9521	0.0209	0.0196	0.0153	0.0222
SR800550	350, 550, 590, 630, 990	0.9700	0.9796	0.9448	0.9610	0.2786	0.2341	0.3851	0.3214
CIIRE	590, 630, 710, 1950	0.9464	0.9332	0.9301	0.9323	0.0446	0.0510	0.0508	0.0506
LCI	590, 630, 670, 710	0.9718	0.9736	0.9685	0.9678	0.0158	0.0153	0.0173	0.0171
ARI	350, 390, 430, 510, 550, 590, 990	0.9654	0.9719	0.9097	0.9392	0.2862	0.2674	0.4748	0.3787
SPRI2	350, 590, 630, 990	0.9667	0.9726	0.9438	0.9583	0.2108	0.1928	0.2777	0.2384
BRI	350, 390, 2110	0.6407	0.5107	0.6443	0.6258	0.0608	0.0706	0.0605	0.0624
SPADI	470, 510, 590, 630, 670, 990	0.9791	0.9921	0.9928	0.9771	0.0113	0.0069	0.0067	0.0128
GM1	350, 590, 630, 990	0.9641	0.9692	0.9425	0.9562	0.2298	0.2147	0.2943	0.2559
RVI1	350, 550, 590, 630, 670, 990, 1910	0.9534	0.9426	0.9417	0.9265	0.0699	0.0822	0.0779	0.0872
CRI1	350, 390, 430, 590, 630, 990	0.9721	0.9811	0.8711	0.9415	0.2723	0.2276	0.5798	0.3944
RVSI	790, 830	0.9870	0.9889	0.9922	0.9869	0.0030	0.0030	0.0023	0.0032
NDVI1	470, 590, 630, 670, 990	0.9737	0.9853	0.9769	0.9672	0.0235	0.0176	0.0222	0.0275
NDVI3	590, 710	0.9425	0.9460	0.9500	0.9475	0.0115	0.0113	0.0109	0.0111
NDVI6	550, 590, 630, 990, 1950	0.9653	0.9706	0.9720	0.9481	0.0181	0.0170	0.0162	0.0229
PSNDB	590, 630, 670, 990	0.9762	0.9868	0.9837	0.9744	0.0192	0.0143	0.0159	0.0214
LIC2	470, 590, 630, 670, 990	0.9759	0.9881	0.9831	0.9714	0.0212	0.0149	0.0178	0.0244

Results for 40 nm fully dry stage

VIs	Inputs	R^2				$RMSE$			
		RF	MLP	SVR	kNN	RF	MLP	SVR	kNN
TM57	910,1710, 2270, 2310,	0.9967	0.9979	0.9869	0.9941	0.0241	0.0193	0.0479	0.0320
FWBI	750, 790, 830, 1150, 1270	0.9940	0.9961	0.9925	0.9940	0.0018	0.0015	0.0021	0.0018
LWI	910, 1590, 1710	0.9959	0.9954	0.9931	0.9961	0.0372	0.0397	0.0478	0.0359
SIWSI	790, 870, 910, 1310, 1390, 1710, 2310	0.9971	0.9998	0.9990	0.9969	0.0090	0.0023	0.0053	0.0091
VOG1	630, 870, 1190, 1750	0.9721	0.9693	0.9525	0.9706	0.0510	0.0537	0.0666	0.0523
SIPI	550, 590, 630, 990,1910, 1950	0.7522	0.7487	0.7203	0.7388	0.0081	0.0089	0.0086	0.0083
VOPT	510, 670, 790, 2310	0.9961	0.9994	0.9961	0.9943	0.0526	0.0212	0.0525	0.0633
TCARI	710, 1150, 1190, 1790, 1950	0.8849	0.8413	0.8021	0.8639	0.0228	0.0270	0.0297	0.0248
SAVI	470, 630, 870, 2310	0.9892	0.9890	0.9902	0.9879	0.0149	0.0148	0.0140	0.0159
SR800550	350, 510, 550, 590, 630, 1030, 2310	0.9878	0.9866	0.9037	0.9741	0.3443	0.3609	0.9920	0.5028
CIIRE	510, 590, 790, 870, 1190, 1710, 2310	0.9785	0.9849	0.9650	0.9715	0.1467	0.1236	0.1902	0.1682
LCI	510, 590, 630, 670, 710, 910, 2310	0.9858	0.9963	0.9936	0.9892	0.0144	0.0073	0.0097	0.0125
ARI	350, 390, 550, 750	0.9741	0.9496	0.8804	0.9688	0.5605	0.7822	1.1861	0.6161
SPRI2	510, 550, 590, 630, 910, 1310	0.9886	0.9916	0.9252	0.9823	0.2892	0.2494	0.7587	0.3634
BRI	350, 790, 990, 1870	0.8794	0.8446	0.8711	0.8799	0.0785	0.0907	0.0810	0.0785
SPADI	630, 670, 910	0.9950	0.9982	0.9968	0.9944	0.0052	0.0031	0.0042	0.0058
GM1	510, 550, 590, 630, 910, 1310	0.9880	0.9912	0.9237	0.9821	0.2938	0.2526	0.7630	0.3626
RVI1	590, 790, 2310	0.9825	0.9767	0.9496	0.9784	0.2225	0.2639	0.3874	0.2467
CRİ1	390, 430, 470, 510, 630, 830, 870, 1270	0.9856	0.9883	0.8641	0.9767	0.5746	0.5325	1.7212	0.7236
RVSI	750, 790, 830, 2310	0.9974	0.9987	0.9976	0.9972	0.0064	0.0045	0.0062	0.0066
NDVI1	470, 590, 630, 670, 910, 2470	0.9869	0.9915	0.9858	0.9868	0.0180	0.0147	0.0189	0.0184
NDVI3	510, 590, 630, 710, 870, 910, 1310, 1710, 2150, 2310	0.9795	0.9987	0.9959	0.9807	0.0165	0.0042	0.0074	0.0160
NDVI6	470, 510, 590, 630, 670, 870, 950, 1350, 2310	0.9855	0.9927	0.9891	0.9843	0.0187	0.0133	0.0162	0.0195
PSNDB	510, 590, 630, 670, 910, 1310, 2470	0.9898	0.9970	0.9937	0.9902	0.0152	0.0082	0.0120	0.0151
LIC2	470, 510, 590, 630, 670, 950, 1390	0.9880	0.9941	0.9893	0.9866	0.0153	0.0108	0.0145	0.0164

Results for 40 nm combined stages

VIs	Inputs	R^2				$RMSE$			
		RF	MLP	SVR	kNN	RF	MLP	SVR	kNN
TM57	700, 1100, 2150, 2200, 2250, 2300, 2350, 2400,	0.9508	0.9802	0.9673	0.9429	0.0421	0.0265	0.0354	0.0466
FWBI	750, 1500	0.8013	0.8280	0.8637	0.8257	0.0009	0.0009	0.0008	0.0009
LWI	350, 1100, 1500, 2150, 2200	0.9560	0.9935	0.9795	0.9566	0.0513	0.0194	0.0363	0.0538
SIWSI	350, 1100, 1500, 2200	0.9350	0.9193	0.9609	0.9271	0.0115	0.0123	0.0086	0.0124
VOG1	550, 700, 1300	0.9203	0.9136	0.8945	0.9131	0.0430	0.0447	0.0488	0.0475
SIPI	350, 1150	0.3200	0.3035	0.4744	0.2696	0.0042	0.0045	0.0037	0.0041
VOPT	450, 650, 800	0.9678	0.9990	0.9959	0.9692	0.1025	0.0182	0.0373	0.1046
TCARI	700, 750	0.5407	0.3172	0.4039	0.5564	0.0265	0.0317	0.0283	0.0258
SAVI	450, 650, 1150, 2000	0.9432	0.9887	0.9949	0.9437	0.0180	0.0080	0.0052	0.0203
SR800550	550, 600, 700, 1300	0.9761	0.9909	0.9400	0.9752	0.3564	0.2167	0.5865	0.4141
CIIRE	550, 700, 1300	0.9548	0.9554	0.9227	0.9526	0.1343	0.1349	0.1740	0.1517
LCI	550, 600, 700, 1300	0.9587	0.9890	0.9917	0.9565	0.0136	0.0068	0.0060	0.0162
ARI	550, 700, 1300	0.7834	0.8982	0.7307	0.7329	0.2586	0.1889	0.2845	0.2840
SPRI2	400, 550, 700, 1300	0.9689	0.9888	0.9436	0.9750	0.3741	0.2339	0.5299	0.3889
BRI	550, 700, 1300	0.5298	0.4522	0.5479	0.4462	0.0752	0.0862	0.0735	0.0793
SPADI	350, 650, 800	0.9294	0.9938	0.9986	0.9069	0.0117	0.0035	0.0018	0.0151
GM1	400, 550, 700, 1300	0.9700	0.9895	0.9434	0.9755	0.3596	0.2177	0.5164	0.3787
RVI1	550, 700, 1300	0.9722	0.9838	0.9433	0.9728	0.2009	0.1531	0.2890	0.2308
CRİ1	400, 450, 800	0.9895	0.9847	0.8781	0.9698	0.5844	0.7035	2.0910	1.0195
RVSI	700, 800, 850, 1100	0.9751	0.9785	0.9888	0.9771	0.0075	0.0072	0.0050	0.0078
NDVI1	450, 650, 800	0.9645	0.9940	0.9974	0.9428	0.0152	0.0061	0.0039	0.0210
NDVI3	550, 700, 1300	0.9241	0.9254	0.9095	0.9085	0.0148	0.0146	0.0159	0.0171
NDVI6	550, 600, 700, 1300, 1950	0.9583	0.9949	0.9950	0.9549	0.0142	0.0048	0.0048	0.0172
PSNDB	400, 450, 500, 600, 650, 1300, 1950	0.9644	0.9937	0.9968	0.9519	0.0146	0.0062	0.0041	0.0188
LIC2	450, 650, 800	0.9653	0.9943	0.9976	0.9420	0.0142	0.0055	0.0035	0.0200

Results for 50 nm fresh stage

VIs	Inputs	R^2				$RMSE$			
		RF	MLP	SVR	kNN	RF	MLP	SVR	kNN
TM57	350, 2200, 2250, 2300, 2350, 2400, 2450	0.9576	0.9473	0.9478	0.9635	0.0355	0.0406	0.0393	0.0340
FWBI	350, 750	0.9352	0.9194	0.9476	0.9210	0.0030	0.0034	0.0027	0.0034
LWI	350, 1450	0.9107	0.8773	0.9237	0.9140	0.0583	0.0737	0.0541	0.0576
SIWSI	750, 900, 1450, 2000	0.9120	0.9574	0.9630	0.9253	0.0157	0.0099	0.0093	0.0152
VOG1	350, 700, 2100	0.7159	0.7090	0.7731	0.7503	0.0600	0.0620	0.0564	0.0565
SIPI	600, 650, 1350	0.6113	0.3913	0.4280	0.5332	0.0050	0.0076	0.0056	0.0053
VOPT	650, 850	0.9533	0.9746	0.9780	0.9677	0.1098	0.0787	0.0758	0.0951
TCARI	350, 750	0.6912	0.6534	0.6544	0.6381	0.0223	0.0244	0.0233	0.0236
SAVI	450, 650, 900	0.9171	0.9477	0.9581	0.9244	0.0264	0.0213	0.0191	0.0256
SR800550	350, 550, 600	0.9652	0.9649	0.8892	0.9542	0.6403	0.6489	1.1374	0.7344
CIIRE	700	0.6838	0.7296	0.7963	0.7447	0.2192	0.2097	0.1854	0.1955
LCI	350, 600, 650, 700	0.9664	0.9613	0.9770	0.9629	0.0146	0.0163	0.0122	0.0155
ARI	550, 750	0.9551	0.9398	0.8467	0.9133	0.7909	0.9229	1.4548	1.0956
SPRI2	550, 600	0.9482	0.9232	0.8751	0.9423	0.5927	0.7239	0.9213	0.6230
BRI	650, 1350	0.6473	0.4888	0.6036	0.5873	0.0752	0.0921	0.0778	0.0803
SPADI	350, 650, 1100	0.9845	0.9983	0.9978	0.9785	0.0068	0.0022	0.0025	0.0091
GM1	550, 600	0.9513	0.9245	0.8788	0.9454	0.5878	0.7342	0.9265	0.6201
RVI1	350, 500, 650, 700	0.7908	0.7398	0.7933	0.7573	0.3133	0.3609	0.3233	0.3346
CRİ1	400, 900	0.8981	0.8673	0.7885	0.8775	1.0449	1.2000	1.4698	1.1406
RVSI	750, 800	0.9856	0.9848	0.9851	0.9801	0.0079	0.0080	0.0082	0.0097
NDVI1	650, 1100	0.9552	0.9616	0.9758	0.9547	0.0245	0.0227	0.0181	0.0258
NDVI3	350, 700, 2150	0.8689	0.8795	0.8914	0.8827	0.0202	0.0198	0.0186	0.0192
NDVI6	600, 650, 1100	0.9287	0.9414	0.9400	0.9204	0.0230	0.0210	0.0216	0.0246
PSNDB	650, 1100	0.9719	0.9756	0.9840	0.9688	0.0168	0.0156	0.0127	0.0190
LIC2	650, 1100	0.9686	0.9733	0.9847	0.9675	0.0185	0.0171	0.0130	0.0202

Results for 50 nm first dehydration stage

VIs	Inputs	R^2				$RMSE$			
		RF	MLP	SVR	kNN	RF	MLP	SVR	kNN
TM57	1100, 2150, 2200, 2250, 2300, 2400, 2450	0.9752	0.9840	0.9825	0.9783	0.0273	0.0219	0.0230	0.0277
FWBI	750, 2200	0.8883	0.9025	0.9106	0.9050	0.0029	0.0028	0.0026	0.0028
LWI	350, 1100, 1450, 1500, 2100	0.9767	0.9979	0.9878	0.9779	0.0295	0.0089	0.0225	0.0298
SIWSI	1050, 1450	0.8095	0.8075	0.8671	0.8302	0.0143	0.0147	0.0124	0.0137
VOG1	700, 1100, 2150	0.9094	0.9096	0.9082	0.8929	0.0316	0.0334	0.0316	0.0345
SIPI	350, 900	0.2556	0.4188	0.3619	0.3948	0.0095	0.0083	0.0083	0.0084
VOPT	450, 650, 900	0.9179	0.9442	0.9585	0.9340	0.0981	0.0848	0.0717	0.0921
TCARI	750	0.6325	0.5968	0.7300	0.6940	0.0229	0.0238	0.0193	0.0204
SAVI	450, 650, 900	0.9336	0.9615	0.9729	0.9498	0.0224	0.0186	0.0148	0.0208
SR800550	400, 550, 600, 1300	0.9841	0.9884	0.9340	0.9773	0.3349	0.2896	0.6888	0.4125
CIIRE	700, 1100, 2400	0.9494	0.9576	0.9450	0.9348	0.0740	0.0725	0.0762	0.0872
LCI	550, 700, 1100, 1950	0.9844	0.9948	0.9918	0.9757	0.0115	0.0070	0.0082	0.0156
ARI	550, 1100	0.9577	0.9632	0.8597	0.9520	0.5296	0.5089	0.9680	0.5767
SPRI2	400, 550, 600, 1300	0.9757	0.9787	0.9351	0.9668	0.3001	0.2834	0.4907	0.3563
BRI	350, 1350	0.6564	0.5277	0.6286	0.6195	0.0669	0.0768	0.0676	0.0688
SPADI	350, 650, 1100	0.9920	0.9992	0.9986	0.9787	0.0060	0.0018	0.0023	0.0111
GM1	400, 550, 600, 1300	0.9752	0.9795	0.9344	0.9681	0.3145	0.2883	0.5121	0.3629
RVI1	700, 1100	0.9488	0.9622	0.9588	0.9467	0.1172	0.1070	0.1057	0.1217
CRİ1	400, 900	0.9554	0.9284	0.8652	0.9535	0.5378	0.7291	0.9226	0.5547
RVSI	800, 900	0.9581	0.9672	0.9760	0.9562	0.0063	0.0058	0.0047	0.0066
NDVI1	400, 450, 500, 600, 650, 1200	0.9763	0.9882	0.9861	0.9752	0.0202	0.0147	0.0157	0.0214
NDVI3	700, 750, 1950	0.9402	0.9241	0.9319	0.9227	0.0150	0.0171	0.0158	0.0174
NDVI6	550, 600, 650, 700, 1100	0.9733	0.9861	0.9839	0.9743	0.0151	0.0115	0.0118	0.0154
PSNDB	450, 500, 600, 650, 1200	0.9805	0.9901	0.9901	0.9848	0.0158	0.0115	0.0114	0.0151
LIC2	400, 450, 500, 600, 650, 1200	0.9809	0.9926	0.9907	0.9804	0.0165	0.0107	0.0117	0.0176

Results for 50 nm second dehydration stage

VIs	Inputs	R^2				$RMSE$			
		RF	MLP	SVR	kNN	RF	MLP	SVR	kNN
TM57	2250, 2350	0.9502	0.9194	0.9569	0.9526	0.0325	0.0421	0.0303	0.0322
FWBI	750, 800, 1500	0.8564	0.8485	0.8853	0.8615	0.0030	0.0032	0.0027	0.0030
LWI	1450, 2050	0.9560	0.9397	0.9620	0.9631	0.0328	0.0396	0.0312	0.0303
SIWSI	1050, 1100, 1450, 1900, 2000	0.7293	0.7854	0.7901	0.7131	0.0143	0.0131	0.0129	0.0146
VOG1	700, 2450	0.9076	0.8931	0.8937	0.9189	0.0285	0.0310	0.0312	0.0273
SIPI	700	0.3861	0.4098	0.3894	0.3864	0.0088	0.0084	0.0081	0.0083
VOPT	450, 650, 950	0.9313	0.9378	0.9576	0.9439	0.0973	0.0923	0.0765	0.0982
TCARI	750, 800, 900	0.5202	0.6210	0.6544	0.5496	0.0272	0.0253	0.0243	0.0263
SAVI	400, 450, 500, 650, 950, 1150	0.9426	0.9644	0.9768	0.9425	0.0233	0.0185	0.0150	0.0261
SR800550	350, 400, 550, 600, 650, 1150	0.9875	0.9919	0.9095	0.9718	0.2825	0.2243	0.9191	0.4362
CIIRE	700, 1950	0.9486	0.9335	0.9315	0.9431	0.0663	0.0755	0.0772	0.0727
LCI	650, 700	0.9784	0.9736	0.9842	0.9764	0.0142	0.0155	0.0120	0.0152
ARI	350, 550, 600, 1100	0.9623	0.9428	0.8572	0.9471	0.4704	0.5784	0.9910	0.5619
SPRI2	350, 400, 550, 600, 650, 1150	0.9826	0.9869	0.9120	0.9687	0.2405	0.2073	0.6517	0.3325
BRI	350, 1250	0.4166	0.2440	0.4263	0.3358	0.0769	0.0909	0.0738	0.0786
SPADI	450, 650, 1100	0.9840	0.9977	0.9985	0.9830	0.0093	0.0034	0.0028	0.0136
GM1	350, 400, 550, 600, 650, 1150	0.9834	0.9871	0.9104	0.9692	0.2462	0.2162	0.6717	0.3459
RVI1	700, 1100	0.9534	0.9619	0.9371	0.9432	0.1033	0.0948	0.1205	0.1195
CRİ1	350, 400, 450, 1150	0.9832	0.9796	0.8246	0.9480	0.3742	0.4085	1.2965	0.6511
RVSI	800, 1050	0.9770	0.9740	0.9780	0.9729	0.0044	0.0050	0.0044	0.0051
NDVI1	400, 450, 500, 600, 650, 1150	0.9763	0.9922	0.9842	0.9760	0.0226	0.0129	0.0190	0.0263
NDVI3	700, 2450	0.9393	0.9271	0.9414	0.9431	0.0146	0.0161	0.0145	0.0147
NDVI6	600, 650, 700, 1100	0.9748	0.9758	0.9794	0.9696	0.0162	0.0158	0.0146	0.0198
PSNDB	400, 450, 500, 600, 650, 1150	0.9803	0.9911	0.9872	0.9783	0.0181	0.0119	0.0148	0.0219
LIC2	400, 450, 500, 600, 650, 1000	0.9797	0.9942	0.9897	0.9809	0.0194	0.0102	0.0140	0.0221

Results for 50 nm third dehydration stage

VIs	Inputs	R^2				$RMSE$			
		RF	MLP	SVR	kNN	RF	MLP	SVR	kNN
TM57	1300, 2250, 2300, 2350	0.9559	0.9680	0.9696	0.9517	0.0219	0.0187	0.0180	0.0243
FWBI	750, 2500	0.8659	0.8742	0.8871	0.8781	0.0026	0.0026	0.0024	0.0024
LWI	2050, 2500	0.8468	0.7905	0.8462	0.8429	0.0161	0.0202	0.0160	0.0161
SIWSI	750, 800, 850, 900	0.6135	0.5655	0.6778	0.6253	0.0109	0.0115	0.0099	0.0106
VOG1	700, 1000	0.9522	0.9524	0.9529	0.9423	0.0129	0.0141	0.0128	0.0144
SIPI	650, 950	0.4687	0.3703	0.5561	0.4970	0.0119	0.0137	0.0112	0.0116
VOPT	450, 650, 950	0.9408	0.9507	0.9679	0.9502	0.0839	0.0782	0.0619	0.0808
TCARI	800, 850, 900, 1100, 1900, 1950, 2000	0.5546	0.3901	0.5681	0.5619	0.0310	0.0370	0.0306	0.0310
SAVI	450, 650, 950	0.9528	0.9620	0.9761	0.9575	0.0212	0.0194	0.0151	0.0214
SR800550	350, 550, 600, 650, 1000	0.9742	0.9835	0.9434	0.9630	0.2588	0.2095	0.3909	0.3156
CIIRE	700, 1000	0.9584	0.9626	0.9583	0.9530	0.0394	0.0411	0.0396	0.0430
LCI	350, 550, 600, 650, 700, 1000	0.9732	0.9863	0.9841	0.9668	0.0154	0.0114	0.0121	0.0176
ARI	350, 400, 450, 500, 550, 600, 1000	0.9648	0.9736	0.9083	0.9380	0.2885	0.2578	0.4801	0.3822
SPRI2	350, 600, 650, 1000	0.9744	0.9815	0.9414	0.9611	0.1852	0.1583	0.2853	0.2319
BRI	350, 400, 2250	0.6548	0.4820	0.6448	0.6399	0.0598	0.0732	0.0604	0.0618
SPADI	450, 500, 550, 600, 650, 1000	0.9763	0.9925	0.9929	0.9751	0.0120	0.0068	0.0066	0.0132
GM1	350, 600, 650, 1000	0.9741	0.9810	0.9412	0.9594	0.1957	0.1684	0.2992	0.2480
RVI1	350, 600, 650, 700, 1000, 1950	0.9583	0.9634	0.9567	0.9315	0.0663	0.0662	0.0673	0.0854
CRİ1	350, 400, 600, 650, 1000	0.9697	0.9793	0.8718	0.9334	0.2837	0.2372	0.5773	0.4188
RVSI	800, 850	0.9873	0.9888	0.9924	0.9839	0.0030	0.0030	0.0023	0.0035
NDVI1	450, 600, 650, 1000	0.9704	0.9864	0.9761	0.9647	0.0249	0.0170	0.0225	0.0284
NDVI3	700, 1000	0.9546	0.9634	0.9635	0.9475	0.0103	0.0097	0.0092	0.0113
NDVI6	600, 650, 700, 1000	0.9675	0.9763	0.9783	0.9688	0.0175	0.0154	0.0145	0.0181
PSNDB	550, 600, 650, 1000	0.9759	0.9890	0.9851	0.9726	0.0193	0.0131	0.0152	0.0220
LIC2	450, 500, 600, 650, 1000	0.9725	0.9881	0.9825	0.9706	0.0225	0.0149	0.0181	0.0245

Results for 50 nm fully dry stage

VIs	Inputs	R^2				$RMSE$			
		RF	MLP	SVR	kNN	RF	MLP	SVR	kNN
TM57	900, 1700, 2250, 2300	0.9968	0.9985	0.9875	0.9940	0.0236	0.0163	0.0469	0.0324
FWBI	750, 800, 850, 1150, 1250	0.9940	0.9958	0.9918	0.9936	0.0018	0.0015	0.0022	0.0019
LWI	850, 900, 950, 1550, 1600, 1650, 1700, 1750, 2300	0.9970	0.9996	0.9932	0.9964	0.0320	0.0118	0.0475	0.0346
SIWSI	800, 900, 1300, 1850, 2300	0.9971	0.9990	0.9978	0.9962	0.0089	0.0052	0.0077	0.0101
VOG1	700, 850, 1750	0.9864	0.9773	0.9616	0.9854	0.0359	0.0464	0.0606	0.0370
SIPI	650, 700, 1000, 1950	0.7675	0.7028	0.7176	0.7450	0.0079	0.0092	0.0087	0.0082
VOPT	500, 650, 800, 2300	0.9962	0.9989	0.9958	0.9946	0.0517	0.0282	0.0544	0.0612
TCARI	700, 1000, 1150, 1200, 1700, 1800, 1950	0.8712	0.8306	0.7880	0.8434	0.0240	0.0281	0.0303	0.0264
SAVI	600, 650, 900, 2300	0.9822	0.9821	0.9797	0.9797	0.0189	0.0189	0.0201	0.0203
SR800550	550, 600, 700, 1050	0.9893	0.9885	0.8951	0.9797	0.3229	0.3480	1.0435	0.4454
CIIRE	700, 800, 2300	0.9909	0.9871	0.9634	0.9903	0.0963	0.1172	0.1955	0.0987
LCI	500, 600, 650, 700, 1050, 2300	0.9910	0.9953	0.9929	0.9860	0.0114	0.0082	0.0102	0.0143
ARI	350, 400, 550, 750	0.9734	0.9496	0.8783	0.9683	0.5683	0.7829	1.1951	0.6198
SPRI2	550, 600, 700, 900	0.9891	0.9883	0.9138	0.9819	0.2831	0.3043	0.8210	0.3657
BRI	350, 950, 1400	0.8815	0.8416	0.8715	0.8798	0.0778	0.0922	0.0808	0.0784
SPADI	650, 950, 2500	0.9927	0.9983	0.9970	0.9878	0.0063	0.0030	0.0040	0.0090
GM1	400, 500, 550, 600, 650, 700, 900, 950, 2300	0.9877	0.9961	0.9319	0.9782	0.2993	0.1694	0.7241	0.3987
RVI1	700, 800, 2300	0.9940	0.9951	0.9619	0.9930	0.1321	0.1230	0.3394	0.1420
CRİ1	400, 450, 600, 850, 1250	0.9843	0.9792	0.8583	0.9728	0.5985	0.6986	1.7500	0.7808
RVSI	700, 750, 800, 1850	0.9981	0.9984	0.9981	0.9964	0.0055	0.0050	0.0055	0.0076
NDVI1	450, 500, 600, 650, 700, 900	0.9876	0.9922	0.9866	0.9864	0.0176	0.0141	0.0184	0.0184
NDVI3	600, 700, 900, 1200, 2300	0.9832	0.9943	0.9912	0.9765	0.0150	0.0087	0.0108	0.0176
NDVI6	600, 700, 950, 2300	0.9844	0.9813	0.9781	0.9833	0.0194	0.0212	0.0229	0.0201
PSNDB	500, 600, 650, 700, 900, 1400	0.9915	0.9950	0.9915	0.9899	0.0138	0.0106	0.0139	0.0154
LIC2	450, 500, 600, 650, 700, 950	0.9883	0.9898	0.9884	0.9863	0.0151	0.0144	0.0151	0.0165

Results for 50 nm combined stages

VIs	Inputs	R^2				$RMSE$			
		RF	MLP	SVR	kNN	RF	MLP	SVR	kNN
TM57	1100, 2075, 2150, 2225, 2300, 2375, 2450	0.9473	0.9797	0.9706	0.9399	0.0434	0.0276	0.0327	0.0473
FWBI	350, 725, 800, 875, 1475	0.8169	0.8423	0.8710	0.8230	0.0009	0.0009	0.0008	0.0009
LWI	1100, 1475, 1550, 2150, 2225	0.9645	0.9968	0.9913	0.9668	0.0472	0.0135	0.0227	0.0481
SIWSI	350, 1100, 1475, 2225	0.9267	0.9044	0.9418	0.9151	0.0120	0.0135	0.0106	0.0131
VOG1	575, 725	0.8136	0.6836	0.7781	0.8126	0.0636	0.0872	0.0692	0.0642
SIPI	350, 725	0.3209	0.4587	0.5175	0.3958	0.0042	0.0039	0.0036	0.0039
VOPT	500, 650, 800	0.9668	0.9988	0.9961	0.9699	0.1042	0.0199	0.0366	0.1037
TCARI	425, 725	0.7893	0.8155	0.8516	0.8189	0.0190	0.0182	0.0162	0.0177
SAVI	425, 500, 650, 1100	0.9503	0.9900	0.9957	0.9559	0.0172	0.0077	0.0047	0.0178
SR800550	575, 1100	0.9748	0.9571	0.9132	0.9526	0.3666	0.4755	0.6879	0.5337
CIIRE	575, 725	0.8649	0.7726	0.8222	0.8675	0.2245	0.3024	0.2581	0.2255
LCI	575, 1100	0.9418	0.9333	0.9651	0.9135	0.0157	0.0168	0.0121	0.0209
ARI	575, 1250, 1925	0.6591	0.6564	0.5858	0.6932	0.3158	0.3278	0.3396	0.3027
SPRI2	350, 575, 1100	0.9774	0.9852	0.9261	0.9449	0.3433	0.2703	0.5956	0.5378
BRI	350, 575, 1325, 1925	0.7050	0.6930	0.7331	0.6873	0.0621	0.0639	0.0610	0.0638
SPADI	350, 650, 800	0.9294	0.9938	0.9986	0.9069	0.0117	0.0035	0.0018	0.0151
GM1	350, 575, 1100	0.9766	0.9833	0.9276	0.9448	0.3373	0.2740	0.5737	0.5268
RVI1	575, 1100	0.9306	0.8598	0.8813	0.9156	0.3101	0.4551	0.4049	0.3573
CRİ1	425, 800	0.9898	0.9789	0.8817	0.9660	0.5770	0.8281	2.0504	1.0902
RVSI	575, 800, 875, 1100	0.9719	0.9697	0.9822	0.9693	0.0079	0.0086	0.0064	0.0087
NDVI1	350, 650, 800	0.9498	0.9943	0.9971	0.9157	0.0179	0.0059	0.0041	0.0254
NDVI3	575, 725	0.8236	0.7060	0.8099	0.8139	0.0218	0.0294	0.0227	0.0225
NDVI6	575, 1100	0.9515	0.9681	0.9838	0.9272	0.0149	0.0122	0.0085	0.0203
PSNDB	500, 650, 800	0.9673	0.9930	0.9971	0.9431	0.0142	0.0063	0.0039	0.0203
LIC2	500, 650, 800	0.9666	0.9941	0.9977	0.9435	0.0140	0.0056	0.0034	0.0199

Results for 75 nm fresh stage

VIs	Inputs	R^2				$RMSE$			
		RF	MLP	SVR	kNN	RF	MLP	SVR	kNN
TM57	350, 2150, 2225, 2300, 2375, 2450	0.9545	0.9464	0.9487	0.9569	0.0368	0.0410	0.0390	0.0366
FWBI	725, 800	0.9174	0.8857	0.9253	0.9143	0.0033	0.0040	0.0032	0.0034
LWI	350, 1475	0.9134	0.8780	0.9251	0.9086	0.0574	0.0737	0.0537	0.0591
SIWSI	875, 2000	0.8387	0.8456	0.8748	0.8656	0.0188	0.0182	0.0169	0.0180
VOG1	350, 650, 2000, 2150	0.7161	0.6865	0.7344	0.6985	0.0597	0.0641	0.0598	0.0611
SIPI	575, 650, 1325	0.5217	0.5075	0.4070	0.5050	0.0055	0.0060	0.0057	0.0055
VOPT	500, 650, 800, 875	0.9632	0.9987	0.9960	0.9726	0.1013	0.0180	0.0323	0.0897
TCARI	725	0.6213	0.6675	0.6995	0.7012	0.0249	0.0245	0.0220	0.0220
SAVI	500, 650, 950	0.8887	0.9106	0.9258	0.8837	0.0302	0.0274	0.0249	0.0310
SR800550	350, 575	0.9619	0.9341	0.8912	0.9562	0.6687	0.8927	1.1401	0.7240
CIIRE	350, 425, 650, 2150	0.7559	0.7142	0.7744	0.7792	0.1913	0.2135	0.1933	0.1843
LCI	350, 425, 575, 650, 2375	0.9524	0.9546	0.9591	0.9475	0.0173	0.0170	0.0162	0.0184
ARI	350, 500, 575, 725	0.9126	0.8959	0.7975	0.8435	1.0915	1.1890	1.6231	1.4550
SPRI2	575	0.9236	0.9043	0.8787	0.9416	0.7158	0.8246	0.9097	0.6281
BRI	650, 1325	0.5304	0.4480	0.5628	0.5968	0.0851	0.0961	0.0806	0.0787
SPADI	350, 650, 1100	0.9845	0.9983	0.9978	0.9785	0.0068	0.0022	0.0025	0.0091
GM1	575	0.9292	0.9074	0.8821	0.9446	0.7049	0.8297	0.9179	0.6252
RVI1	350, 425, 500, 650, 950	0.7783	0.7624	0.8155	0.7774	0.3227	0.3362	0.3029	0.3230
CRİ1	425, 875	0.8949	0.8928	0.7910	0.8980	1.0464	1.0694	1.4643	1.0399
RVSI	800	0.9835	0.9846	0.9822	0.9781	0.0085	0.0081	0.0098	0.0100
NDVI1	650, 1100	0.9552	0.9616	0.9758	0.9547	0.0245	0.0227	0.0181	0.0258
NDVI3	350, 650, 2150	0.8273	0.8117	0.8465	0.8425	0.0229	0.0239	0.0218	0.0221
NDVI6	350, 650, 1100	0.9191	0.9368	0.9454	0.9092	0.0245	0.0218	0.0203	0.0262
PSNDB	650, 1100	0.9719	0.9756	0.9840	0.9688	0.0168	0.0156	0.0127	0.0190
LIC2	650, 1100	0.9686	0.9733	0.9847	0.9675	0.0185	0.0171	0.0130	0.0202

Results for 75 nm first dehydration stage

VIs	Inputs	R^2				$RMSE$			
		RF	MLP	SVR	kNN	RF	MLP	SVR	kNN
TM57	1100, 2075, 2150, 2225, 2300, 2375, 2450	0.9719	0.9854	0.9817	0.9766	0.0289	0.0208	0.0237	0.0284
FWBI	725, 800	0.8630	0.8984	0.9047	0.8922	0.0032	0.0030	0.0027	0.0029
LWI	350, 1100, 1475, 2075	0.9769	0.9921	0.9796	0.9756	0.0294	0.0171	0.0290	0.0319
SIWSI	1100, 1475	0.7971	0.7964	0.8594	0.8252	0.0147	0.0151	0.0127	0.0138
VOG1	425, 725, 1925	0.9027	0.8636	0.9076	0.9144	0.0325	0.0388	0.0316	0.0309
SIPI	350, 875	0.2694	0.4249	0.3596	0.5035	0.0094	0.0082	0.0083	0.0076
VOPT	425, 650, 875	0.9379	0.9673	0.9754	0.9527	0.0867	0.0658	0.0548	0.0791
TCARI	725, 800	0.6908	0.5553	0.6928	0.7373	0.0209	0.0242	0.0204	0.0191
SAVI	425, 500, 575, 650, 875, 950	0.9486	0.9983	0.9976	0.9635	0.0201	0.0040	0.0043	0.0183
SR800550	575, 1100	0.9829	0.9824	0.9321	0.9759	0.3464	0.3553	0.6992	0.4326
CIIRE	575, 650, 725, 1925	0.9227	0.8772	0.9079	0.9255	0.0901	0.1156	0.0978	0.0890
LCI	425, 500, 575, 650, 725, 2300	0.9730	0.9679	0.9696	0.9692	0.0150	0.0162	0.0157	0.0165
ARI	575, 1100	0.9610	0.9605	0.8569	0.9493	0.5101	0.5265	0.9741	0.5857
SPRI2	575, 1100	0.9715	0.9684	0.9347	0.9624	0.3241	0.3449	0.4939	0.3819
BRI	350, 1325	0.6437	0.5291	0.6393	0.6461	0.0681	0.0766	0.0668	0.0665
SPADI	350, 650, 1100	0.9920	0.9992	0.9986	0.9787	0.0060	0.0018	0.0023	0.0111
GM1	575, 1100	0.9713	0.9701	0.9345	0.9633	0.3373	0.3482	0.5126	0.3915
RVI1	425, 500, 575, 650, 725	0.9404	0.9288	0.9178	0.9300	0.1260	0.1441	0.1475	0.1379
CRİ1	425, 1175	0.9644	0.9395	0.8629	0.9421	0.4802	0.6484	0.9495	0.6172
RVSI	800, 875	0.9571	0.9665	0.9759	0.9582	0.0063	0.0059	0.0047	0.0064
NDVI1	425, 500, 575, 650, 1175	0.9783	0.9906	0.9871	0.9756	0.0193	0.0130	0.0151	0.0214
NDVI3	650, 725, 1925	0.9256	0.8914	0.9218	0.9319	0.0166	0.0201	0.0170	0.0161
NDVI6	425, 500, 575, 650, 1250	0.9723	0.9771	0.9745	0.9636	0.0154	0.0147	0.0148	0.0182
PSNDB	425, 500, 575, 650, 1175	0.9793	0.9903	0.9896	0.9828	0.0163	0.0114	0.0116	0.0159
LIC2	425, 500, 575, 650, 1175	0.9830	0.9938	0.9912	0.9810	0.0156	0.0097	0.0114	0.0175

Results for 75 nm second dehydration stage

VIs	Inputs	R^2				$RMSE$			
		RF	MLP	SVR	kNN	RF	MLP	SVR	kNN
TM57	2150, 2300	0.9383	0.9178	0.9548	0.9455	0.0359	0.0428	0.0309	0.0340
FWBI	725, 800	0.8813	0.8345	0.8803	0.8649	0.0028	0.0034	0.0028	0.0030
LWI	1475, 2075	0.9590	0.9352	0.9604	0.9579	0.0317	0.0410	0.0317	0.0322
SIWSI	1100, 1475, 2000	0.7155	0.6888	0.7745	0.6974	0.0146	0.0155	0.0133	0.0149
VOG1	650, 725, 2450	0.8612	0.8641	0.8765	0.8753	0.0345	0.0350	0.0327	0.0332
SIPI	650, 875	0.4697	0.3735	0.3237	0.5199	0.0080	0.0085	0.0083	0.0075
VOPT	425, 650, 950	0.9334	0.9356	0.9576	0.9419	0.0960	0.0939	0.0764	0.0986
TCARI	725, 800, 875	0.5821	0.6752	0.6418	0.5530	0.0256	0.0241	0.0245	0.0263
SAVI	425, 650, 1025	0.9302	0.9404	0.9652	0.9451	0.0255	0.0238	0.0183	0.0261
SR800550	350, 425, 500, 575, 650, 1025	0.9873	0.9927	0.9089	0.9734	0.2844	0.2144	0.9218	0.4173
CIIRE	575, 650, 725	0.9018	0.8956	0.8971	0.8816	0.0905	0.0937	0.0924	0.0992
LCI	425, 500, 575, 650, 725	0.9628	0.9611	0.9724	0.9591	0.0185	0.0188	0.0159	0.0200
ARI	350, 575, 1100	0.9609	0.9432	0.8555	0.9409	0.4787	0.5755	0.9624	0.5930
SPRI2	350, 425, 575, 650, 1025	0.9826	0.9874	0.9070	0.9674	0.2407	0.2046	0.6579	0.3356
BRI	350, 1250	0.4166	0.2440	0.4263	0.3358	0.0769	0.0909	0.0738	0.0786
SPADI	425, 500, 575, 650, 1100	0.9837	0.9980	0.9985	0.9809	0.0096	0.0033	0.0027	0.0131
GM1	350, 425, 500, 575, 650, 1025	0.9841	0.9877	0.9088	0.9721	0.2415	0.2126	0.6930	0.3253
RVI1	425, 500, 575, 650, 725	0.9258	0.9292	0.8990	0.9128	0.1292	0.1261	0.1510	0.1408
CRİ1	350, 425, 1025	0.9783	0.9749	0.8203	0.9496	0.4244	0.4553	1.2442	0.6546
RVSI	800, 1025	0.9771	0.9738	0.9779	0.9708	0.0044	0.0050	0.0044	0.0052
NDVI1	425, 500, 575, 650, 1025	0.9770	0.9951	0.9842	0.9763	0.0223	0.0104	0.0191	0.0261
NDVI3	650, 725, 1925	0.9146	0.8967	0.9290	0.9091	0.0173	0.0190	0.0158	0.0182
NDVI6	575, 650, 1100	0.9653	0.9531	0.9689	0.9584	0.0187	0.0218	0.0178	0.0225
PSNDB	425, 500, 575, 650, 1025	0.9801	0.9922	0.9858	0.9775	0.0182	0.0112	0.0154	0.0223
LIC2	425, 500, 575, 650, 1025	0.9799	0.9955	0.9896	0.9794	0.0193	0.0092	0.0140	0.0232

Results for 75 nm third dehydration stage

VIs	Inputs	R^2				$RMSE$			
		RF	MLP	SVR	kNN	RF	MLP	SVR	kNN
TM57	1325, 2300	0.9490	0.9554	0.9570	0.9403	0.0232	0.0219	0.0216	0.0273
FWBI	725	0.8313	0.8637	0.8808	0.8528	0.0029	0.0027	0.0024	0.0026
LWI	2075, 2450	0.8070	0.7475	0.8215	0.7958	0.0178	0.0217	0.0171	0.0182
SIWSI	800, 875	0.5867	0.5369	0.6718	0.5967	0.0113	0.0117	0.0100	0.0109
VOG1	350, 575, 650, 725	0.9231	0.9183	0.9152	0.9195	0.0162	0.0174	0.0170	0.0166
SIPI	650, 950	0.4687	0.3703	0.5561	0.4970	0.0119	0.0137	0.0112	0.0116
VOPT	425, 650, 950	0.9367	0.9501	0.9676	0.9491	0.0869	0.0787	0.0622	0.0814
TCARI	800, 875, 1100, 1925, 2000	0.5655	0.4490	0.5651	0.5530	0.0307	0.0355	0.0307	0.0313
SAVI	425, 650, 950	0.9467	0.9611	0.9755	0.9566	0.0225	0.0196	0.0153	0.0216
SR800550	350, 500, 575, 650, 1100	0.9755	0.9822	0.9348	0.9663	0.2525	0.2175	0.4117	0.2993
CIIRE	575, 1925	0.9338	0.9217	0.9258	0.9185	0.0496	0.0554	0.0522	0.0555
LCI	575, 1325	0.9662	0.9735	0.9718	0.9623	0.0172	0.0158	0.0159	0.0194
ARI	350, 425, 500, 575, 1100	0.9575	0.9618	0.9073	0.9386	0.3164	0.3091	0.4766	0.3789
SPRI2	350, 500, 575, 650, 950	0.9749	0.9829	0.9401	0.9651	0.1837	0.1542	0.2862	0.2199
BRI	350, 425, 2225	0.6135	0.4531	0.6312	0.6040	0.0628	0.0747	0.0615	0.0641
SPADI	650, 1100	0.9812	0.9879	0.9914	0.9779	0.0108	0.0086	0.0073	0.0130
GM1	350, 500, 575, 650, 950	0.9750	0.9829	0.9386	0.9645	0.1925	0.1619	0.3061	0.2324
RVI1	350, 425, 500, 575, 650, 1925	0.9525	0.9472	0.9248	0.9229	0.0706	0.0761	0.0890	0.0898
CRİ1	350, 425, 650, 950	0.9721	0.9788	0.8725	0.9354	0.2726	0.2405	0.5765	0.4121
RVSI	800, 875	0.9870	0.9885	0.9920	0.9819	0.0030	0.0030	0.0023	0.0038
NDVI1	425, 500, 650, 950	0.9706	0.9795	0.9779	0.9670	0.0248	0.0211	0.0216	0.0276
NDVI3	575, 650, 725	0.9441	0.9406	0.9369	0.9453	0.0114	0.0121	0.0122	0.0113
NDVI6	575, 650, 1925	0.9618	0.9487	0.9542	0.9452	0.0189	0.0220	0.0208	0.0232
PSNDB	650, 1925	0.9738	0.9663	0.9712	0.9650	0.0202	0.0233	0.0212	0.0256
LIC2	425, 500, 575, 650, 1025	0.9720	0.9892	0.9795	0.9668	0.0228	0.0143	0.0195	0.0257

Results for 75 nm fully dry stage

VIs	Inputs	R^2				$RMSE$			
		RF	MLP	SVR	kNN	RF	MLP	SVR	kNN
TM57	950, 1700, 2150, 2300	0.9964	0.9980	0.9883	0.9940	0.0249	0.0190	0.0452	0.0324
FWBI	350, 800, 1250	0.9923	0.9954	0.9910	0.9895	0.0021	0.0016	0.0023	0.0024
LWI	875, 950, 1550, 1625, 1700, 2300	0.9971	0.9996	0.9932	0.9967	0.0313	0.0118	0.0475	0.0333
SIWSI	800, 875, 1325, 1850, 2300	0.9973	0.9989	0.9977	0.9964	0.0087	0.0055	0.0079	0.0098
VOG1	575, 800, 1700, 2300	0.9791	0.9687	0.9622	0.9754	0.0443	0.0540	0.0592	0.0479
SIPI	650, 1025, 1925	0.7605	0.6980	0.7226	0.7419	0.0080	0.0093	0.0086	0.0083
VOPT	500, 650, 800, 2300	0.9962	0.9989	0.9958	0.9946	0.0517	0.0282	0.0544	0.0612
TCARI	350, 1175, 1250, 1625, 1700, 1925	0.8216	0.7686	0.8070	0.8177	0.0279	0.0330	0.0289	0.0283
SAVI	500, 650, 875, 2300	0.9895	0.9873	0.9889	0.9875	0.0146	0.0160	0.0149	0.0161
SR800550	575, 950, 2150	0.9884	0.9880	0.8999	0.9667	0.3389	0.3641	1.0192	0.5758
CIIRE	500, 575, 800, 875, 1175, 1250, 2300	0.9805	0.9869	0.9589	0.9722	0.1403	0.1158	0.2028	0.1662
LCI	500, 575, 650, 1025, 2300	0.9771	0.9782	0.9759	0.9732	0.0182	0.0178	0.0188	0.0196
ARI	350, 425, 725, 800, 1250	0.9550	0.9227	0.8275	0.9474	0.7374	0.9751	1.4317	0.7943
SPRI2	500, 575, 650, 950, 2300	0.9852	0.9826	0.9102	0.9728	0.3304	0.3567	0.8253	0.4482
BRI	350, 950, 1400	0.8815	0.8416	0.8715	0.8798	0.0778	0.0922	0.0808	0.0784
SPADI	650, 950, 2300	0.9937	0.9983	0.9970	0.9873	0.0059	0.0030	0.0040	0.0089
GM1	500, 575, 650, 950, 2300	0.9866	0.9841	0.9112	0.9733	0.3128	0.3376	0.8173	0.4404
RVI1	575, 800, 2300	0.9857	0.9772	0.9485	0.9833	0.2019	0.2645	0.3889	0.2175
CRİ1	425, 500, 575, 650, 800, 875, 1250	0.9895	0.9921	0.8688	0.9713	0.4943	0.4423	1.7017	0.8042
RVSI	650, 800, 875, 1250	0.9964	0.9976	0.9961	0.9952	0.0075	0.0062	0.0078	0.0087
NDVI1	425, 500, 575, 650, 950, 1325	0.9859	0.9942	0.9870	0.9838	0.0187	0.0120	0.0181	0.0201
NDVI3	425, 500, 575, 650, 875, 950, 1625, 1700, 2150, 2300	0.9645	0.9677	0.9587	0.9625	0.0216	0.0207	0.0234	0.0222
NDVI6	425, 500, 575, 650, 875, 950, 1325, 2300	0.9845	0.9918	0.9866	0.9832	0.0194	0.0141	0.0181	0.0202
PSNDB	425, 500, 575, 650, 875, 1250, 2300	0.9913	0.9981	0.9936	0.9897	0.0140	0.0066	0.0122	0.0155
LIC2	425, 500, 575, 650, 950, 2300	0.9895	0.9943	0.9889	0.9853	0.0144	0.0106	0.0149	0.0171

Results for 75 nm combined stages

VIs	Inputs	R^2				$RMSE$			
		RF	MLP	SVR	kNN	RF	MLP	SVR	kNN
TM57	850, 2250, 2350	0.9514	0.9591	0.9581	0.9396	0.0417	0.0376	0.0394	0.0490
FWBI	750, 1450	0.7953	0.8207	0.8528	0.8211	0.0010	0.0009	0.0008	0.0009
LWI	350, 1050, 1450, 1550, 2150, 2250	0.9532	0.9961	0.9914	0.9597	0.0532	0.0149	0.0224	0.0521
SIWSI	350, 850, 1450, 1550, 2250	0.9380	0.9972	0.9956	0.9429	0.0114	0.0024	0.0030	0.0110
VOG1	550, 1050	0.9020	0.8090	0.8749	0.8687	0.0475	0.0672	0.0529	0.0558
SIPI	350, 1150	0.3200	0.3035	0.4744	0.2696	0.0042	0.0045	0.0037	0.0041
VOPT	450, 650, 850	0.9664	0.9983	0.9958	0.9705	0.1043	0.0234	0.0385	0.1030
TCARI	750, 1450	0.3630	0.3771	0.4254	0.3702	0.0295	0.0308	0.0280	0.0289
SAVI	450, 650, 1150, 1950	0.9428	0.9846	0.9937	0.9457	0.0180	0.0093	0.0057	0.0200
SR800550	550, 850	0.9862	0.9941	0.9480	0.9712	0.2765	0.1786	0.5628	0.4358
CIIRE	550, 1050	0.9384	0.8841	0.9053	0.9205	0.1564	0.2170	0.1915	0.1842
LCI	550, 1050	0.9626	0.9802	0.9872	0.9437	0.0130	0.0093	0.0074	0.0175
ARI	550, 1250	0.6394	0.6413	0.6133	0.6979	0.3239	0.3334	0.3298	0.2989
SPRI2	350, 550, 850	0.9735	0.9961	0.9504	0.9634	0.3596	0.1341	0.5037	0.4661
BRI	350, 550, 1250, 1950	0.7198	0.7135	0.7579	0.7077	0.0608	0.0624	0.0578	0.0620
SPADI	350, 650, 850	0.9302	0.9939	0.9986	0.9112	0.0118	0.0035	0.0017	0.0150
GM1	350, 550, 850	0.9729	0.9972	0.9517	0.9636	0.3532	0.1104	0.4910	0.4560
RVI1	550, 1050	0.9626	0.9429	0.9275	0.9505	0.2331	0.2874	0.3229	0.2845
CRİ1	450, 850	0.9914	0.9830	0.8704	0.9621	0.5280	0.7517	2.1860	1.1551
RVSI	550, 850, 950, 1050	0.9731	0.9783	0.9866	0.9731	0.0078	0.0072	0.0055	0.0084
NDVI1	450, 650, 850	0.9654	0.9936	0.9972	0.9430	0.0151	0.0063	0.0040	0.0210
NDVI3	550, 1050	0.8837	0.8076	0.8890	0.8566	0.0180	0.0235	0.0175	0.0204
NDVI6	450, 550, 650, 1250, 1950	0.9547	0.9894	0.9905	0.9419	0.0151	0.0069	0.0065	0.0186
PSNDB	350, 650, 850	0.9487	0.9923	0.9968	0.9198	0.0175	0.0066	0.0042	0.0243
LIC2	450, 650, 850	0.9654	0.9943	0.9976	0.9429	0.0142	0.0056	0.0035	0.0199

Results for 100 nm fresh stage

VIs	Inputs	R^2				$RMSE$			
		RF	MLP	SVR	kNN	RF	MLP	SVR	kNN
TM57	350, 2150, 2250, 2350, 2450	0.9563	0.9578	0.9498	0.9576	0.0361	0.0361	0.0385	0.0364
FWBI	350, 750	0.9352	0.9194	0.9476	0.9210	0.0030	0.0034	0.0027	0.0034
LWI	350, 1450	0.9107	0.8773	0.9237	0.9140	0.0583	0.0737	0.0541	0.0576
SIWSI	750, 850, 1450, 1950	0.9125	0.9656	0.9698	0.9174	0.0157	0.0089	0.0085	0.0158
VOG1	350, 650, 2050	0.6589	0.6581	0.7286	0.6702	0.0647	0.0661	0.0606	0.0636
SIPI	650, 1350	0.6078	0.2542	0.4331	0.5027	0.0050	0.0070	0.0056	0.0055
VOPT	650, 850	0.9533	0.9746	0.9780	0.9677	0.1098	0.0787	0.0758	0.0951
TCARI	350, 750	0.6912	0.6534	0.6544	0.6381	0.0223	0.0244	0.0233	0.0236
SAVI	450, 650, 950	0.8894	0.9117	0.9253	0.8816	0.0301	0.0274	0.0250	0.0312
SR800550	350, 550	0.9289	0.8843	0.8941	0.9199	0.9059	1.1885	1.1029	0.9586
CIIRE	350, 650, 1050	0.7518	0.6924	0.7712	0.7005	0.1931	0.2172	0.1907	0.2091
LCI	350, 450, 650, 2450	0.9514	0.9564	0.9593	0.9472	0.0175	0.0166	0.0161	0.0186
ARI	550, 750	0.9551	0.9398	0.8467	0.9133	0.7909	0.9229	1.4548	1.0956
SPRI2	550, 650	0.9432	0.9088	0.8663	0.9355	0.6196	0.7911	0.9501	0.6562
BRI	650, 1350	0.6473	0.4888	0.6036	0.5873	0.0752	0.0921	0.0778	0.0803
SPADI	350, 650	0.9823	0.9820	0.9906	0.9779	0.0070	0.0075	0.0053	0.0085
GM1	550, 650	0.9452	0.9131	0.8711	0.9393	0.6218	0.7902	0.9516	0.6512
RVI1	350, 450, 650, 950	0.7956	0.7739	0.8163	0.7761	0.3105	0.3281	0.3023	0.3231
CRİ1	450, 850	0.9056	0.9031	0.7890	0.9085	0.9944	1.0043	1.4787	0.9894
RVSI	750, 850	0.9840	0.9835	0.9848	0.9795	0.0084	0.0084	0.0084	0.0098
NDVI1	650, 1050	0.9568	0.9640	0.9774	0.9528	0.0240	0.0220	0.0175	0.0259
NDVI3	350, 650, 2150	0.8273	0.8117	0.8465	0.8425	0.0229	0.0239	0.0218	0.0221
NDVI6	350, 650, 1050	0.9236	0.9373	0.9463	0.9059	0.0239	0.0217	0.0201	0.0265
PSNDB	650, 1050	0.9730	0.9773	0.9848	0.9663	0.0164	0.0151	0.0123	0.0193
LIC2	650, 1050	0.9692	0.9750	0.9858	0.9648	0.0183	0.0166	0.0125	0.0205

Results for 100 nm first dehydration stage

VIs	Inputs	R^2				$RMSE$			
		RF	MLP	SVR	kNN	RF	MLP	SVR	kNN
TM57	1050, 2150, 2250, 2450	0.9751	0.9839	0.9803	0.9771	0.0274	0.0221	0.0249	0.0285
FWBI	750, 2150	0.8914	0.9020	0.9106	0.9029	0.0029	0.0028	0.0026	0.0028
LWI	1050, 1450, 2050, 2150	0.9753	0.9962	0.9838	0.9850	0.0300	0.0118	0.0254	0.0252
SIWSI	1050, 1450	0.8095	0.8075	0.8671	0.8302	0.0143	0.0147	0.0124	0.0137
VOG1	450, 550, 750, 1950, 2150	0.8803	0.8519	0.8539	0.8682	0.0358	0.0400	0.0394	0.0376
SIPI	350, 850	0.2864	0.4303	0.3573	0.4475	0.0091	0.0082	0.0083	0.0081
VOPT	450, 650, 950	0.8986	0.9075	0.9321	0.9074	0.1083	0.1067	0.0911	0.1058
TCARI	750	0.6325	0.5968	0.7300	0.6940	0.0229	0.0238	0.0193	0.0204
SAVI	450, 650, 950	0.9248	0.9357	0.9566	0.9339	0.0238	0.0230	0.0188	0.0231
SR800550	550, 1050	0.9760	0.9771	0.9339	0.9724	0.4094	0.4046	0.6878	0.4719
CIIRE	450, 550, 650, 750, 2450	0.9043	0.8973	0.8741	0.8977	0.0995	0.1051	0.1132	0.1037
LCI	450, 550, 650, 1050, 2350	0.9638	0.9700	0.9670	0.9531	0.0172	0.0162	0.0167	0.0200
ARI	550, 1050	0.9570	0.9634	0.8581	0.9491	0.5338	0.5076	0.9716	0.5956
SPRI2	550, 1050	0.9582	0.9611	0.9327	0.9593	0.3912	0.3810	0.4990	0.4029
BRI	350, 1350	0.6564	0.5277	0.6286	0.6195	0.0669	0.0768	0.0676	0.0688
SPADI	350, 650, 1050	0.9907	0.9993	0.9987	0.9785	0.0064	0.0016	0.0022	0.0111
GM1	550, 1050	0.9628	0.9634	0.9329	0.9601	0.3830	0.3836	0.5185	0.4134
RVI1	550, 1050, 2050	0.9145	0.9158	0.9180	0.9010	0.1501	0.1586	0.1472	0.1631
CRİ1	450	0.9680	0.9438	0.8587	0.9745	0.4556	0.6168	0.9646	0.4078
RVSI	750, 850	0.9538	0.9624	0.9715	0.9549	0.0066	0.0063	0.0051	0.0067
NDVI1	450, 650, 1050	0.9785	0.9837	0.9837	0.9771	0.0193	0.0174	0.0170	0.0213
NDVI3	450, 550, 650, 750, 1950, 2150	0.9037	0.8724	0.8870	0.9028	0.0188	0.0217	0.0203	0.0190
NDVI6	550, 650, 1050	0.9706	0.9722	0.9741	0.9660	0.0158	0.0160	0.0149	0.0180
PSNDB	450, 550, 650, 1250	0.9804	0.9918	0.9883	0.9818	0.0159	0.0105	0.0124	0.0166
LIC2	450, 650, 1050	0.9833	0.9887	0.9894	0.9824	0.0155	0.0135	0.0125	0.0175

Results for 100 nm second dehydration stage

VIs	Inputs	R^2				$RMSE$			
		RF	MLP	SVR	kNN	RF	MLP	SVR	kNN
TM57	2250, 2350	0.9502	0.9194	0.9569	0.9526	0.0325	0.0421	0.0303	0.0322
FWBI	750, 1450	0.8389	0.8447	0.8876	0.8547	0.0032	0.0032	0.0027	0.0031
LWI	1450, 2050	0.9560	0.9397	0.9620	0.9631	0.0328	0.0396	0.0312	0.0303
SIWSI	1050, 1450, 1950	0.7556	0.7017	0.7870	0.7156	0.0136	0.0152	0.0130	0.0145
VOG1	450, 550, 650, 750, 1950, 2450	0.8493	0.8165	0.8626	0.8681	0.0359	0.0403	0.0344	0.0341
SIPI	650, 950	0.5369	0.3539	0.3082	0.4517	0.0075	0.0086	0.0083	0.0078
VOPT	450, 650, 950	0.9313	0.9378	0.9576	0.9439	0.0973	0.0923	0.0765	0.0982
TCARI	750, 850	0.5064	0.5409	0.6624	0.5685	0.0277	0.0271	0.0240	0.0260
SAVI	450, 650, 1150	0.9306	0.9378	0.9640	0.9355	0.0255	0.0242	0.0184	0.0279
SR800550	350, 450, 550, 650, 1150	0.9872	0.9914	0.9052	0.9676	0.2874	0.2317	0.9314	0.4648
CIIRE	550, 650, 2450	0.8743	0.8506	0.8653	0.8799	0.1014	0.1159	0.1058	0.1003
LCI	550, 2350	0.9589	0.9446	0.9676	0.9506	0.0194	0.0224	0.0171	0.0226
ARI	350, 550, 1050	0.9660	0.9469	0.8518	0.9412	0.4495	0.5572	0.9828	0.5917
SPRI2	350, 450, 550, 650, 1150	0.9834	0.9867	0.9056	0.9654	0.2358	0.2092	0.6626	0.3487
BRI	350, 1250	0.4166	0.2440	0.4263	0.3358	0.0769	0.0909	0.0738	0.0786
SPADI	450, 650, 1050	0.9837	0.9978	0.9985	0.9820	0.0094	0.0034	0.0027	0.0137
GM1	350, 450, 550, 650, 1150	0.9839	0.9873	0.9046	0.9669	0.2434	0.2150	0.6945	0.3586
RVI1	550, 1950	0.9138	0.8725	0.8898	0.9036	0.1384	0.1714	0.1563	0.1507
CRİ1	350, 450, 1150	0.9818	0.9798	0.8147	0.9389	0.3927	0.4057	1.2890	0.7064
RVSI	750, 850	0.9574	0.9660	0.9721	0.9670	0.0060	0.0056	0.0049	0.0054
NDVI1	450, 650, 1050	0.9776	0.9857	0.9832	0.9786	0.0220	0.0177	0.0198	0.0268
NDVI3	450, 550, 650, 750, 2450	0.8853	0.8990	0.8986	0.8863	0.0199	0.0189	0.0188	0.0201
NDVI6	550, 650, 1050	0.9665	0.9498	0.9691	0.9579	0.0184	0.0224	0.0177	0.0225
PSNDB	350, 650, 1050	0.9777	0.9888	0.9860	0.9571	0.0194	0.0133	0.0153	0.0283
LIC2	450, 650, 1050	0.9809	0.9897	0.9890	0.9815	0.0187	0.0139	0.0144	0.0241

Results for 100 nm third dehydration stage

VIs	Inputs	R^2				$RMSE$			
		RF	MLP	SVR	kNN	RF	MLP	SVR	kNN
TM57	1350, 2250	0.9554	0.9594	0.9708	0.9432	0.0220	0.0213	0.0177	0.0269
FWBI	750	0.8559	0.8714	0.8846	0.8584	0.0027	0.0027	0.0024	0.0026
LWI	2050, 2450	0.8065	0.7506	0.8242	0.8036	0.0179	0.0217	0.0170	0.0179
SIWSI	750, 850	0.5827	0.5397	0.6830	0.6345	0.0113	0.0117	0.0099	0.0105
VOG1	350, 550, 650	0.9088	0.8978	0.9052	0.9111	0.0176	0.0196	0.0179	0.0174
SIPI	650, 950	0.4687	0.3703	0.5561	0.4970	0.0119	0.0137	0.0112	0.0116
VOPT	450, 650, 950	0.9408	0.9507	0.9679	0.9502	0.0839	0.0782	0.0619	0.0808
TCARI	850, 1050, 1950	0.5440	0.4332	0.5751	0.5403	0.0313	0.0355	0.0304	0.0317
SAVI	450, 650, 950	0.9528	0.9620	0.9761	0.9575	0.0212	0.0194	0.0151	0.0214
SR800550	350, 450, 550, 650, 1050	0.9791	0.9824	0.9352	0.9677	0.2333	0.2165	0.4129	0.2937
CIIRE	350, 550, 650	0.9290	0.9020	0.9066	0.9222	0.0511	0.0625	0.0587	0.0535
LCI	550, 650, 1950	0.9666	0.9628	0.9637	0.9518	0.0171	0.0181	0.0183	0.0213
ARI	350, 450, 550, 1250	0.9658	0.9654	0.9092	0.9435	0.2845	0.2901	0.4710	0.3657
SPRI2	350, 450, 550, 650, 950	0.9748	0.9830	0.9373	0.9635	0.1839	0.1539	0.2920	0.2257
BRI	350, 450, 2250	0.6393	0.4517	0.6308	0.6330	0.0609	0.0751	0.0613	0.0622
SPADI	650, 1250	0.9810	0.9878	0.9914	0.9754	0.0108	0.0087	0.0073	0.0140
GM1	350, 450, 550, 650, 950	0.9747	0.9828	0.9361	0.9642	0.1936	0.1622	0.3104	0.2345
RVI1	350, 450, 550, 650, 1950	0.9490	0.9542	0.9206	0.9217	0.0731	0.0704	0.0912	0.0902
CRİ1	350, 650, 950	0.9391	0.9145	0.8719	0.9174	0.3984	0.4731	0.5762	0.4628
RVSI	750, 850	0.9842	0.9869	0.9903	0.9810	0.0034	0.0034	0.0026	0.0038
NDVI1	650, 1050	0.9705	0.9649	0.9736	0.9655	0.0248	0.0271	0.0235	0.0287
NDVI3	550, 750, 1950	0.9381	0.9288	0.9289	0.9161	0.0119	0.0133	0.0128	0.0145
NDVI6	650, 1950	0.9601	0.9457	0.9527	0.9448	0.0194	0.0226	0.0211	0.0236
PSNDB	650, 1950	0.9739	0.9670	0.9706	0.9653	0.0201	0.0231	0.0214	0.0257
LIC2	650, 1250	0.9741	0.9700	0.9787	0.9673	0.0219	0.0235	0.0199	0.0267

Results for 100 nm fully dry stage

VIs	Inputs	R^2				$RMSE$			
		RF	MLP	SVR	kNN	RF	MLP	SVR	kNN
TM57	750, 950, 1750, 2150, 2250	0.9956	0.9974	0.9856	0.9914	0.0276	0.0220	0.0501	0.0386
FWBI	750, 850, 1150, 1850	0.9932	0.9965	0.9946	0.9912	0.0019	0.0014	0.0018	0.0022
LWI	850, 950, 1550, 1650, 1750, 2250	0.9971	0.9996	0.9929	0.9963	0.0313	0.0112	0.0485	0.0351
SIWSI	850, 1850	0.9974	0.9963	0.9959	0.9974	0.0085	0.0101	0.0106	0.0084
VOG1	650, 850, 1250, 1750	0.9693	0.9685	0.9523	0.9687	0.0534	0.0543	0.0667	0.0539
SIPI	550, 650, 1050, 1950	0.7585	0.7213	0.7186	0.7418	0.0080	0.0090	0.0087	0.0082
VOPT	650, 850, 2350	0.9941	0.9953	0.9858	0.9931	0.0646	0.0589	0.0983	0.0695
TCARI	350, 1150, 1250, 1650, 1950, 2250	0.8283	0.7855	0.7974	0.8200	0.0274	0.0312	0.0295	0.0281
SAVI	450, 650, 850, 2350	0.9930	0.9941	0.9954	0.9907	0.0121	0.0109	0.0096	0.0141
SR800550	350, 450, 550, 650, 1050, 2350	0.9839	0.9837	0.8929	0.9680	0.3979	0.3987	1.0425	0.5584
CIIRE	650, 850, 1750	0.9668	0.9620	0.9423	0.9672	0.1812	0.1948	0.2448	0.1800
LCI	450, 550, 650, 1050, 2350	0.9798	0.9754	0.9727	0.9753	0.0171	0.0189	0.0200	0.0188
ARI	350, 550, 750	0.9715	0.9517	0.8660	0.9625	0.5879	0.7676	1.2533	0.6729
SPRI2	450, 550, 650, 950, 2350	0.9822	0.9818	0.8998	0.9705	0.3623	0.3652	0.8657	0.4655
BRI	350, 850, 1450	0.8891	0.8406	0.8729	0.8878	0.0753	0.0919	0.0805	0.0759
SPADI	650, 950, 2450	0.9924	0.9983	0.9970	0.9866	0.0065	0.0030	0.0040	0.0093
GM1	450, 550, 650, 950, 2350	0.9833	0.9813	0.8992	0.9712	0.3486	0.3693	0.8635	0.4563
RVI1	450, 650, 750, 2150	0.9706	0.9695	0.9463	0.9711	0.2870	0.2993	0.4021	0.2850
CRİ1	450, 650, 850, 1250	0.9838	0.9681	0.8572	0.9700	0.6042	0.8909	1.7615	0.8211
RVSI	650, 750, 850, 1250	0.9959	0.9963	0.9957	0.9940	0.0080	0.0077	0.0082	0.0097
NDVI1	650, 950, 2350	0.9867	0.9790	0.9826	0.9809	0.0183	0.0229	0.0208	0.0225
NDVI3	450, 650, 950, 1750, 2150, 2350	0.9554	0.9586	0.9531	0.9540	0.0242	0.0234	0.0247	0.0245
NDVI6	450, 650, 950, 2350	0.9790	0.9793	0.9718	0.9718	0.0225	0.0231	0.0260	0.0260
PSNDB	450, 550, 650, 950, 2350	0.9885	0.9948	0.9877	0.9850	0.0161	0.0108	0.0171	0.0185
LIC2	650, 950, 2350	0.9897	0.9877	0.9889	0.9835	0.0142	0.0155	0.0148	0.0189

Results for 100 nm combined stages

References
